# Global Justice Index Report 2020

**DOI:** 10.1007/s41111-021-00178-1

**Published:** 2021-05-06

**Authors:** Yanfeng Gu, Xuan Qin, Zhongyuan Wang, Chunman Zhang, Sujian Guo

**Affiliations:** Fudan Institute for Advanced Study in Social Sciences, Fudan University, Shanghai, China

**Keywords:** Global Justice Index, Indicators, Measurements, Methods, Country, Global rankings

## Abstract

The Global Justice Index is a multiyear research project conducted at the Fudan-IAS to conceptualize and measure each country’s contribution to achieving greater global justice. In 2019, we completed our research project on first-year achievements, with the rankings of nation-states at the global level based on data from 2010 to 2017. This was published titled the “Global Justice Index Report” in *Chinese Political Science Review* (Vol. 5, No. 3, 2020). The “Global Justice Index Report 2020” is the second annual report based on our work analyzing data from 2010 to 2018, which was concluded in 2020. In order to better measure each country’s performance and contribution to achieving greater global justice, compared to the first edition published in 2020, we have improved the model, added the refugee issue to expand the issue areas to 10, and added new indicators, regional analysis and comparison in this report. The report comprises five main sections. In the introduction, we discuss the development of the conceptual framework and evaluative principles to justify our selection of dimensions and indicators for measurement. Next, in the section of methodology, we discuss the production, normalization, and aggregation of the raw data and the generation of the final results. In the findings section, we report the data, indicators and our results for the ten issues, and provide regional comparisons. And then, in the following section we present the main results, and report the ranking of each country’s contribution to achieving greater global justice. In the final section, we discuss the applications and limitations of the index, and its potential further research trajectories.

## Introduction

The Global Justice Index is a multiyear research project conducted at the Fudan-IAS to conceptualize and measure each country’s contribution to achieving greater global justice. In 2019, we provided our first-year achievements with the rankings of nation-states at the global level from 2010 to 2017. Based on the results, we have published a book in Chinese and an academic paper in English, which has received widespread attention. Building on the success of the previous year’s work, in 2020, we intend to provide our second-year results with the rankings of nation-states at the global level from 2010 to 2018. This year’s Global Justice Index (2020) report consists of five sections: introduction, methodology, results, analysis and conclusion.

In the introduction, we highlight our theoretical innovation by discussing the development of the conceptual framework to justify our selection of issues, dimensions and indicators for measurement. In addition, we present some major changes in this year’s report compared with last year’s report. Next, in our methodology section, we introduce our methods for production, normalization, and aggregation of the raw data and the generation of the final results. In the results section, we present the rankings of nation-states’ contribution to global justice from 2010 to 2018. Following the results section, we provide regional comparisons with detailed policy analysis assisted with various visualization tools. In the last concluding section, we discuss the applications and limitations of the index, and its potential further research trajectories and policy implications for advancing global justice.

Global justice is a broad concept composed of multilevel and multidimensional aspects belonging to both normative and empirical realities. A coherent, integrated theoretical framework that covers the normative basis and various empirical dimensions is therefore necessary to address some of the basic and important questions under study. Our Global Justice Index study began with the conceptualization of global justice based on a theoretical paper titled “Conceptualizing and Measuring Global Justice: Theories, Concepts, Principles and Indicators,” coauthored by the project leader, Sujian Guo et al. published in *Fudan Journal of the Humanities and Social Sciences* (Vol. 12, No. 4, 2019). The paper discusses theories, concepts, evaluative principles, and methodologies related to the study of global justice.

In the theoretical paper above (Guo et al. 2019), we attempt to clarify how to conceptualize global justice, how conceptual indicators can be selected and justified by theories, and how those indicators can be consistent with the concept of global justice. Through the synthesis of multiple theories and intellectual traditions in various cultural and political contexts, we conceptualize global justice from three main approaches—rights based, goods based, and virtue based—to develop a normatively based theoretical framework for measurement. Rights-based conceptualization focuses on the basic principles, rules, and sources of legitimacy of justice (*Universal Declaration of Human Rights*, 1948; Rawls, 1971, 1999). Goods-based conceptualization concentrates on the material and institutional supports that governments or institutions are obliged to provide (Arneson, 1989; Freeman, 2006; Nussbaum, 2006, 2011; Richardson, 2006). And virtue-based conceptualization regards justice as a virtue that an individual is willing to pursue rather than a regulation an individual is forced to comply with (Mo, 2003). The relationship between the three approaches of conceptualization is interdependent rather than separate, which indicates three interrelated components of a holistic whole. Additionally, the three approaches are complementary rather than competing, with the rights-based conceptualization forming the basic structure (“the bones”), the goods-based conceptualization providing substantial material supports (“the muscles”, and the virtue-based conceptualization emphasizing personal motivation and internalized willingness (“the heart)” (Guo et al., 2019).

Based on the aforementioned theoretical framework, we propose two evaluative principles to further bridge the gap between theory and practice to determine and justify our selection of issue areas for evaluation. We call the two principles Common but Differentiated and Respective Capabilities (CBDR-RC) and Cosmopolitan but Due-diligent Responsibilities (CDDR). CBDR-RC addresses the issues “for which no single nation-state can be held directly accountable or responsible, matters that can only be tackled through the globally concerted efforts of all stakeholders” (Guo et al. 2019). For example, it is the responsibility of all to protect the climate system and ecological balance, and environmental protection is a task that cannot be handled by one country on its own. The principle of CBDR-RC, first adopted by the United Nations Framework Convention on Climate Change and reaffirmed in the Rio Declaration on Environment and Development, combines normative legitimacy and historical rationality. Although it was a principle that first aimed to determine the responsibilities of each country for climate change, it has been expanded to other global justice areas such as combatting transnational crime and global peacekeeping.

The second principle, CDDR, addresses that “all-nation-states are morally obligated to provide cosmopolitan aid, in which context the least advantaged will have a due-diligent responsibility” (Guo et al. 2019). This principle is based on the concept of “mutual accountability” proposed in the Paris Declaration on Aid Effectiveness, adopted in 2005 at the Second High-Level Forum on Aid Effectiveness to promote better cooperation between different actors in aid and development. This principle views such obligations as part of domestic affairs, such as anti-poverty and education policy, in the context of which nation-states are expected to provide material and institutional assistance to their citizenry within their territories.

According to the principles of CBDR-RC and CDDR, we have selected two clusters of global justice issue areas for practical measurement. Those issue areas that follow the principle of CBDR-RC are (1) climate change (global warming), (2) peacekeeping, (3) humanitarian aid, (4) terrorism and armed conflicts, (5) cross-national criminal police cooperation, (6) refugees; and those belonging to the principle of CDDR are (7) anti-poverty, (8) education, (9) public health and (10) the protection of women and children.

This year’s Global Justice Index study is not simply a continuation of last year’s work. To further improve the quality of our index, we have made a few major modifications. First of all, we have perfected our selection of issue areas and indicators by adding a brand new issue area and more indicators to our study. In the Global Justice Index (2019), we have selected nine issues areas to construct the index. The issue of refugees has been included in this year’s Global Justice Index as more and more attention has been devoted to the fermenting refugee crisis. For other issue areas, indicator systems have been either kept unchanged or improved. Second, we have slightly modified our research methodology to better calculate the index (for more information, please see the next section). Third, we have changed our indicators and included more data in our calculation. Last, we have strengthened our analysis section by incorporating and discussing more literature and policy implications. As such, readers from different backgrounds can all benefit.

Due to these new changes, readers may find that some countries’ rankings in this year’s Global Justice Index are quite different from those in the Global Justice Index (2019), while other countries’ rankings have not changed substantially. This should not be a surprise to our readers. Global justice is a cutting-edge research field which involves sophisticated materials, a large volume of data and a changing international landscape. We aim to keep our results consistent across different years. At the same time, we do make necessary and important modifications to our research design in light of a changing international environment and the availability of new and better data.

## Methodology: Construction of the Global Justice Index

In this study, we classify our data into four levels: indicators, dimensions, categories, and issues. The first and lowest level of our data provides the information on indicators, which is our raw data. The second level is named dimensions, which usually comprises several related indicators. The third level is categories and comprises several related dimensions. And the highest level is the issue index, usually calculated based on two categories: contribution and performance.

The global justice index is calculated as follows:

### First step: Convert Indicator Indices

To ensure comparability between indicators, we use the following two formulas to convert the raw data into comparable indicators:

1 $$\begin{array}{c}i{i}_{ij}=\frac{{actual value}_{ij}-\mathrm{min}({actual value}_{.j})}{\mathrm{max}({actual value}_{.j})-\mathrm{min}({actual value}_{.j})}+1\end{array}$$

2 $$\begin{array}{c}i{i}_{ij}=\frac{\mathrm{max}({actual value}_{.j})-actual valu{e}_{ij}}{\mathrm{max}({actual value}_{.j})-\mathrm{min}({actual value}_{.j})}+1\end{array}$$

$$i\in \left\{\mathrm{1,2},\cdots 192\right\} , j\in \left\{\mathrm{2010,2011},\cdots 2018\right\},$$

where $actual {value}_{ij}$ indicates the actual value of an indicator in country *i* in year *j*, $\mathrm{min}({actual value}_{.j})$ is the minimum value of an indicator among all countries in year *j* and $\mathrm{max}({actual value}_{.j})$ is the maximum value of an indicator among all countries in year *j*. If an indicator positively relates to global justice, the first formula is used to convert the raw data; if not, the second formula is used.

### Second step: Population-Based Weighting

Consciously, efforts made to raise the welfare of their populations to the same level have a comparatively larger overall impact in countries with larger populations; therefore, we weight indicators based on population size. We proceed as follows:

First, we calculate the weighted average of an indicator as per the following formula:

3 $${ii}_{mj}=\frac{\sum {ii}_{ij}*{population}_{ij}}{\sum {population}_{ij}}.$$

where ${ii}_{mj}$ is the weighted average of an indicator. $i{i}_{ij}$ is the actual value of an indicator in country *i* in year *j.*$populatio{n}_{ij}$ is the population size of country *i* in year *j*.

Second, we calculate the weight of each country on an indicator as follows:

4 $${ss}_{ij}=\left(i{i}_{ij}-{ii}_{mj}\right)*populatio{n}_{ij},$$

where ${ss}_{ij}$ *i*s the weight of country *i* in year *j.*

Third, we calculate the score for an indicator in country* i* in year *j* as follows:

5 $${II}_{ij}=\frac{{ss}_{ij}-\mathrm{min}({ss}_{.j})}{\mathrm{max}\left({ss}_{.j}\right)-\mathrm{min}({ss}_{.j})}+1,$$

where ${II}_{ij}$ is the score of an indicator in country *i* in year *j.* We use ${II}_{ij}$ to further calculate the dimension global justice.

### Third step: Calculate the Scores of Both Dimension Indices and Category Indices

For each variable, we calculate the score of the dimension index as follows:

6 $${DI}_{ij}=\frac{1}{k}\sum {II}_{ijk} ,$$

where ${II}_{ij}$ is the score of an indicator in country *i* in year *j*, and K is the number of indicators in a specific dimension in country *i* in year *j.*

Similarly, we use ${DI}_{ij}$ to further calculate the score of category indices as follows:

7 $${VI}_{ij}=\sqrt[n]{\prod_{k}D{I}_{ijk}}$$

### Fourth step: Calculate the Score of the Issue Index

We use ${VI}_{ij}$ to further calculate the score of each issue in county *i* in year *j* as follows:

8 $${ISI}_{ij}=\sqrt[n]{\prod_{k}V{I}_{ijk}}$$

### Last step: Calculate Global Justice Index

We use the following formula to calculate the score of the global justice index in country *i* in year *j*:

9 $${GJ}_{ij}=\sqrt[10]{\prod_{k}{ISI}_{ijk}} ,$$

where ${GJ}_{ij}$ is the score of global justice in country *i* and year *j*.${ISI}_{ijk}$ is the score of issue *k* in country *i* in year *j.*

## Findings

### Issue 1: Climate Change

#### Introduction

Nowadays, more and more people around the globe have realized that climate change is a global challenge facing our planet, closely related to the survival of human beings and the continuation of our civilization. Climate change will bring about many problems, such as extreme weather, melting ice and snow, rising sea levels, frequent mountain fires and so on. Climate change has been a feature of the evolution of the earth itself, and it has long been a completely natural phenomenon, without significant human intervention. But with the development of mankind, more and more human activities have been linked to climate change. For example, the industrial production process needs to consume a substantial amount of fossil energy, which causes a lot of greenhouse gases to be emitted into the air, further enhancing global warming. Many activities in our daily lives are also emitting greenhouse gases into the air. The increase in the earth's temperature caused by climate change will have an irreversible impact and cause harm to human production and life, so all countries in the world must act.

The issue of climate change is also a global justice issue, because it involves the distribution of responsibilities and obligations between all of the developing and developed countries in the world. The signing of the Paris Climate Agreement in 2015 was a milestone event for dealing with the climate change issue, which shows the strong determination of governments of all countries to cooperate to solve the problem of global warming. However, the Trump administration’s withdrawal from the Agreement in recent years has cast a shadow on global climate governance. With the victory of Joe Biden in the 2020 US election, the United States is very likely to return to the field of global climate governance and even push countries to take more measures to deal with climate change. The Climate Ambition Summit which was held in December 2020 shows a new surge in action and ambition to control global warming. As a matter of fact, countries are taking various measures to advance their national determined contribution targets, but how well each country is doing in reality remains unknown. Our Global Justice Index research will answer this question through data analysis.

#### Dimensions and Indicators

The issue of global warming has prompted worldwide discussion. At the beginning of this century, Thomas Crowley published an important research paper in *Science*, arguing that “natural variability plays only a subsidiary role in the twentieth-century warming and that the most parsimonious explanation for most of the warming is that it is due to the anthropogenic increase in GHG”.^1^ This research finding is consistent with the definition of climate change in the *United Nations Framework Convention on Climate Change* which defines climate change as a change of climate which is attributed directly or indirectly to human activity that alters the composition of the global atmosphere and which is in addition to natural climate variability observed over comparable time periods. According to these scientific research results and the definition of climate change of the United Nations, our empirical analysis for measuring countries’ contributions to solving climate change includes four dimensions: energy consumption, electricity production, CO2 and forests.

We have obtained highly reliable open source data from prestigious international organizations, research institutions, and multinational companies, such as forest data from the United Nations Environment Programme, and carbon dioxide-related data from the Global Carbon Project. Based on these open source data, we design three to five indicators for each dimension. For example, in the energy consumption dimension, our indicators include primary energy consumption in total, primary energy consumption per capita, oil consumption, natural gas consumption, and coal consumption. In the electricity production dimension, our indicators include electricity production in total, electricity production from nuclear sources, electricity production from hydroelectric sources, Electricity production from renewable sources excluding hydroelectric. In the dimension of CO2, our indicators include C02 emissions, C02 emissions per GDP and C02 emissions per capita. In the forest dimension, our indicators include forest area in total, forest area change rate, forest area per capita, forest coverage, planted forest area. In order to better reflect the contributions of countries around the world in the current battle against climate change in the past decade, the time span that we focus on in this project is from 2010 to 2018. Last year's Global Justice Index research on climate change covers 192 countries around the world, but the time frame is from 2010 to 2014. This year we have done a better job in terms of time frame than last year, but this year's research can only cover 75 countries, which leaves some small and medium-sized countries omitted. We are fully aware that the scale of national coverage is a major shortcoming of this year’s research due to the lack of reliable data, but the comprehensive indicator system can make sure that we can have a sound calculation of 75 countries’ contributions to global justice from the perspective of fighting climate change. We will keep looking for better and comprehensive data to cover more countries in the future (Table 1).

**Table 1:** Data on climate change

Category	Dimension	Indicator	Data source	Coverage
Performance	Energy consumption	Primary energy consumption in total	BP Statistical Review of World Energy	75 2010–2018
		Primary energy consumption per capita		
		Oil consumption		
		Natural gas consumption		
		Coal consumption		
	Electricity production	Electricity production in total		
		Electricity production from nuclear sources		
		Electricity production from hydroelectric sources		
		Electricity production from renewable sources excluding hydroelectric		
	CO_2_	C02 emissions	Global Carbon Project	192 2010–2018
		C02 emissions per GDP		
		C02 emissions per capita		
	Forest	Forest area in total	UN Environment Programme	192 2010–2018
		Forest area change rate		
		Forest area per capita		
		Forest coverage		
		Planted forest area		

#### Results

In this section, we present the ranking results of the countries’ contributions to global justice from a climate change perspective (Table 2). Table 2 shows 9 years of results from 2010 to 2018 in 75 countries.

**Table 2:** Country ranking in the climate change aspect of promoting global justice

Country	2010	2011	2012	2013	2014	2015	2016	2017	2018
Brazil	1	1	1	1	1	1	1	1	1
Canada	2	2	2	2	2	2	2	2	2
Sweden	3	3	3	3	3	3	3	3	3
Russian Federation	4	4	4	4	4	4	4	4	4
China	18	18	16	12	10	7	5	5	5
France	6	5	5	5	5	5	6	6	6
Finland	9	7	6	7	6	6	7	7	7
Peru	7	6	7	6	7	8	8	8	8
Colombia	10	11	10	9	9	11	11	9	9
Philippines	36	9	9	8	8	9	9	10	10
Japan	5	8	15	17	15	13	12	12	11
Latvia	13	14	13	13	14	14	13	13	12
Viet Nam	12	16	14	14	16	16	16	14	13
United States of America	14	10	8	11	11	10	10	11	14
Spain	8	12	11	10	12	15	14	15	15
Chile	34	20	20	20	19	18	17	17	16
Slovenia	22	25	24	24	20	19	19	18	17
Germany	11	13	12	15	13	12	15	16	18
Malaysia	17	22	22	26	22	21	22	19	19
Indonesia	15	17	19	16	17	17	18	20	20
Romania	35	31	30	25	23	23	21	22	21
Ecuador	19	21	18	21	26	24	23	21	22
Switzerland	30	29	29	30	28	27	26	25	23
Sri Lanka	21	24	25	23	27	28	28	26	24
Italy	28	23	21	19	18	20	20	24	25
New Zealand	23	27	28	28	29	29	25	29	26
Austria	25	28	26	27	25	26	27	28	27
India	16	15	17	18	21	22	24	23	28
Norway	24	26	27	29	30	30	29	27	29
Lithuania	29	34	33	32	31	33	31	31	30
Venezuela (Bolivarian Republic of)	20	19	23	22	24	25	30	30	31
Azerbaijan	27	30	31	31	33	34	33	32	32
Mexico	32	33	35	34	35	35	34	34	33
United Kingdom of Great Britain and Northern Ireland	48	40	40	39	37	32	32	33	34
Slovakia	39	38	36	37	36	36	37	35	35
Portugal	31	32	34	33	34	37	36	37	36
Thailand	38	37	38	38	39	41	40	38	37
Turkey	37	36	37	36	38	38	38	39	38
Republic of Korea	33	35	32	35	32	31	35	36	39
Bulgaria	43	46	43	40	43	45	42	43	40
Greece	44	42	44	43	41	40	39	41	41
Belarus	42	39	39	41	40	39	41	40	42
Denmark	47	44	42	44	44	42	43	42	43
Hungary	41	41	41	42	42	43	44	44	44
Bangladesh	45	43	46	45	47	48	46	46	45
Poland	46	45	45	46	45	44	45	45	46
Australia	54	47	47	47	46	46	47	47	47
Czechia	50	50	48	49	48	49	49	48	48
Ireland	51	49	50	50	49	50	50	49	49
Morocco	40	48	49	48	50	51	48	50	50
Israel	59	56	56	54	52	53	53	53	51
Egypt	49	51	54	52	55	54	54	54	52
Cyprus	53	53	53	51	53	55	55	55	53
Belgium	55	52	52	53	51	52	52	51	54
Estonia	56	55	51	56	54	47	51	52	55
Luxembourg	62	62	61	59	57	56	56	56	56
Argentina	52	54	55	55	56	57	58	58	57
Algeria	26	57	57	57	58	58	57	57	58
Ukraine	63	63	63	62	62	60	61	59	59
Netherlands	58	58	58	58	59	59	59	60	60
Uzbekistan	66	67	67	65	65	64	64	63	61
Singapore	64	61	62	63	63	63	60	62	62
Iraq	57	59	60	60	61	61	62	61	63
Iceland	60	60	59	61	60	62	63	64	64
Pakistan	61	64	64	64	64	65	65	65	65
South Africa	67	66	66	67	67	66	67	66	66
Iran (Islamic Republic of)	65	65	65	66	66	67	66	67	67
Oman	68	68	68	68	68	68	68	68	68
Turkmenistan	73	72	72	70	70	71	69	69	69
United Arab Emirates	69	70	70	72	72	70	71	71	70
Kuwait	71	73	73	73	71	72	72	72	71
Saudi Arabia	72	71	71	71	73	73	73	73	72
Kazakhstan	70	69	69	69	69	69	70	70	73
Trinidad and Tobago	75	74	74	74	74	74	74	74	74
Qatar	74	75	75	75	75	75	75	75	75

The table above shows that from 2010 to 2018, Brazil, Canada, Sweden, Russia, France, Finland, Peru, Colombia and other countries have consistently performed well in climate change. Among them, Brazil, Russia, Peru and Colombia are developing countries while Canada, Sweden, France and Finland are developed ones. China's performance in climate change over the previous years has been very impressive, and it is a good model for developing countries. In 2010, China ranked 18th in terms of climate change performance. After that, the progress was very obvious. It entered the top 10 in 2014 and has risen to 5th in 2018. Among the entire 75 countries, developing countries have done worse than developed countries. Saudi Arabia, Kazakhstan, Trinidad and Tobago, Qatar and other countries ranked low. In addition, some countries’ climate change ranking has shown great volatility. For example, the United Kingdom has risen from 48 in 2010 to 34 in 2018, showing a clear upward trend; Algeria has dropped from 26 in 2010 to 58 in 2018, showing a clear downward trend; and Japan first drops and then rises, forming a V-shaped fluctuating change.

Brazil's ranking from 2010 to 2018 is quite stable because its vast forests make it score higher in the forest dimension, and its performance in the other three dimensions is also excellent. Brazil has a vast Amazon forest, however, the forest area change rate in Brazil has consistently been negative from 2010 to 2018. As such, Brazil ranks 5th in the forest dimension score, i.e. not the first in the world. Canada scored higher in the dimensions of forests and electricity generation. However, because it scored slightly lower in the dimensions of carbon emissions and energy consumption, in the end it ranked second after Brazil. Sweden, which ranked third in 2018, scored lower than Canada in the dimensions of forests and electricity generation. Sweden has a higher score in carbon emissions, and has a slightly higher score in energy consumption than Canada. Russia scored very high in the dimensions of forests and electricity generation, and scored low in terms of carbon emissions and energy consumption. Thanks to the rapid development and energy saving and emission reduction policies in the past 10 years, China scored high both in electricity generation and carbon emissions. These significant improvements are key reasons why its ranking rose from 18th in 2010 to 5th in 2018. France has a high score in the dimensions of carbon emissions and electricity generation, and a low score in the dimensions of forests and energy consumption. Contrary to France, Finland has a low score in the dimensions of energy consumption and carbon emissions and has a medium score in the dimension of electricity generation. Finland’s forest dimension score is higher. Peru scored very highly in the energy consumption, carbon emissions, and forest dimensions, but it scored very low on electricity generation. Similarly, Colombia also has higher scores in the dimensions of energy consumption and carbon emissions; but its score in the electricity generation dimension is slightly higher than Peru, and its score in the forest dimension is slightly lower than Peru, thus the overall ranking is lower than Peru.

The lowest ranking countries in the field of climate change are Saudi Arabia, Kazakhstan, Trinidad and Tobago, and Qatar. The major reason for the low ranking of these countries is that they are generally less capable of addressing climate change. Part of the reason comes from their low scores on forests and carbon emissions. Both Saudi Arabia and Qatar are Middle Eastern countries with relatively few forest resources. Although the scores of these two countries in the forest dimension are on the rise, they are still relatively small compared to other countries. Qatar performed worst because of its poor performance in all aspects. Kazakhstan's forest score is also relatively low, and the gap with Saudi Arabia is not very large, but Kazakhstan's performance in carbon emissions and power generation is weaker than Saudi Arabia; thus it ranks lower than Saudi Arabia.

The United States’ rankings over the years have not been in the top 10. The Democratic Party in the United States is more concerned about climate change issues than the Republican Party. During the Obama administration, the United States performed relatively well on climate change. However, after the 2016 U.S. election, Donald Trump became President of the United States. He strongly supported traditional energy, not new energy, and was very indifferent to the issue of climate change. This led to a significant decline in the ranking of the United States in 2018. The British government has attached great importance to climate change issues in the past few years.^2^ Judging from the scores of the four dimensions, the UK is in an upward phase in the four dimensions of electricity generation, carbon emissions, energy and forests. According to this trend, the UK's ranking in the future is expected to rise.

Germany's ranking is in a downward trend at this stage. On the whole, Germany's performance in the three dimensions of power generation, carbon emissions and forests is good. However, as a major manufacturing country, Germany's carbon emissions are relatively large, thus dragging down Germany's overall performance. Germany changed its attitude towards nuclear power generation after the Fukushima nuclear accident in Japan. The Merkel government has gradually shut down more nuclear power generation in the past few years and plans to close all nuclear power plants in the country by 2022. This has caused Germany's score in power generation to drop substantially, which is also an important reason for the decline in Germany's ranking year by year. But in the long run, we are relatively optimistic about Germany's ability to deal with climate change. Germany is a leader and pioneer in developing renewable energy and promoting low-carbon development. The German government believes that climate protection not only provides long-term guarantees for sustainable economic development, but also brings direct benefits to the German economy. Therefore, it has been actively participating in and promoting action against climate change. Looking at recent history, we found that Germany passed the "Energy Utilization and Climate Protection Package" in 2007, and subsequently passed the "Biofuel Oil Ratio Law", the "Renewable Energy Heating Law", and the "Vehicles A series of related legislations including the Purchase Tax Reform Law, which stipulates that the new car purchase tax rate is linked to the size of the vehicle engine and the level of carbon dioxide emissions. Germany also has strong technical strength in the development of wind power generation, and its ranking may show an upward trend in the future.

India's ranking is generally in decline. As the second largest developing country in the world after China, India is facing great pressure on the issue of climate change. India's scores on the three dimensions of energy, carbon emissions and power generation are all falling, and the only growth is in the forest dimension. From 2010 to 2018, India’s economy and population are still growing rapidly, especially since the Modi government came to power, India’s economic development has accelerated. Some studies have found that India has begun to suffer severe impacts from climate change, especially in the agricultural sector.^3^ But India has yet to find a better way and build greater determination to deal with climate change.

The Dutch ranking in climate change has been around 60 for a long time, which may surprise some readers. As a major developed country, the performance of the Netherlands in tackling climate change is far worse than other developed countries and even many developing ones. Our research shows that the Netherlands performs well in the two dimensions of energy and carbon emissions, but its performance in the two dimensions of forests and power generation is poor, which is the main reason for its relatively backward ranking. Electricity generation in the Netherlands is highly dependent on thermal power plants, which rely on a large amount of fossil energy. The situation in Iceland is similar to that in the Netherlands. Iceland is even worse than the Netherlands in the three dimensions of carbon emissions, power generation and energy consumption, but it performs much better than the Netherlands in the forest dimension. As a developed country, Belgium ranks slightly higher than the Netherlands and Iceland, but it is also in a relatively backward position within the group of developed countries. The reason is that from a data point of view, Belgium ranks relatively low in the two dimensions of forests and power generation.

Japan's ranking decline over the past 9 years is mainly related to the leak at the Fukushima nuclear power plant in 2011. After the accident, the abandonment of nuclear power was one of the main response measures of the Japanese government. In May 2011, Japan’s last nuclear power plant in operation, the Hokkaido Tomari Nuclear Power Plant, ceased power generation. In July 2011, the then Prime Minister of Japan proposed the goal of "establishing a society without nuclear power." Because of the reduction in nuclear power, Japan’s score in power generation has fallen. The later prime minister led the formulation of Japan's new energy and environmental strategy, making it clear that Japan's dependence on nuclear power will be zero by 2030. As a result, Japan’s score on the power generation dimension has dropped consistently, leading to a decrease in its ranking. However, in recent years, Japan has seen some new changes in its attitude towards nuclear power, and it has begun to again support a role for nuclear power in the national energy system. This is the main reason why Japan's climate change rankings are beginning to rise.

#### Regional Analysis

**Fig. 1:**
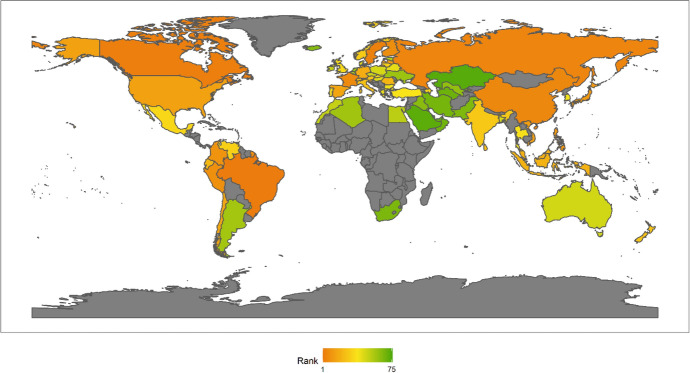
2018 Index ranking of climate change on a world map

In 2018, the top Ten countries in the field of climate change were Brazil, Canada, Sweden, Russia, China, France, Finland, Peru, Colombia, and the Philippines. Among the top ten countries, there are four countries in the Americas, two Asian countries, four European countries, but African countries are not included in the top ten. There are six developing countries and four developed countries (Fig. 1). This shows that the degree of economic development is not necessarily related to the response to climate change. Both developed and developing countries have the opportunity to play an important role in the response to climate change. These countries are able to rank high in the field of climate change because they have some common characteristics. First, these countries generally attach importance to climate change issues. Different countries have different understanding of climate change issues.^4^ For example, the Trump administration of the United States has a very negative attitude towards climate change, and the U.S. government even withdrew from the Paris climate agreement. However, these top 10 countries have not only signed and maintained the Paris climate agreement, but have also been taking many measures to increase their nationally determined contributions. Second, these countries generally have no obvious shortcomings in the four dimensions of energy consumption, carbon emissions, power generation and forests. In other words, the top-ranked countries have performed well in major aspects of tackling climate change. Finally, these countries tend to be particularly prominent in certain aspects of responding to climate change. For example, Russia and Canada have outstanding performance in the forest dimension. China has outstanding performance in the two dimensions of power generation and forests. After signing the Paris Agreement in 2015, China has become more proactive in promoting the transition to low-carbon social and economic development. Renewable energy power generation is developing rapidly in China. China has also invested a lot of money, manpower and material support in afforestation.

It should be noted that the analysis of climate change is based on statistical data of 75 countries, so it reflects the relative ranking of these 75 countries. From an absolute point of view, the lower-ranking countries in the climate rankings may not be worse.

Next, we classify countries according to their continents. These continents include Asia, Europe, North America, Latin America, Africa and Oceania. The ranking of each continent is obtained by calculating the average of the rankings of these countries. We drew a line chart to achieve a visual presentation to compare the differences in the contribution of various continents to climate change.

**Fig. 2:**
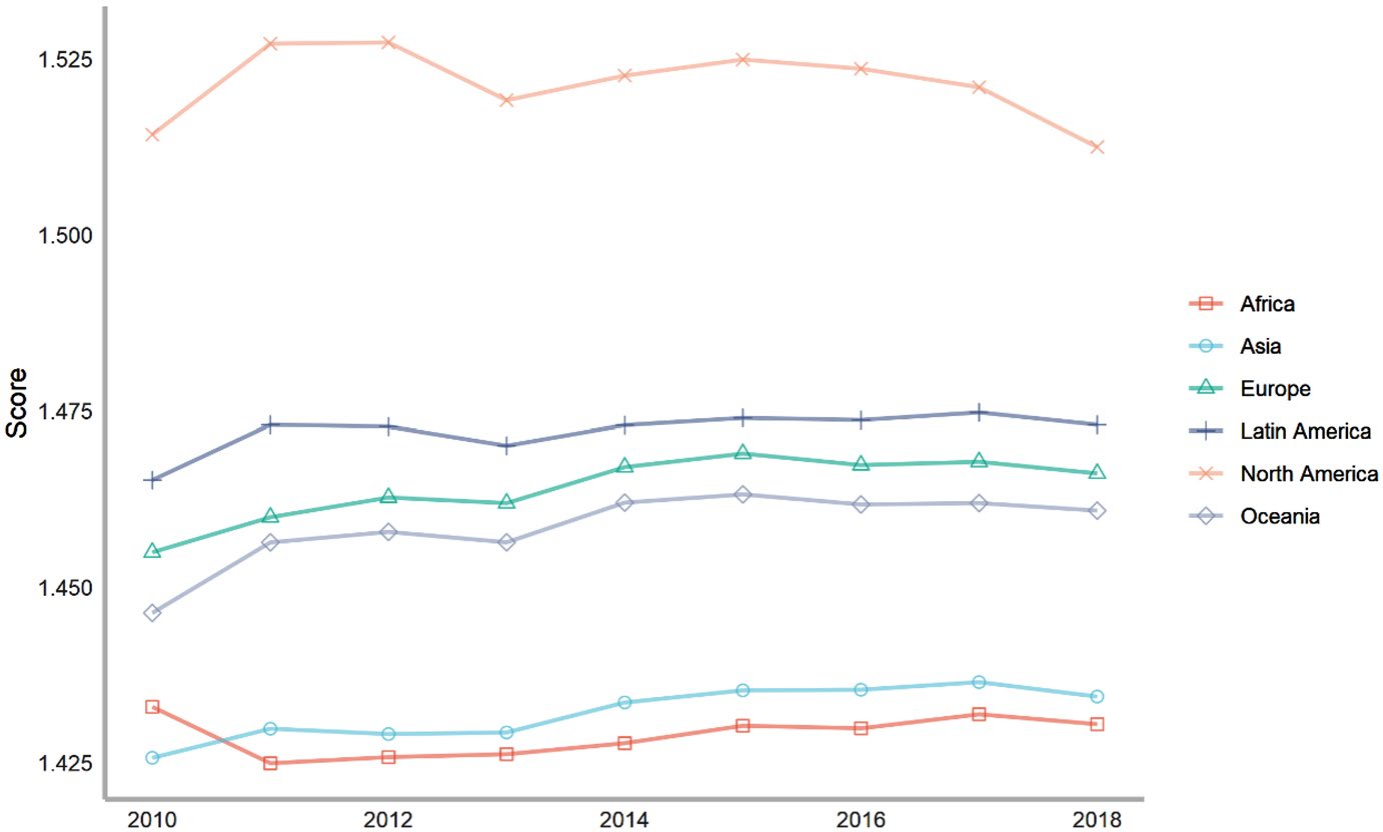
The score of climate change across continents, 2010–2018

Seventy-five countries are included in the six continents, of which there are more countries in Europe and Asia and less countries in Oceania and North America (Fig. 2). According to the average number of scores, the top overall rankings are North American countries, followed by Latin American countries and European countries. The lowest overall ranking is African countries. The reason why North American countries rank high is because North America has only two countries in our ranking, the United States and Canada. The rankings of these two countries are very high. Africa is at the bottom of the ranking because the selected African countries are generally at the bottom, and no country has performed well in addressing climate change. From the comparison of various continents, the ability and level of various regions to cope with climate change are extremely uneven. Africa, with the largest concentration of developing countries, needs more support and help from other countries.

**Asia** In 2018, we found that the top three Asian countries are China, the Philippines, and Japan, and the bottom countries are Qatar, Kazakhstan, and Saudi Arabia. After the Copenhagen Conference, China changed its attitude towards climate change and began to take more active measures to address the challenges of climate change. In 2013, the Chinese government officially released the "National Climate Change Strategy." In 2015, China signed the Paris Climate Agreement and actively fulfilled its emission reduction obligations. At the General Debate of the 75th UN General Assembly in 2020, Chinese President Xi Jinping announced that China will increase its nationally determined contribution and adopt a series of more powerful measures to strive for the peak of carbon dioxide emissions by 2030 and strive to achieve carbon neutrality by 2060. Among the top three countries in Asia, China scores higher in the power generation and forest dimensions, and lower in the carbon emissions and energy consumption dimensions. The main reason why China scores high in the power generation dimension is that China is vigorously developing nuclear power, hydropower, wind power and solar photovoltaic power generation. Meanwhile, the Philippines scores high in carbon emissions and energy consumption, but it scores very low in power generation, and its performance in the forest dimension is average. The economic foundation of the Philippines is relatively weak. Although it has maintained rapid growth from 2010 to 2018, the economic structure of the Philippines is dominated by the service industry, so its industrial and manufacturing capabilities are not strong. Service-oriented economies tend to have relatively low carbon emissions and energy consumption, so the Philippines performs better in these two dimensions. Japan’s scores on carbon emissions and energy consumption are slightly lower than those of the Philippines, and its scores on the forest dimension are better However, its score in the power generation dimension has shown a clear downward trend. As mentioned above, this is mainly because nuclear power generation has been greatly affected in Japan. Japan is a major manufacturing country in Asia and the world, with strong demand for energy and electricity. Although the leak at the Fukushima nuclear power plant caused the Japanese people to strongly resist nuclear power generation, Japan currently does not have the ability to completely get rid of nuclear power generation.^5^ As a kind of clean energy, nuclear power generation is gradually recovering in Japan, which will help Japan better achieve its emission reduction targets.

In 2018, we found that the Asian countries ranked at the bottom were Qatar, Kazakhstan and Saudi Arabia. Qatar and Saudi Arabia are desert countries in the Middle East, and Kazakhstan is a landlocked country in Central Asia. These three countries are very rich in oil and natural gas resources, and exporting energy is an important pillar of their own economy. Affected by economic inertia and inherent interests, the three countries have relatively negative attitudes towards the development of new energy sources and are less active in responding to climate change. In addition, the three countries have relatively few forest resources, and as a result their capacity for carbon neutrality is comparatively insufficient. These countries should actively adapt to the requirements of the climate change era, reduce their dependence on fossil energy and actively open up new economic development paths to promote the transition of their entire societies and economies to low carbon.

**Europe** In 2018, we found that the top three European countries were Sweden, Russia, and France. Sweden and France are major economic powers in Europe, with relatively strong technological and industrial capabilities. In contrast, Russia's economy is heavily dependent on the export trade of oil and natural gas and other resources, and its level of industrialization is relatively weak. The reason why Sweden, Russia and France can rank highly is mainly because they have an outstanding performance in certain dimensions. For example, Sweden scores relatively highly in the carbon emissions, energy consumption and forest dimensions, but it scores poorly in the power generation dimension; Russia scores very highly in the forest dimension, mainly due to its vast territory and high forest coverage. Russia also scores relatively highly in the dimensions of carbon emissions and energy consumption. Although Russia is a major producer and exporter of oil and natural gas, Russia's own manufacturing industry is underdeveloped, and its own carbon emissions and energy consumption are smaller than those of some developed countries. Russia has made slow progress in the development of renewable energy, with a slightly lower score in the power generation dimension. France has a higher score in the carbon emissions and energy consumption dimensions, which is inseparable from France’s active transition to a low-carbon economy. It is a developed country and a role model in this area. However, France has a low score in the power generation and forest dimensions, especially in the use of renewable energy to generate electricity. France is not in a leading position.

In 2018, we found that the bottom three countries in Europe were Iceland, Ukraine and the Netherlands. Both Iceland and the Netherlands are developed countries. Iceland's ranking lags behind other European countries because of its poor performance in power generation, carbon emissions and energy consumption. Iceland is located in the northern part of Europe where the climate is relatively cold, and economic activities require a lot of energy consumption and produce a lot of greenhouse gas emissions. However, Iceland has a “bright spot” in power generation: its hydropower and geothermal power generation systems are relatively developed. Iceland’s hydropower performance is included in our research, but its performance in geothermal power generation is currently not included, which has somewhat dragged down Iceland’s ranking.

The Netherlands ranked 60th in climate in 2018, and its performance in the two dimensions of energy consumption and forests was poor. The Netherlands has a concentration of energy and emission-intensive industries and is heavily dependent on fossil fuels. The academic community is paying increasing attention to how the Netherlands is responding to climate change.^6^ From 2008 to 2018, the share of fossil fuels in the total primary energy supply only slightly decreased, from 92 to 90%. The Netherlands is the most economically developed country in the world, and its current performance in tackling climate change is unsatisfactory. The Dutch government is taking a series of measures to promote a cost-effective transition to a low-carbon economy. One of these measures will result in at least 70% of electricity coming from renewable energy sources (mainly wind energy and photovoltaic power generation). In addition, the Netherlands has a forest coverage rate of 11%, and the per capita forest area is low (only 0.02 hectares/person), so in the future it is also necessary to improve its performance in the forest dimension. Ukraine is a large agricultural country and has always been known as the "granary of Europe." Because agricultural activities are greatly affected by climate change, the issue of global warming has gradually attracted the attention of Ukrainians. However, Ukraine’s economic activities rely heavily on fossil energy and its energy efficiency is low, which has caused Ukraine’s poor performance in carbon emissions and power generation. Ukraine is very interested in the use of renewable energy, especially in the application of power generation, and is actively promoting the development of photovoltaic power generation projects.

**North America** In 2018, Canada's ranking was better than that of the United States. This was mainly due to the Canadian government's long-term continued attention to climate change issues. Canada’s only shortcoming is in power generation, especially in the use of renewable energy for power generation. Although the United States is the most economically developed country in the world and has the strongest scientific and technological strength, it is not the most outstanding in addressing the climate change issue. During the Obama administration, the United States not only signed the Paris Climate Agreement, but also actively promoted the development and use of renewable energy. However, the U.S. Republican Party, especially the Trump administration, is skeptical of climate change and has a negative attitude towards climate change.^7^

**Latin America** In 2018, we found that the top countries in Latin America were Brazil and Peru, and the bottom countries were Argentina and Trinidad and Tobago. Brazil ranks first not only in Latin America, but also in the entire group of 75 countries, as discussed above. It is worth noting that Brazil's virgin rainforest is decreasing. Studies have found that compared with the Democratic Congo and Indonesia, which also have tropical rainforests, Brazil’s virgin forest has been reduced by twice that of Indonesia and five times that of the Democratic Republic of Congo.^8^The reason why Peru ranks highly is that Peru performs very well in the three dimensions of energy consumption, carbon emissions and forests. Argentina is one of the more economically developed countries in Latin America, with relatively developed industry and agriculture and relatively large carbon emissions. Argentina's support for renewable energy is relatively small, and the performance in renewable energy power generation is poor. Trinidad and Tobago is an island country whose economy is dominated by energy development and processing industries, and its ability to cope with climate change is insufficient, so it ranks at the bottom among Latin American countries.

**Africa** Among the 75 selected countries, only Morocco, Egypt, Algeria and South Africa are in Africa, and their rankings are relatively low. Compared with countries on other continents, the performance of African countries in dealing with climate change is relatively poor and they are more vulnerable to the negative effects of climate change.^9^ The reason is that although the carbon emissions of African countries are generally small, their energy use efficiency is generally low, and their performance in the two dimensions of power generation and forests is also relatively poor. Morocco ranks highly in Africa because it scores highly in the dimensions of carbon emissions and energy consumption, which does not mean that Morocco’s economic activities are very focused on improving energy efficiency. The main reason is that Morocco’s economic pillars are tourism and fisheries, and industry and manufacturing are underdeveloped. South Africa ranks behind because it is the largest economy in Africa, with relatively developed industrial and manufacturing sectors, and relatively large carbon emissions.

**Oceania** In 2018, New Zealand and Australia in Oceania ranked 26th and 47th, respectively, with New Zealand performing much better than Australia. Both New Zealand and Australia performed relatively well in terms of carbon emissions and energy consumption, but their performance in the two dimensions of forests and power generation was not satisfactory. In particular, Australia has been performing poorly in forest resource protection. The annual wildfires burn down and devour a large amount of forest, resulting in a negative rate of change in Australia's forest cover. Global warming continues to pose a threat to Australia's forests, and more forest resources may be destroyed in the future. Australia needs to show greater determination to work with the rest of the world to cope with the challenges brought about by climate change.

#### Conclusion

The need to deal with the challenges brought about by climate change is urgent. Countries around the world should strengthen cooperation and strive to achieve the long-term goal set in the Paris Agreement, that is, to control the global average temperature rise to within 2 °C compared with the pre-industrial period, and efforts should be made to limit the temperature rise to within 1.5 °C. To achieve this goal, more and more countries have adopted various measures to increase their nationally determined contributions. For example, China has made a clear commitment to achieve a peak in carbon emissions by 2030 and carbon neutrality by 2060. Our research has found that countries around the world currently differ greatly in their capacity to deal with climate change issues. This difference is also reflected in their nationally determined contributions.^10^Some countries, as represented by China, have the ability to increase their national independent contributions, but most developing countries urgently need support and assistance from developed countries in terms of capital, technology and science and technology. In particular, African countries need the support of developed countries.

There is also a certain degree of differentiation within developed countries. Some countries have taken a leading position in the development of renewable energy, while the pace of some developed countries has been relatively slow. The Biden administration announced its return to the "Paris Agreement" as soon as it took office. This is a very positive signal for the world's response to climate change. With the support of world powers such as China and the United States, we can expect the world to cooperate closely on the issue of climate change.

### Issue 2: Peacekeeping

#### Introduction

War and peace have always accompanied the development of human civilization. Once a war breaks out, justice disappears. Although there has been no major global war since the end of World War II, local conflicts have not ceased and have intensified in some places. Regional conflicts surrounding resources, borders, ethnic conflicts, historical disputes and other factors have always been important factors threatening regional security and development. There can be no justice without security. Therefore, resolving regional conflicts is a long-term focus of the international community, as well as a difficulty and challenge. After World War II, the United Nations came into being. Maintaining peace, preventing and resolving conflicts and wars are important goals of the United Nations. Since the mid-twentieth century, peacekeeping operations have become an important task of the international community, and especially of the United Nations. More and more countries are sending military personnel to participate in UN peacekeeping operations. Some countries provide large amounts of financial support to maintain the smooth progress of UN peacekeeping operations. After the end of the Cold War, United Nations peacekeeping operations have become an important means of regional conflict management and resolution and have received extensive attention and support globally.

The content and scope of peacekeeping work has undergone great changes from the mid-twentieth century to today.^11^Our understanding of peacekeeping is constantly deepening. The traditional understanding is that peacekeeping work is mainly related to regional security issues, but now peacekeeping work also involves human rights protection, the establishment of the rule of law- and the organization of elections. While UN peacekeeping operations have produced good results, we also find that the willingness and ability of countries to participate in UN peacekeeping is undergoing great changes. In the early days of the rise of peacekeeping, Western developed countries were the most important participating countries. In the following decades, developing countries continued to join the peacekeeping work and gradually grew into the backbone of the UN peacekeeping work. Although developed countries are still actively participating in UN peacekeeping operations, the number of personnel dispatched shows a clear downward trend. Our research analyzes the contributions of countries around the world to UN peacekeeping operations over the past few years. This is conducive to analyzing the changing trends in UN peacekeeping operations and provides a reference for the future reform and development of UN peacekeeping.

#### Dimensions and Indicators

In general, there are two types of peacekeeping missions. The first type of peacekeeping mission is conducted by the United Nations (UN) and the second is conducted by various regional organizations. Both types of peacekeeping activity are important, but scholars and policy analysts have different opinions on each of them.^12^ In general, more people support peacekeeping operations conducted by the United Nations. The reason is obvious. The United Nations enjoys compelling, overwhelming and incomparable authority in peacekeeping. The peacekeeping efforts of regional organization enjoy certain advantages, but they cannot be comparable to the UN peacekeeping in terms of authority and capacity. In addition, UN peacekeeping data are available online with detailed information about countries’ contributions in various ways. Regional peacekeeping data are not fully open source data (Table 3).

**Table 3:** Data on peacekeeping

Category	Dimension	Indicator	Data source	Coverage
Contribution	Personnel Contribution	Troops and Police	UN Peacekeeping Website International Peace Institute	129 (2010–2018)
	Financial Contribution	Donation		120 (2010–2018)

As such, our empirical analysis of countries’ contributions to peacekeeping is limited to UN peacekeeping contributions. It includes two dimensions: personnel contribution and financial contribution. Personnel contribution is measured by the troops and police indicator, while the financial contribution is measured by the donation indicator. These data are all available on the UN peacekeeping website and international Peace Institute. Our time span is from 2010 to 2018. In the future, we may consider including regional peacekeeping contributions to our analysis.

#### Results

In this section, we present the ranking results of countries’ contributions to global justice from a peacekeeping perspective (Table 4). Table 4 shows nine years of results from 2010 to 2018 in 192 countries.

**Table 4:** Country ranking in the peacekeeping aspect of promoting global justice

Country	2010	2011	2012	2013	2014	2015	2016	2017	2018
United States of America	1	1	1	1	1	1	1	1	1
Ethiopia	19	15	5	5	5	3	2	2	2
Bangladesh	3	2	2	2	2	2	5	4	3
Rwanda	13	13	8	7	6	6	7	7	4
India	4	4	4	4	3	5	3	3	5
China	11	12	7	9	8	7	6	6	6
Pakistan	2	3	3	3	4	4	4	5	7
Nepal	9	9	11	8	7	8	8	8	8
Egypt	6	7	10	14	15	20	14	10	9
Japan	7	6	9	10	10	9	10	11	10
France	8	8	12	12	11	10	13	12	11
Germany	16	16	16	17	17	16	19	15	12
Indonesia	23	23	23	23	26	15	15	13	13
Ghana	14	17	14	13	12	12	11	14	14
United Republic of Tanzania	32	27	26	21	19	22	18	18	15
Senegal	20	21	19	16	13	11	9	9	16
United Kingdom of Great Britain and Northern Ireland	15	14	17	18	18	17	20	17	17
Italy	10	11	15	15	14	14	17	19	18
Burkina Faso	34	30	32	27	22	19	12	16	19
Morocco	24	24	24	24	21	21	22	20	20
Chad	89	89	88	40	33	34	26	23	21
Togo	38	41	40	28	27	25	23	24	22
Spain	18	18	22	25	28	27	28	22	23
Republic of Korea	25	25	25	29	30	31	31	27	24
Russian Federation	28	32	29	32	34	35	30	26	25
South Africa	22	22	21	20	20	23	24	25	26
Cameroon	76	76	72	80	29	18	32	31	27
Zambia	37	45	63	70	75	50	39	32	28
Mauritania	179	179	140	138	93	74	40	33	29
Niger	45	38	30	26	25	24	21	29	30
Canada	26	26	28	33	79	86	36	35	31
Uruguay	17	20	20	19	24	29	27	28	32
Malawi	68	42	35	41	40	40	37	37	33
Guinea	86	87	90	73	67	46	42	38	34
Mongolia	50	80	45	35	36	39	38	34	35
Malaysia	30	29	27	34	37	41	43	40	36
Jordan	12	10	13	11	16	26	33	39	37
Benin	27	33	34	30	31	30	29	36	38
Cambodia	80	61	57	52	43	42	44	42	39
Burundi	88	79	77	69	47	32	35	41	40
Netherlands	39	40	42	46	35	33	41	43	41
Australia	36	35	39	39	42	44	46	44	42
Sri Lanka	29	28	31	31	32	43	51	49	43
Fiji	60	59	58	48	41	45	47	45	44
Ireland	52	48	43	45	49	52	52	46	45
Sweden	46	49	52	54	53	47	48	47	46
Uganda	66	88	94	93	96	100	49	48	47
Nigeria	5	5	6	6	9	13	16	21	48
Brazil	21	19	18	22	23	28	25	30	49
Finland	59	62	59	51	46	51	54	54	50
Gabon	145	145	146	145	69	53	56	52	51
Argentina	33	31	33	36	38	48	55	50	52
Austria	40	39	38	42	50	54	57	55	53
Switzerland	47	50	54	55	55	57	60	56	54
Belgium	41	44	47	49	52	59	63	63	55
Ukraine	53	56	46	43	44	49	50	53	56
Norway	49	55	56	57	56	56	59	58	57
Saudi Arabia	64	65	68	68	74	73	66	61	58
Portugal	44	47	53	71	73	71	77	59	59
Greece	56	58	61	59	64	65	72	71	60
Serbia	94	91	82	72	66	60	62	60	61
Cote d'Ivoire	69	68	69	60	62	76	124	75	62
Gambia	51	52	50	53	54	61	61	57	63
Peru	54	54	49	50	51	62	58	62	64
Slovakia	62	64	66	64	68	67	71	69	65
Turkey	42	46	41	44	60	66	70	72	66
Tunisia	58	95	92	67	76	69	65	65	67
El Salvador	79	82	83	79	83	72	69	68	68
Denmark	48	51	62	62	63	63	67	64	69
Kenya	35	36	37	37	39	36	34	66	70
Djibouti	98	96	95	74	72	75	75	74	71
Guatemala	57	57	60	58	59	64	68	70	72
United Arab Emirates	71	70	67	66	71	70	74	73	73
Congo	149	160	156	159	58	38	45	51	74
Israel	67	69	71	76	78	78	78	76	75
Singapore	70	71	73	78	82	84	79	77	76
Mexico	65	67	75	77	81	81	83	80	77
Czechia	90	84	76	81	84	85	82	82	78
Zimbabwe	75	75	85	82	89	92	85	85	79
New Zealand	74	74	79	85	87	90	86	87	80
Liberia	179	179	163	103	97	97	95	91	81
Sierra Leone	61	53	51	65	80	89	88	84	82
Hungary	73	73	74	75	77	79	81	81	83
Poland	77	78	78	84	85	87	87	88	84
Kuwait	84	83	84	88	91	93	89	89	85
Romania	78	81	80	83	86	82	80	83	86
Chile	43	43	44	47	48	55	53	67	87
Qatar	93	93	87	90	94	94	91	90	88
Namibia	87	86	89	94	88	91	92	96	89
Mali	81	77	81	87	90	88	90	93	90
Croatia	72	72	70	91	104	104	108	108	91
Estonia	111	108	108	108	110	98	94	92	92
Thailand	82	37	48	96	99	80	97	99	93
Slovenia	92	90	91	95	98	96	98	97	94
Bhutan	191	191	192	185	150	112	103	100	95
Bosnia and Herzegovina	101	100	96	92	95	99	99	98	96
Brunei Darussalam	105	97	99	99	105	102	102	101	97
Lithuania	119	116	119	117	116	121	120	116	98
Armenia	155	153	142	144	125	103	101	103	99
Venezuela (Bolivarian Republic of)	102	104	97	98	103	101	104	104	100
Paraguay	83	66	65	63	70	77	76	78	101
Iran (Islamic Republic of)	106	106	103	104	109	110	105	106	102
Oman	114	112	100	100	106	106	106	105	103
Kazakhstan	118	120	116	121	124	115	111	112	104
Colombia	95	99	98	97	101	107	96	95	105
Philippines	31	34	36	38	45	68	73	86	106
Bolivia (Plurinational State of)	55	63	64	61	65	83	107	94	107
Kyrgyzstan	104	107	105	101	107	111	110	109	108
Madagascar	91	92	93	89	102	108	100	107	109
Luxembourg	96	98	102	102	108	109	109	110	110
Samoa	112	117	110	112	112	116	113	111	111
Viet Nam	133	133	133	133	136	127	121	121	112
Cyprus	107	105	106	107	111	113	112	113	113
Latvia	126	126	127	126	132	130	126	123	114
Honduras	115	113	112	113	100	95	93	102	115
Ecuador	85	85	86	86	92	105	114	119	116
Bahrain	113	111	113	114	118	119	116	118	117
Algeria	116	110	111	110	114	118	115	114	118
Libya	99	101	109	111	115	114	117	120	119
Republic of Moldova	121	118	118	115	119	120	118	124	120
Cuba	125	124	128	127	133	131	132	117	121
Belarus	129	125	120	116	122	124	122	122	122
Bulgaria	124	122	121	122	126	126	127	126	123
Iraq	141	139	129	128	134	132	123	127	124
Dominican Republic	129	127	132	130	131	136	134	115	125
Iceland	108	109	115	118	121	123	125	128	126
Montenegro	128	123	122	123	127	133	129	129	127
Costa Rica	132	132	138	137	142	141	139	130	128
Solomon Islands	179	179	183	185	186	186	143	132	129
Malta	122	119	124	124	129	128	128	131	130
Yemen	63	60	55	56	61	58	64	79	131
Trinidad and Tobago	120	115	117	119	123	125	131	134	132
Papua New Guinea	165	156	145	140	151	144	129	137	133
Bahamas	123	121	125	125	130	129	132	135	134
Azerbaijan	146	142	135	135	140	138	136	136	135
Democratic Republic of the Congo	109	102	104	109	57	37	84	125	136
Central African Republic	100	94	101	106	117	135	186	133	137
Monaco	146	142	130	129	135	134	138	138	138
Lebanon	133	133	133	133	138	137	140	139	139
Myanmar	161	160	156	159	161	147	135	143	140
Panama	140	138	141	142	147	148	141	141	141
Andorra	131	131	135	135	140	138	142	142	142
Turkmenistan	136	135	147	146	152	150	145	144	143
Republic of North Macedonia	142	140	143	143	149	149	148	147	144
Syrian Arab Republic	138	136	139	139	145	143	146	145	145
Uzbekistan	152	150	149	149	155	154	147	146	146
Tajikistan	117	114	114	105	120	164	162	163	147
Cabo Verde	168	168	171	173	170	168	175	156	148
Barbados	139	137	148	148	154	153	152	150	149
San Marino	146	142	149	149	155	154	151	149	150
Botswana	143	141	123	147	153	151	152	150	151
Mauritius	150	149	151	152	158	157	154	153	152
Jamaica	103	103	107	120	113	117	144	152	153
Albania	97	130	153	155	143	142	156	154	154
Georgia	154	152	154	157	159	156	137	140	154
Suriname	161	160	161	164	165	162	157	155	156
Angola	155	153	156	159	161	159	158	158	157
Democratic People's Republic of Korea	153	151	155	158	160	158	158	158	157
Eritrea	179	179	183	185	186	186	186	186	157
Timor-Leste	179	165	126	141	137	140	155	162	157
Vanuatu	110	179	183	151	128	122	119	167	157
Nicaragua	161	160	164	166	167	164	162	163	162
Afghanistan	165	166	166	168	169	167	165	165	163
Antigua and Barbuda	158	157	161	164	165	162	165	165	163
Eswatini	161	160	164	166	167	164	168	168	165
Guyana	168	168	171	173	175	174	168	168	165
Maldives	179	179	171	173	175	174	168	168	165
Kiribati	179	179	183	185	186	186	186	186	168
Lesotho	151	148	131	131	139	146	164	186	168
Saint Kitts and Nevis	168	168	168	170	170	168	171	171	168
Sudan	155	153	156	159	161	159	158	158	168
Belize	168	168	171	173	175	174	175	175	172
Dominica	168	168	171	173	175	174	175	175	172
Grenada	135	128	152	156	146	145	167	175	172
Guinea-Bissau	127	129	183	154	148	152	149	148	172
Marshall Islands	168	168	171	173	175	174	175	175	172
Micronesia (Federated States of)	168	168	171	173	175	174	175	175	172
Mozambique	137	147	144	153	157	171	150	157	172
Nauru	168	168	171	173	175	174	175	175	172
Palau	144	146	137	132	144	174	175	175	172
Saint Lucia	168	168	171	173	175	174	175	175	172
Saint Vincent and the Grenadines	168	168	171	173	175	174	175	175	172
Sao Tome and Principe	179	179	183	185	186	186	186	186	172
Seychelles	158	157	168	170	173	171	175	175	172
Tonga	168	168	171	173	175	174	175	175	172
Equatorial Guinea	158	157	156	159	161	159	158	158	186
Haiti	167	167	168	170	173	171	171	171	186
Lao People's Democratic Republic	179	179	171	173	175	174	171	171	186
Somalia	179	179	183	185	186	186	186	186	186
South Sudan	191	191	167	169	170	168	171	171	186
Tuvalu	179	179	183	185	186	186	186	186	186
Comoros	179	179	183	185	186	186	186	186	192

The above table shows that from 2010 to 2018, countries such as the United States, Ethiopia, Bangladesh, Rwanda, India, China, Pakistan, Nepal, Japan*, and France have consistently performed well in peacekeeping. Among them, Ethiopia, Bangladesh, Rwanda, India, China, Pakistan and Nepal are all developing countries, while the United States, Japan and France are developed countries. Among them, the United States, China and France are permanent members of the Security Council. Judging from the performance of the top ten, developing countries perform better than developed countries in peacekeeping. This result shows that the degree of economic development itself may not be the main factor influencing the country’s participation in UN peacekeeping. Among the top ten countries, six countries are in Asiad, one country is in North America, one country is in Europe, no country is in Latin America, two countries are in Africa, and no country is in Oceania. From the perspective of geographical distribution, Asian countries have contributed more to peacekeeping than other continents.

The United States has long been ranked No. 1 for its contribution to peacekeeping, and it is a model for developed countries’ contribution to UN peacekeeping. The United States is a permanent member of the United Nations Security Council and the most powerful country in the world. It has also played the role of "world police" for a long time. The number of peacekeepers (military and police) sent by the United States used to be large, but the number of peacekeepers has gradually decreased over the past few years. At the same time, the United States has increased its financial support for UN peacekeeping. Although the number of peacekeepers dispatched by the United States is decreasing, financial support for peacekeeping has been increasing, which has resulted in the United States ranking first in peacekeeping. In fact, not only is the United States gradually reducing the number of peacekeepers it dispatches, but other developed countries are also reducing the number of peacekeepers they dispatch. The reason why developed countries choose to reduce personnel dispatch and increase financial support is likely to be related to the fact that domestic public opinion is very concerned about casualties among peacekeepers.^13^

Ethiopia is a country with relatively large economic strength, land area and population in Africa. In the past 9 years, Ethiopia’s contribution to UN peacekeeping has jumped to second place in the world. The main reason behind this is that Ethiopia is greatly increasing the number of peacekeeping personnel. Taking 2018 as an example, Ethiopia was the country that sent the most peacekeepers. The situation in Bangladesh, India, Pakistan and China is similar to that of Ethiopia. They have all gradually increased their numbers of peacekeeping personnel. This also shows the strong willingness of these countries to support UN peacekeeping. France is a European country that has long performed well in peacekeeping contributions. On the one hand, France is a permanent member of the UN Security Council, and it has assumed the responsibility of maintaining world peace. On the other hand, France has historically been inextricably linked with African countries, especially with its former colonies. France itself, therefore, has a strong need to ensure stability of the situation in Africa, and because many United Nations peacekeeping operations take place in Africa, naturally France will attach great importance to it.

India's contribution to UN peacekeeping has long ranked among the top 5 in the world. India was one of the earliest countries in the world to participate in UN peacekeeping, and it has always been very active. Some statistics show that India has sent more than 180,000 people to UN peacekeeping operations, making it one of the countries with the largest number of personnel sent to peacekeeping operations. India has also displayed its own characteristics and innovations in participating in UN peacekeeping. For example, in 2007, India became the first country to send an all-female peacekeeping team to a UN peacekeeping operation. Because India has been involved in UN peacekeeping for a long time and has participated in more peacekeeping operations, and because some peacekeeping operations are very dangerous, there has been a relatively large number of casualties in India’s peacekeeping operations. Statistics show that more than 160 Indian peacekeepers have died in peacekeeping operations.^14^

China used to reject participation in UN peacekeeping operations, but now it has not only become an active participant, but also has made major contributions to participating in UN peacekeeping operations.^15^ In 2012, China became a top ten country for the first time, and further rose to sixth place in 2018. Compared with many developed countries, China's participation in UN peacekeeping operations started relatively late, but the pace of development has been fast. In April 1990, the Chinese army sent five military observers to the UN Truce Supervision Organization. This was the beginning of China's participation in UN peacekeeping operations. As of 2020, China's peacekeeping operations will cover more than 20 countries and regions including Cambodia, Liberia, Congo (Kinshasa), Cyprus, Sudan, Lebanon, South Sudan, Mali and Central Africa. According to the data in the white paper "The Chinese Army's Participation in UN Peacekeeping Operations for 30 Years", the Chinese military has participated in 25 UN peacekeeping operations. As a key force in UN peacekeeping operations, China’s role includes six aspects: monitoring ceasefires, stabilizing the situation, protecting civilians, acting as security guards, supporting guarantees and spreading hope. Upholding the concept of "a community with a shared future for mankind", we can expect China to play a greater role in maintaining peace.

The above table shows that from 2010 to 2018, countries such as Laos, Somalia, South Sudan, Tuvalu and Comoros ranked last in terms of peacekeeping performance. These five countries have relatively small territories, relatively small populations, and relatively low levels of economic development. They are typical small countries. In countries such as Somalia and South Sudan, their domestic social order is relatively unstable, with internal ethnic, social and criminal problems emerging one after another. As their domestic problems are still very serious, it is difficult for these countries to make greater contributions to the UN peacekeeping cause. UN peacekeeping work requires financial and personnel technical support. Many small countries often do not have these capabilities and, therefore, cannot make a substantial contribution to UN peacekeeping.

From the data, we can find that the rankings of most countries are relatively stable, but there are also some countries whose rankings have experienced greater fluctuations from 2010 to 2018. For example, Italy's ranking has been gradually declining. Italy is a large European country, which formerly had colonized some countries in Africa, and is thus inextricably linked with Africa. And because Italy and the northern African countries belong to the countries along the Mediterranean Sea, in fact they are "across the sea" from each other, and as such Italy is affected by the impact of African refugees. However, in recent years, with the weakening of the Italian economy and the rise of a populist government in Italy, Italy's willingness and ability to participate in international affairs has gradually declined. During the same period, the rankings of the Philippines, Yemen, Albania, Nigeria and other countries dropped significantly. The reasons for the decline in the ranking of these countries are different. For example, Nigeria’s ranking dropped from 5th in 2010 to 48th in 2018. The reason for the decline in ranking is that Nigeria’s peacekeeping focus has always been on parts of West Africa. As the security situation in this region continues to improve, Nigeria has gradually reduced its peacekeeping forces.^16^The ranking of the Philippines has gradually dropped from 31st in 2010 to 106th in 2018. The purpose of sending peacekeepers by the Philippines is to advance national interests and better participate in international military cooperation. Satisfying the needs of national interests is the primary reason. The Philippines tends to send peacekeepers to areas where there are more Filipinos overseas in accordance with the needs of the United Nations. This is because a large number of Filipino workers working overseas are sending money to their home country as an important source of income for the Philippines. Instability in some areas will affect the work of Filipino workers in these places and directly affect their remittances to their home country.^17^

From 2010 to 2018, we found that Chad, Mauritania, Guyana, Cambodia, Gabon, Congo, Liberia and other countries have greatly increased their rankings. For example, Gabon’s ranking in peacekeeping rose from 145th in 2010 to 51st in 2018. Congo’s ranking in peacekeeping rose from 149th in 2010 to 74th in 2018. The countries that have risen sharply in these rankings are basically African countries and especially Central and West African countries. These African countries have increased their support for United Nations peacekeeping operations in the past few years and in particular have actively participated in peacekeeping operations in some areas of Africa. Among them, the most typical is the participation of countries such as Chad, Gabon, and Congo in the UN peacekeeping operations in the Central African Republic. The Central African Republic has been in a state of civil war since 2012. The government forces and the rebel coalition "Séléka" have been in a state of ongoing conflict.^18^ Because the conflict in Central Africa still shows no sign of ending, these African countries may continue to be at the forefront of the ranking.

#### Regional Analysis

**Fig. 3:**
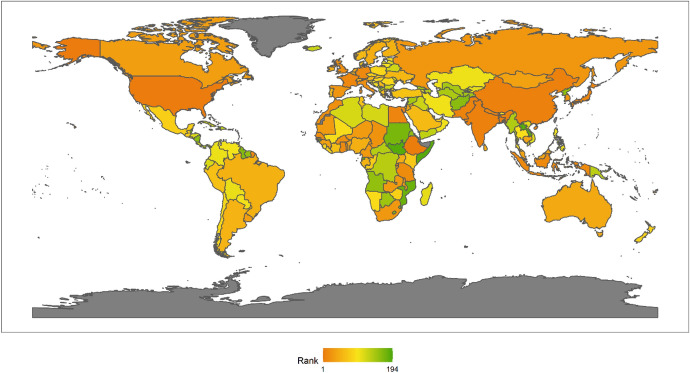
2018 Index ranking of peacekeeping on a world map

In 2018, the top 10 countries in the field of peacekeeping were the following: the United States, Ethiopia, Bangladesh, Rwanda, India, China, Pakistan, Nepal, Egypt and Japan (Fig. 3). Among the top 10 countries, there is 1 American country, plus 6 Asian countries, and 3 African countries. No European country made the top 10. There are eight developing countries and two developed countries. This shows that the degree of economic development is not necessarily related to participation in UN peacekeeping. Both developed and developing countries have the opportunity and ability to play an important role in participating in UN peacekeeping. In fact, from the perspective of the top 10 selected countries, the contribution of developing countries to the cause of UN peacekeeping may be greater than that of developed countries.

The above-mentioned countries are able to rank highly in the field of peacekeeping because they have some common characteristics. First, these countries are generally concerned about the international and regional security situation. Needless to say, the United States has long played the role of “world police”. China and Japan are also very concerned about the international and regional security situation. China has always cherished a peaceful international and regional environment, because China's development cannot be separated from world peace. Second, the military capabilities of these countries are generally relatively strong. United Nations peacekeeping contributions are mainly personnel contributions and financial contributions. At present, personnel contributions are the core part. Sending troops and police to UN peacekeeping operations has certain requirements for the military capabilities of the sending country. United Nations peacekeeping operations are highly dangerous and peacekeepers are likely to be involved in local armed conflicts. United Nations peacekeepers sent by countries with stronger military capabilities can better deal with complex local political and military risks. Third, these countries aspire to play a more important role in international and regional affairs. The United States, China, India and Japan are all major countries with global influence, and have been very active in global governance. The United States and China are permanent members of the UN Security Council, and it is their responsibility to actively participate in peacekeeping operations. India and Japan have always sought to be among the permanent members of the UN Security Council, so they are very enthusiastic about participating in UN peacekeeping.

**Fig. 4:**
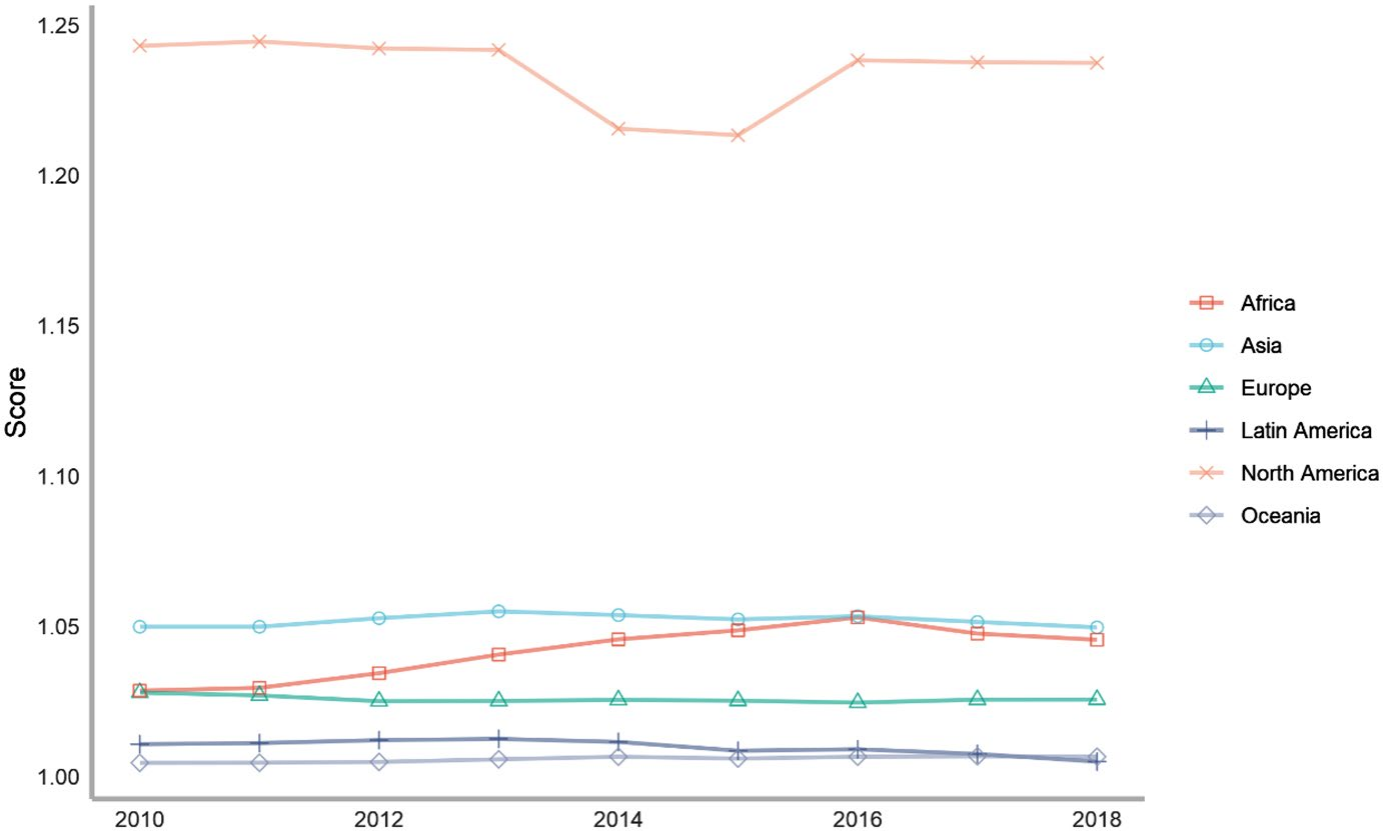
The score of peacekeeping across continents, 2010–2018

Next, we also classify countries according to their continents. These continents include Asia, Europe, North America, Latin America, Africa and Oceania. The ranking of each continent is obtained by calculating the average of the rankings of these countries. We drew a line chart to achieve a visual presentation to compare the differences in the contribution of each continent in peacekeeping (Fig. 4).

From the above figure, we can find that taking 2018 as an example, in terms of participating in UN peacekeeping, if we compare the average performance of countries on each continent, North American countries have contributed the most, followed by Asian and African countries and finally Latin American and Oceanian countries. North American countries only include the United States and Canada, which have performed very well in peacekeeping. African countries as a whole have also made relatively large contributions to peacekeeping. This is partly because many peacekeeping operations have taken place on African territory. Compared with other continents, Latin America and Oceania are at the bottom of the ranking. Latin American countries rank lower mainly because some island countries in Central America tend to have lower rankings; while Oceanian countries rank at the bottom because both the large countries in the region (Australia and New Zealand) and small Oceanian countries generally lag behind in their peacekeeping contribution rankings.

**Asia** In 2018, we found that the top 5 Asian countries are Bangladesh, India, China, Pakistan and Nepal, and the bottom countries are North Korea, Afghanistan, the Maldives, Micronesia and Laos. China and India are the top countries in the Asian region in terms of comprehensive national strength and are the two most populous countries among all developing countries. India was one of the earliest countries to participate in peacekeeping among all Asian countries, and it has consistently maintained this relatively high enthusiasm for participation. It is unexpected that Bangladesh ranks No. 1 in Asia. Bangladesh has a population of over 100 million, but its level of economic and social development is low, and nearly half of the population lives below the poverty line. Bangladesh has participated in UN peacekeeping work since the 1980s and has become the largest source of UN peacekeeping forces in this century.^19^There are many reasons why Bangladesh actively participates in UN peacekeeping. On the one hand, this can greatly improve Bangladesh's regional and global reputation. On the other hand, Bangladesh can also receive a large amount of financial compensation. These funds can be used in all aspects of the country's construction in addition to paying for the soldiers killed and wounded in peacekeeping missions. Obtaining some financial assistance through participating in UN peacekeeping is a very important reason for developing countries to participate in peacekeeping.^20^ Pakistan ranks highly in the field of peacekeeping, and even ranked second in the world in 2010. Pakistan and India are hostile to each other, and the foreign policies of the two countries are highly targeted at each other. Because India is actively participating in global peacekeeping operations to expand its influence, Pakistan also has the motivation to strengthen its influence in UN peacekeeping operations.^21^Similar to Bangladesh, Pakistan's participation in UN peacekeeping operations is also motivated by financial returns. In the peacekeeping rankings of Asian countries, the countries that lag behind are often small and relatively closed countries. North Korea is a closed country and has not been involved in global and regional security affairs for a long time. Therefore, it is reasonable for it not to actively participate in the UN peacekeeping work. The Maldives, Micronesia and Laos are all small Asian countries, and their own capabilities limit their participation in UN peacekeeping operations.

**Europe** In 2018, we found that the top five European countries are France, Germany, the United Kingdom, Italy and Spain, and the bottom countries are Morocco, Andorra, North Macedonia and Albania. France and the United Kingdom are permanent members of the UN Security Council, and they themselves shoulder responsibility for maintaining global and regional security. Active participation in UN peacekeeping is a long-term foreign policy of France and the United Kingdom. Germany caused tremendous damage to global security in the Second World War. After the end of World War II, Germany adopted a diplomatic and security strategy of "hiding our power and biding our time" and did not actively participate in the work of the United Nations. It was not until the 1990s that Germany began to gradually participate in UN peacekeeping operations. Germany is a country with a large number of military and police contributions among developed countries, which notable as many developed countries are now gradually reducing the number of people they discharge. Germany is the fourth largest United Nations’ dues-paying country and has made a great financial contribution to UN peacekeeping.^22^Another important reason why Germany has strengthened its contribution to UN peacekeeping is that it hopes to become a permanent member of the Security Council. At this point, the situation in Germany is similar to that of Japan and India. Britain once ranked higher than Germany in terms of peacekeeping, but in recent years Britain has begun to implement an "isolationist" policy. Britain’s contribution to peacekeeping is on a downward trend, which is basically consistent with Britain’s departure from the European Union. Morocco, Andorra, North Macedonia and Albania, which rank at the bottom, are all small European countries with a small population and a relatively small land area. They have long pursued a policy of partial security and are not actively participating in global governance.

**North America** The United States is a permanent member of the UN Security Council. It has long regarded itself as a "world policeman", and its active participation in UN peacekeeping is in line with this. On the other hand, the United States has a unique practical advantage in participating in UN peacekeeping. A large number of US defense funds are used to maintain various military bases outside the United States. According to incomplete statistics, the United States has more than 800 military bases in approximately 70 countries and regions. The existence of these military bases helps the United States keep abreast of the security situation and the latest trends in various countries and regions around the world.^23^ These resources are a huge supporting force for the United States’ participation in UN peacekeeping. In recent years, the United States’ financial support for UN peacekeeping has been continuously increasing, while its contribution to UN personnel has gradually weakened. This is consistent with the performance of many other developed countries in UN peacekeeping. Canada is a developed country with a large area and a sparse population. Although it has a small population, it has always been a country with a global perspective. Canada participated in UN peacekeeping operations relatively early on. In the 1960s, participation in UN peacekeeping even became a part of Canada’s national identity, a responsibility and contribution that Canadians are proud of. Although the peacekeeping “fever” gradually subsided in Canada due to scandals and other reasons, Canada is still an important participant in UN peacekeeping.

**Latin America** In 2018, we found that the top three Latin American countries were Uruguay, Brazil and Argentina. The bottom countries are Grenada, Dominica, Saint Lucia, Saint Vincent and the Grenadines and Haiti. Brazil and Argentina are the most important countries in South America, and it is not surprising that they are at the forefront of the UN peacekeeping rankings. Compared with Brazil and Argentina, Uruguay is a very small country. Uruguay has a population of just over 3 million, and its territory is the second smallest in Latin America, after Suriname. However, this relatively small country ranks much higher than Brazil and Argentina in terms of UN peacekeeping. There are many reasons why Uruguay ranks first in Latin America.

First of all, Uruguay has a relatively sound economic foundation, and its per capita GDP has long ranked first in Latin America. Second, Uruguay's peacekeeping capabilities have received assistance and support from the United States. Finally, Uruguay has a relatively long experience in participating in UN peacekeeping and has become one of the countries with the largest number of military and police contributions per capita to UN peacekeeping. The countries at the bottom are mainly small countries in Central America. These countries have small populations, low economic and social standards and severely inadequate national capabilities. Some of these countries are themselves destination countries for UN peacekeeping. A typical country in this regard is Haiti. For example, the United Nations Stabilization Mission in Haiti was established in Haiti in 2004 and only ended in 2017. The lack of national capabilities restricts these countries from playing a greater role in UN peacekeeping.

**Africa** In 2018, we found that the top five African countries are Ethiopia, Rwanda, Egypt, Ghana and Tanzania, and the bottom countries are Guinea-Bissau, Equatorial Guinea, South Sudan, Somalia and Comoros. Ethiopia is the most populous landlocked country in the world and the second most populous country in Africa. Ethiopia has participated in UN peacekeeping operations since the 1950s and has continued to do so to this day. At present, Ethiopia has become the country that has contributed the most to UN peacekeeping in terms of numbers. This is also the main reason why it can rank second in the world for its contribution to UN peacekeeping. Rwanda is a small country in Africa with a very small area. Rwanda experienced a terrible genocide in the 1990s, and is a country in which UN peacekeeping operations have also intervened. Rwanda has participated in UN peacekeeping operations since 2005. Although it started relatively late, it developed rapidly. Now Rwanda has become one of the countries that provides the most personnel support for UN peacekeeping—a very striking performance. Egypt began to participate in UN peacekeeping work in the 1960s, and today has become one of the countries with the largest number of military and police contributions. Egypt has participated in all of the UN peacekeeping projects in Africa, which shows that Egypt attaches great importance to its role in Africa’s security and stability. Ghana is a country in western Africa. Ghana began to participate in UN peacekeeping work in the 1970s, and has since increased the number of military and police dispatched, and as a result its contribution to peacekeeping has gradually increased. Tanzania is a country in eastern Africa. Tanzania has a large population, but its level of economic and social development is low. Nevertheless, Tanzania's ranking in the field of peacekeeping has been on the rise. The African countries at the bottom are Guinea-Bissau, Equatorial Guinea, South Sudan, Somalia and Comoros. These countries have relatively small populations and relatively small land areas, and their economic and social development levels are at the middle and lower levels, even for Africa. Moreover, the internal social security and stability situation of some countries is severe, and there are many domestic contradictions. These factors limit their willingness and ability to participate in UN peacekeeping operations.

**Oceania** In 2018, we found that the top three countries in Oceania are Australia, Fiji and New Zealand. The bottom countries are Tuvalu, Palau and Tonga. Australia and New Zealand are the two largest countries in Oceania, and both are developed countries. These two countries were among the first countries to participate in the UN peacekeeping work, and have made great contributions to the development of the UN peacekeeping cause. However, in recent years, the rankings of these two countries in the field of peacekeeping have shown a downward trend. The main reason is that the number of military and police personnel sent by them has declined. In contrast, Fiji's ranking in the field of peacekeeping has improved significantly. Fiji is a small country in Oceania with a population of less than one million and a very small land area. Despite being a small country, Fiji has become a model for participation during its 40-year history of participating in UN peacekeeping. By per capita standards, Fiji has been the country with the most UN peacekeepers since the 1970s. Tuvalu, Palau and Tonga are all Pacific island countries, and their own national capabilities are not enough to make a significant contribution to UN peacekeeping, so they rank very low.

#### Conclusion

World peace is a common aspiration of the whole world and this is also inherent to the topic of promoting global justice. United Nations peacekeeping operations are an important means of maintaining peace and reducing wars, and they have achieved good results over the past few decades. At the same time, great changes have taken place in the types, methods and scope of countries participating in UN peacekeeping. Although the United States is the country that has contributed the most to participating in UN peacekeeping, the number of personnel sent is far lower than that of developing countries such as Ethiopia and Bangladesh. Developing countries are increasingly emerging as the backbone of UN peacekeeping operations. From the comparison of various continents, except for the outstanding performance of North America, the overall performance of other continents is almost the same. This shows that participation in peacekeeping is not necessarily related to a country’s wealth, population and geographic location. It may be related to the country’s will, the country’s interests and the country’s security environment. Today's world is still not at peace, and various regional conflicts occur one after another. UN peacekeeping operations will not only continue, but may also expand. This requires more countries to be willing to strengthen their support for UN peacekeeping operations. This support can be carried out in the form of increased peacekeeping funds or in the form of additional peacekeepers. No matter what form it takes, this is an important contribution to world peace and global justice.

### Issue 3: Humanitarian Aid

#### Introduction

Humanitarian aid denotes the short-term assistance provided in response to natural disasters and emergencies. In recent years, international organizations are gradually building consensus about the guidelines and principles regarding humanitarian aid in both theoretical and practical respects. For example, the International Code of Medical Ethics asserts that doctors “are duty-bound to provide technically competent care, treat patients with compassion, and respect human dignity”, and the Geneva Conventions of 1949 proposed an international humanitarian law with the principles of humanity, neutrality, impartiality and independence.^24^ Providing help to save lives, reduce suffering and maintain human dignity is a vital aspect of the global justice agenda. As a result, we included this issue into our global justice index and measure each country’s efforts to provide humanitarian aid by evaluating their financial contribution to global humanitarian affairs.

#### Dimensions and Indicators

Last year, we used ten indicators to measure each country’s efforts toward humanitarian aid. These ten indicators are food, health, water, emergency response, early recovery, coordination, education, protection, agriculture and other. The last indicator, “other,” denotes donations without a designated use. This year, we added the sector of “housing” into the measurement, with the other scores unchanged; thus there are 11 indicators in all. We included humanitarian donations from each country to UN departments, nongovernmental organizations and other relevant organizations such as the World Food Program, the World Health Organization (WHO) and the International Federation of Red Cross and Red Crescent Societies. We also include the one-to-one donations from one nation state directly to another nation state. Data are obtained from the Financial Tracking Service database, managed by the UN Office for the Coordination of Humanitarian Affairs. It “aims to present a complete picture of all international humanitarian funding flows” such that it “supports the transparency and accountability of the humanitarian system and facilitates resource mobilization.”^25^ Please see below the details of all the indicators in the measurement of humanitarian aid (Table 5).

**Table 5:** Indicators of humanitarian aid

Issue Area	Indicator	Source	Coverage
Humanitarian Aid	Food	Financial Tracking Service	181 (2010–2018)
	housing		
	Health		
	Water		
	Emergency Response		
	Early Recovery		
	Coordination		
	Education		
	Protection		
	Agriculture		
	Other		

In contrast to our measurement of other issues, here we sum up the amount of donations to the 11 indicators to obtain a total number and use GDP per capita to control for the impact of economic size. The underlying principle is that countries with rich resources and big economies have stronger financial strength to provide humanitarian aid, and it is not reasonable to compare the contributions of big and small countries by the same criteria.

#### Results

According to the results, the Unites States performs excellently on the issue of humanitarian aid (Table 6). It has maintained first place from 2010 to 2018. Rich countries in Asia such as Saudi Arabia and the United Arab Emirates contributed greatly as well. European countries ranked at the forefront generally.

**Table 6:** Country ranking in humanitarian aid

Country	2010	2011	2012	2013	2014	2015	2016	2017	2018
United States of America	1	1	1	1	1	1	1	1	1
Saudi Arabia	4	12	3	7	3	6	6	4	2
United Arab Emirates	26	17	30	14	11	8	7	12	3
United Kingdom of Great Britain and Northern Ireland	3	5	2	2	2	3	3	3	4
Germany	15	9	6	4	5	4	2	2	5
Canada	12	10	8	6	9	10	8	6	6
Kuwait	60	48	43	8	12	5	11	11	7
Japan	5	8	4	3	6	7	5	5	8
Sweden	9	7	7	5	8	12	10	9	9
Pakistan	105	30	10	65	7	2	4	8	10
Norway	17	15	11	9	15	15	9	14	11
Afghanistan	22	91	75	74	100	85	92	79	12
Denmark	21	19	20	10	13	16	12	13	13
Belgium	28	23	16	12	19	17	15	15	14
Netherlands	20	21	12	15	14	11	13	20	15
Burundi	47	110	99	87	100	94	92	79	16
Italy	30	31	27	22	23	24	16	18	17
Switzerland	24	22	15	11	17	19	18	19	18
Niger	136	110	99	87	100	94	22	10	19
Australia	19	16	17	13	20	26	17	21	20
Sudan	13	18	99	87	100	94	92	79	21
France	14	24	19	21	18	21	14	16	22
Republic of Korea	42	38	33	29	29	31	29	28	23
Finland	32	26	21	17	22	25	20	26	24
China	8	6	13	23	16	20	21	7	25
Ireland	38	32	26	19	26	29	27	29	26
Spain	11	11	24	18	25	28	31	27	27
Russian Federation	23	28	18	20	24	23	26	30	28
Qatar	102	51	29	26	27	30	35	32	29
Austria	51	44	41	37	51	42	34	33	30
Brazil	27	33	14	42	33	33	43	37	31
New Zealand	52	36	40	38	45	39	39	35	32
Nigeria	41	39	99	87	37	49	62	22	33
Luxembourg	49	45	39	35	44	41	36	39	34
South Africa	79	34	49	47	52	48	46	40	35
Turkey	18	14	32	33	35	34	41	25	36
Estonia	85	64	52	43	59	47	45	41	37
Mexico	40	57	53	51	43	64	38	51	38
Poland	55	42	38	39	48	40	33	31	39
Bangladesh	33	84	57	87	100	37	81	23	40
Czechia	64	50	44	41	54	38	40	38	41
Bulgaria	93	76	59	49	77	58	51	49	42
Slovakia	90	110	64	60	86	66	50	50	43
Indonesia	37	47	36	31	40	36	58	48	44
Kazakhstan	31	37	58	46	49	35	48	56	45
Iceland	115	89	84	56	80	62	55	46	46
Belarus	67	61	46	44	66	50	47	47	47
Argentina	122	90	69	58	79	94	68	44	48
Lithuania	97	87	85	69	72	57	53	55	49
Philippines	98	35	22	30	32	75	59	17	50
Thailand	35	69	82	50	38	27	66	42	51
Portugal	87	85	66	59	64	60	67	63	52
Romania	100	70	54	53	69	52	56	60	53
Colombia	29	102	63	45	67	61	82	57	54
Malaysia	73	88	77	61	62	56	77	43	55
Malta	112	67	86	72	78	71	61	54	56
Myanmar	114	63	71	63	85	72	74	62	57
Slovenia	94	68	80	66	84	68	65	52	58
Cote d'Ivoire	136	95	37	87	42	78	92	66	59
Monaco	121	97	87	77	87	77	79	65	60
Azerbaijan	53	53	48	54	58	43	92	53	61
Costa Rica	129	110	99	87	98	94	92	79	62
Latvia	120	81	79	67	73	65	80	79	63
Algeria	36	20	99	36	93	94	49	72	64
Mongolia	117	49	99	71	91	82	92	71	65
Central African Republic	54	110	99	87	4	94	37	79	66
Chile	125	86	81	64	71	63	76	67	67
Iraq	44	27	99	24	28	51	84	79	68
Cyprus	113	80	78	78	94	84	85	73	69
Montenegro	131	79	92	81	95	94	90	79	70
Sri Lanka	107	46	28	62	55	76	92	61	71
Armenia	101	101	62	79	76	86	83	76	72
Singapore	119	99	90	80	83	70	71	68	73
Andorra	118	96	89	76	89	80	86	75	74
Bhutan	132	107	95	87	100	45	92	77	75
Guyana	58	73	96	83	74	90	88	79	76
Namibia	134	43	99	86	50	94	92	79	77
Grenada	111	110	99	87	100	94	92	79	77
Chad	50	72	99	87	100	13	92	79	77
Mali	136	110	99	87	63	94	92	79	77
Peru	83	110	91	73	97	89	87	74	77
Palau	136	110	99	87	100	94	92	79	77
Trinidad and Tobago	91	110	99	87	96	69	92	79	77
India	2	4	25	16	10	22	30	36	77
Antigua and Barbuda	124	110	99	87	100	74	92	79	77
Djibouti	126	110	94	84	100	91	89	79	77
Tuvalu	136	110	99	87	100	94	92	79	77
Israel	123	94	72	87	56	94	92	79	77
Cameroon	136	110	99	87	100	55	92	79	77
Zimbabwe	136	110	9	87	100	94	28	79	77
San Marino	127	103	97	87	99	93	92	78	77
Angola	136	40	99	87	30	94	92	79	77
Malawi	136	110	5	87	100	14	92	79	77
Bolivia (Plurinational State of)	136	110	99	87	41	94	92	79	77
Libya	136	110	99	87	100	94	92	79	77
Maldives	136	83	99	87	100	94	92	79	77
United Republic of Tanzania	136	93	42	87	100	94	92	79	77
Morocco	10	100	47	25	36	46	92	79	77
Dominican Republic	43	110	99	87	100	94	63	79	77
Guatemala	136	110	99	87	100	94	92	79	77
Belize	136	110	99	87	100	94	92	79	77
Congo	56	52	99	87	100	94	92	79	77
Ghana	34	110	70	87	100	94	92	79	77
Sierra Leone	63	110	99	87	100	94	25	79	77
El Salvador	136	110	99	87	100	94	92	79	77
Samoa	136	75	99	87	100	94	70	79	77
Mauritania	136	110	99	32	100	94	92	79	77
Papua New Guinea	136	110	99	87	100	94	57	79	77
Botswana	99	82	65	68	81	94	75	59	77
Saint Kitts and Nevis	136	110	99	87	100	67	92	79	77
Mauritius	82	110	74	87	92	53	92	79	77
Gambia	39	110	99	87	100	94	92	79	77
Burkina Faso	59	110	99	87	100	94	92	79	77
Lesotho	136	110	99	87	100	94	32	79	77
Ukraine	57	110	60	87	100	94	92	79	77
Jamaica	136	110	99	87	100	94	92	79	77
Honduras	136	110	99	87	100	94	92	79	77
Zambia	136	110	55	87	100	94	92	79	77
Saint Lucia	104	109	99	87	100	94	92	79	77
Haiti	136	110	99	87	100	94	92	79	77
Hungary	88	71	50	48	61	54	42	45	77
Democratic Republic of the Congo	16	62	34	87	57	94	19	79	77
Turkmenistan	77	110	99	87	100	94	92	79	77
Cuba	80	110	56	87	100	94	92	79	77
Lao People's Democratic Republic	136	66	99	55	100	94	92	79	77
Tonga	136	74	99	87	100	94	92	79	77
Tunisia	66	110	99	87	100	94	92	79	77
Equatorial Guinea	75	110	99	87	100	94	92	79	77
Paraguay	136	110	99	87	100	94	92	79	77
Bosnia and Herzegovina	108	110	99	87	100	94	92	64	77
Seychelles	136	110	99	87	100	94	92	79	77
Saint Vincent and the Grenadines	109	110	99	87	100	94	92	79	77
Oman	61	110	35	28	34	94	92	79	77
Tajikistan	78	54	88	87	100	94	92	79	77
Rwanda	69	110	99	87	100	94	92	79	77
Uruguay	136	110	98	85	82	92	92	79	77
Egypt	46	29	76	87	46	94	44	79	77
Micronesia (Federated States of)	136	110	99	87	100	94	92	79	77
Serbia	106	108	99	82	100	88	52	79	77
Albania	136	105	67	87	100	94	91	79	77
Eswatini	136	110	99	87	100	94	92	79	77
Republic of Moldova	96	97	93	87	100	94	92	79	77
Lebanon	133	110	99	87	100	94	92	79	77
Ethiopia	136	3	99	87	100	94	24	34	77
Madagascar	110	110	99	87	100	94	23	79	77
Kyrgyzstan	81	59	99	87	100	94	92	79	77
Jordan	136	110	99	87	100	59	92	79	77
Liberia	84	110	99	87	100	94	92	24	77
Yemen	136	110	99	87	100	94	92	79	77
Nauru	136	110	99	87	100	94	72	79	77
Dominica	136	110	99	87	100	94	69	79	77
Senegal	45	110	99	87	100	94	92	79	77
Cambodia	89	110	99	87	100	94	92	79	77
Suriname	74	110	99	87	100	94	92	79	77
Bahamas	65	110	99	87	100	94	92	79	77
Gabon	76	110	99	87	100	94	92	79	77
Panama	130	110	99	87	100	94	92	79	77
Uganda	72	110	99	87	100	94	92	79	77
Fiji	136	110	99	87	100	94	92	79	77
Georgia	92	78	99	60	88	73	64	79	77
Ecuador	136	104	99	87	60	94	60	79	77
Benin	70	110	99	87	100	94	92	79	77
Kenya	25	25	23	34	39	94	92	70	77
Viet Nam	68	55	73	70	70	79	78	69	77
Nepal	6	2	99	87	100	9	92	79	77
Togo	136	110	99	87	90	94	92	79	77
Greece	86	58	68	57	65	87	54	58	77
Barbados	136	110	99	87	100	94	92	79	77
Mozambique	116	110	45	75	100	94	92	79	77
Uzbekistan	71	60	99	87	100	94	92	79	77
Republic of North Macedonia	103	110	99	87	100	94	92	79	77
Timor-Leste	48	41	31	87	31	44	92	79	77
Bahrain	62	56	51	27	47	83	92	79	77
Croatia	95	65	61	52	68	81	73	79	77
Brunei Darussalam	128	98	83	87	75	94	92	79	77
Nicaragua	136	106	99	87	100	94	92	79	77
Venezuela (Bolivarian Republic of)	135	77	99	87	53	NA	NA	NA	NA
South Sudan	136	13	99	40	21	18	NA	NA	NA
Eritrea	136	110	NA	NA	NA	NA	NA	NA	NA
Iran (Islamic Republic of)	7	92	99	87	100	32	92	79	NA

The United States donated more than 7 billion dollars in 2018. Most of this (more than 2 billion) went to the food security sector, followed by the sector of coordination and support services (more than 274 million) and the health sector (more than 154 million). Saudi Arabia donated more than 1 billion dollars in 2018, with most of this going to the coordination and support services and food security sector. The United Arab Emirates donated more than 2 billion dollars in 2018, with most of this going to the food security sector and the health sector. The United Kingdom donated more than 1 billion dollars in 2018, with most of this going to the food security sector and the health sector. Germany donated more than 2 billion dollars in 2018, the majority of which went to the food security sector. Canada donated more than 500 million dollars in 2018, with most of this going to the food security sector and the health sector. Through the analysis of the financial flows of the top contributors in 2018, it is obvious that most of the humanitarian aid worldwide is used to ensure food provision and fight hunger.

**Fig. 5:**
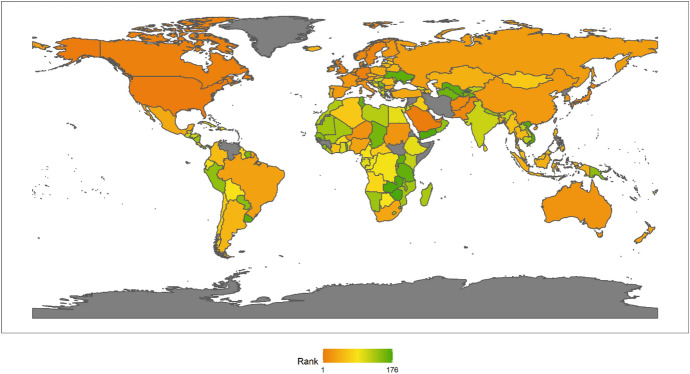
2018 Index ranking of humanitarian aid on a world map

Although there are many of organizations, programs and initiatives relating to food assistance in the world, the major way for national states to provide food aid is through the World Food Programme (WFP). The WFP is initiated by the UN and works through the UN system. It is “the leading humanitarian organization saving lives and changing lives, delivering food assistance in emergencies and working with communities to improve nutrition and build resilience.”^26^ More specifically, support from the WFP consists of the following three categories: food assistance supplied directly to families or individuals; food support supplied directly to national stakeholders (governments or civil society); and South–South and triangular cooperation, which focuses on the exchange of experience, knowledge, cash or other forms of assistance between developing countries.^27^ The latest strategic goals of the WFP are the Sustainable Development Goals set forth in the 2030 Agenda, which aims to transform lives to an unprecedented level by 2030 (Fig. 5).

The top ten countries in the provision of humanitarian aid in 2018 are the United States, Saudi Arabia, the United Arab Emirates, the United Kingdom, Germany, Canada, Kuwait, Japan and Sweden. The United States performed excellently in humanitarian aid and has consistently ranked at the top from 2010 to 2018. Arab countries with strong petroleum reserves, such as Saudi Arabia and the United Arab Emirates, contributed greatly on this issue as well. European countries ranked at the forefront generally due to their longstanding policies on humanitarian aid. Kuwait was not the largest in absolute contribution, but when its economic size is taken into account it ranks eighth.

#### Regional Analysis

See Fig. 6.

**Fig. 6:**
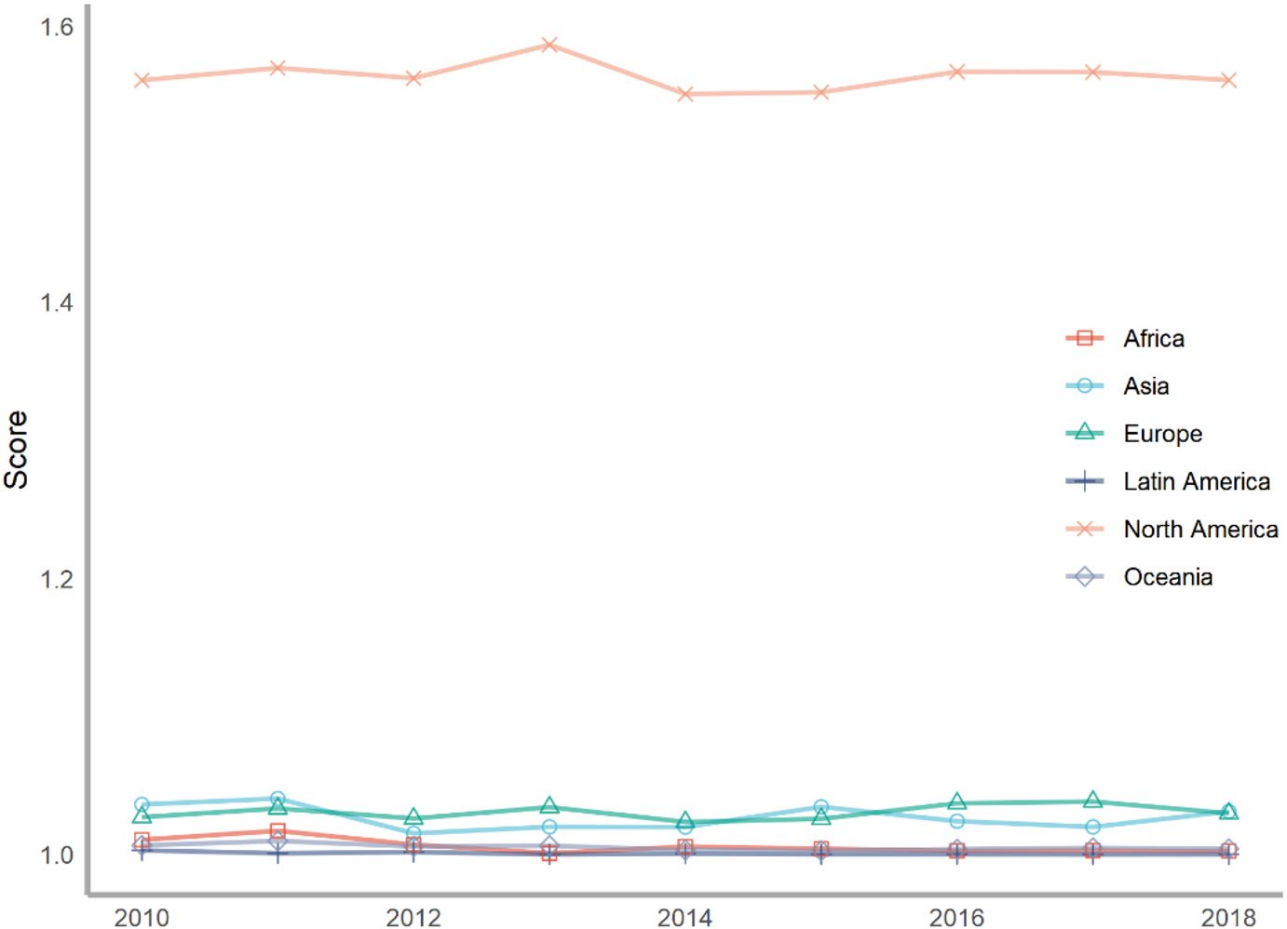
Score of humanitarian aid issue across continents, 2010–2018

**Asia** As we take into consideration the economic size of each country, it is not the states with the largest absolute donations that rank the highest, but those who contribute greatly in comparison with their GDP per capita. This explains why Pakistan, a country in South Asia, ranked tenth in 2018. According to the data, the total outgoing funding of Pakistan is about 13 million dollars. It is not a large number compared with most of the economically significant countries. For example, the Republic of Korea, which ranked 23rd in 2018, had a total outgoing funding of about 108 million dollars. However, the GDP per capita of Pakistan is 1482 dollars according to the World Bank, while that of Korea in 2018 was 33,340 dollars. When we take into the consideration their respective economic sizes and capabilities, Pakistan obtained a higher rank than Korea in 2018 with a lower absolute level of donations.

Under this measurement, Asian countries performed well on the issue of humanitarian aid in 2018. Among the top 30 countries, there were nine countries from Asia: Saudi Arabia, United Arab Emirates, Kuwait, Japan, Pakistan, Afghanistan, Republic of Korea, China and Qatar. Among these countries, there were not only rich oil states in West Asia, but also economically small countries in Central and South Asia. Countries in East Asia with close economic cooperation and high economic growth rank the forefront as well. Taking China as an example, foreign aid from China focuses generally on “large-scale infrastructure projects, energy facilities and commercial cooperation, new emphasis is being given to supporting institutional capacity building and human resource development.”^28^ Research shows that Chinese official development assistance (ODA) brings economic growth in recipient countries. For the average recipient country, one Chinese ODA project leads to a 0.7% point economic growth increase in 2 years. Through a comparison between the effectiveness of Chinese aid, the World Bank, the United States, and members of the OECD’s Development Assistance Committee, the results show that China, the United States and members of the OECD’s Development Assistance Committee have positive effects on economic growth, while there is no robust evidence showing that World Bank aid promotes growth.^29^

**Europe** Humanitarian aid has long been an important part of the EU’s agenda. Due to its long-lasting shared commitments on humanitarian assistance and its existing platform for easily transforming willingness into action, European countries perform generally well on this issue. Within the top 30 countries according to our measurement, there are 14 countries from Europe: the United Kingdom, Germany, Sweden, Norway, Denmark, Belgium, Netherlands, Italy, Switzerland, France, Finland, Ireland, Spain and Russia. Most of them are located in the Western Europe.

The EU has a long history of commitment to humanitarian assistance. Humanitarian aid first entered the EU realm through the 1969 Yaounde II Convention, and the EU’s spending on humanitarian action doubled between 1986 and 1991. In 1992, the European Community Humanitarian Office (ECHO), an administrative structure exclusively in charge of the management of humanitarian assistance, was established.^30^ In 2007, the Council of the EU, European Parliament and the European Commission signed the European Consensus on Humanitarian Aid, which provides a policy framework for the EU to provide humanitarian aid in the cases of emergencies and humanitarian crises, including natural and man-made disasters. In addition, it outlines a common vision, guidelines and principles and acts as a practical guideline. The EU reaffirms in the statement the fundamental principles of humanity, neutrality, impartiality and independence, which are fundamental to humanitarian action. Based on these fundamental principles, the EU has engaged in a series of international practical initiatives and adopted a number of relevant policies and legal acts.

**North America** From 2010 to 2018, the United States has maintained first place in humanitarian aid, and Canada has been one of the top 10 countries on this issue. The United States is the largest single provider of humanitarian assistance worldwide. As mentioned above, total outgoing funding from the United States in 2018 was more than 8 billion dollars, which included funding from the State Department’s Bureau of Population, Refugees, and Migration and the U.S. Agency for International Development’s Bureau for Democracy, Conflict, and Humanitarian Assistance.^31^ South Sudan was the largest recipient country of the United States, followed by the Syrian Arab Republic, Ethiopia, Iraq, Yemen, Somalia, the Democratic Republic of Congo, Sudan, Nigeria, and Bangladesh. In regard to recipient organizations, the WFP received the most, followed by the United Nations High Commissioner for Refugees, the United Nations Children's Fund, Catholic Relief Services and the International Organization for Migration.

For Canada, the total outgoing funding in 2018 was more than 500 million. Most of it went to the food security sector and the health sector. The Syrian Arab Republic was the largest recipient country of Canadian assistance, followed by the Democratic Republic of Congo, Iraq, Lebanon, South Sudan, Bangladesh, Yemen and Ethiopia. In regard to recipient organizations, the WFP was the largest recipient, followed by the United Nations Children's Fund, the International Committee of the Red Cross, the United Nations High Commissioner for Refugees and the United Nations Population Fund.

**Latin America** Latin America is a region exposed to multiple natural crises. There have been various humanitarian assistance programs and initiatives between Latin America and North America and the EU. From 2016, the EU allocated €12.7 million through Disaster Risk Reduction (DRR) projects in Bolivia, Colombia, Ecuador, Paraguay, Peru and Venezuela, which includes €2.25 million in humanitarian assistance towards the 2017 floods in northern Peru and €3 million in response to the 2014 severe floods in Bolivia.^32^ The European Union Civil Protection Mechanism was another project through which Latin American countries received immediate assistance in the face of emergencies and disasters. For example, through this project the EU allocated €5 million to assist Ecuador after the deadly earthquake in 2016. Additionally, this project has assisted Chile in 2017 in response to forest fires, Bolivia in 2016 in response to drought, and Peru in 2017 in response to floods.^33^

Although Latin America is commonly understood as a recipient region of humanitarian assistance, Brazil and Mexico performed well under our measurement, with rankings of 31 and 38 in 2018. Total outgoing funding from Mexico in 2018 was about 1 million dollars, through the United Nations Children’s Fund. The total outgoing funding of Brazil was more than 3 million dollars, through the United Nations Relief and Works Agency for Palestine Refugees in the Near East, the International Organization for Migration, the Food & Agriculture Organization of the United Nations and the International Organization for Migration.

**Africa** Due to the combined effect of food shortages, natural disasters, poverty, conflict and climate change, Africa is in need of humanitarian assistance. “Burkina Faso, Mali and Niger are at the epicenter of one of the world’s fastest-growing humanitarian crises. Vulnerable people living in conflict-hit areas are facing, for the fourth consecutive year, a food crisis due to the overlapping challenges in the region. More than a quarter of the population in the Central African Republic is either internally displaced or living as a refugee in neighboring countries.”^34^ Humanitarian assistance acts as the major support for the survival of a large number of people in Africa. According to the data, among the top twenty affected countries, ten of them are in Africa. They are South Sudan, Somalia, the Democratic Republic of Congo, Nigeria, Ethiopia, Sudan, Central African Republic, Uganda, Niger and Chad.

Besides humanitarian assistance in food and security, the continent also receives foreign aid for infrastructure and energy facilities. China is one of the major donors to African countries. Foreign aid to Africa counts for nearly half of China’s foreign aid. Most of it goes to infrastructure projects, technical assistance and public works. China started to provide foreign aid to Africa in 1955, and this grew steadily between 1973 and 1979. Between 2003 and 2013 54% of China’s overall humanitarian assistance went to sub-Saharan Africa countries, and the three largest recipient countries were Ethiopia, Kenya and Somalia.^35^ In 2017, China provided Nigeria, South Sudan, Kenya and Ethiopia with about 7 million, 14 million, 21 million and 15 million in USD in financial assistance, respectively.

**Oceania** Generally speaking, Oceania performed well in humanitarian assistance in 2018. Australia ranked 30th under our measurement, and New Zealand ranked 32nd. The total outgoing funding of Australia was more than 314 million in USD in 2018. Most of it went to coordination and support services and the food security sector. The largest recipient countries were Bangladesh, Afghanistan, Jordan, the Syrian Arab Republic, Iraq, Lebanon, Myanmar, Tonga and Somalia. Regarding the recipient organizations, the largest recipients were the United Nations High Commissioner for Refugees, the World Food Programme, the Office for the Coordination of Humanitarian Affairs, the International Committee of the Red Cross, and the United Nations Children's Fund. For New Zealand, the total outgoing funding in 2018 was more than 22 million in USD. Most of it went to coordination and support services and the early recovery sector. The largest recipient countries were Yemen, Tonga, Papua New Guinea, Bangladesh, Indonesia, the Syrian Arab Republic, South Sudan, Somalia and Ethiopia. And the largest recipient organizations were the International Committee of the Red Cross, the Central Emergency Response Fund, the World Food Programme, the United Nations High Commissioner for Refugees, and Caritas New Zealand.

#### Conclusion

Humanitarian assistance has long been an important part of global justice. In this section, we measure the contribution of each nation state to providing humanitarian assistance across 11 indicators in all, measuring the donation to different sectors including food, health, water, emergency response, early recovery, coordination, education, protection, agriculture, housing and others (i.e. donations without a designated use). According to our measurement, the top ten countries in 2018 were the United States, Saudi Arabia, the United Arab Emirates, the United Kingdom, Germany, Canada, Kuwait, Japan and Sweden. The United States has maintained first place from 2010 to 2018. Arab countries with strong petroleum reserves and developed countries in Europe also ranked at the forefront.

### Issue 4: Anti-terrorism and Conflicts

#### Introduction

Terrorist activities have been relatively uncommon throughout history, but since the twentieth century terrorism has become a major global problem. Although 20 years have passed since the September 11 incident in 2001, its impact on the world has not yet completely dissipated. With in-depth study of terrorism in countries around the world, our understanding of terrorism is constantly deepening. However, the definition of terrorism has become increasingly vague. Some organizations are recognized as terrorist organizations by some countries, but they cannot be called terrorist organizations according to the standards of other countries. Although differences in this regard cannot be resolved, there is a global consensus on the need to fight against terrorism. This is because not only developed countries face the threat of terrorism, developing countries are also not free from terrorism. Domestic and regional conflicts also occur frequently around the world. Especially in recent years, there have been more and more conflicts caused by the gap between the rich and the poor, economic fluctuations and ethnic conflicts.

Terrorist activities and various armed conflicts are major challenges and threats facing the world today. Countries are under great pressure in countering terrorism and reducing armed conflicts. Currently, regional and local armed conflicts are quite common, resulting in great trauma to the affected countries and people. Meanwhile, terrorist organizations and terrorist activities are very active, threatening regional and world peace and development. Some extremist organizations resort to undifferentiated violence against civilians for various political, cultural and religious reasons. These acts are harmful to global justice. For now, the international community generally believes that national efforts and global cooperation are the main measures to combat terrorism and reduce the threat of conflicts. In this section, we measure the efforts and effectiveness of countries in responding to terrorism and armed conflict in order to measure their contribution to global justice on this issue.

#### Dimensions and Indicators

Our study attempts to measure the contribution of various countries to global justice in response to terrorism and armed conflict. Terrorist attacks and armed conflicts are negatively related to global justice and, therefore, are negative performance measures. If a country is involved in more terrorist attacks and armed conflicts, it means that it contributes less to global justice; by contrast, peace agreements are positively related to global justice and therefore are a positive measurement of contribution. We measure the contribution of various countries to global justice in responding to terrorism and armed conflict across three dimensions: armed conflict, conflict agreements and terrorism (Table 7).

**Table 7:** Data on anti-terrorism and armed conflicts

Category	Dimension	Indicator	Data source	Coverage
Performance	Conflicts	Number of conflicts	UCDP Armed Conflict 192 Dataset	192 2010–2018
		Number of wars		
		Number of conflict deaths		
Contribution	Conflict Agreements	Number of agreements		
		Achievements of agreements		
Performance	Terrorism	Number of terrorism events	Global Terrorism Dataset	
		Number of deaths from terrorism events		

Each dimension has two or three indicators. The armed conflict dimension includes three indicators: number of conflicts, number of wars, and number of conflict deaths. The conflict agreements dimension includes two indicators: number of agreements and achievement of agreements. The terrorism dimension two indicators: number of terrorist events and number of deaths from terrorist events. These data come from the Uppsala Conflict Data Program (UCDP) and the Global Terrorism Database (GTB). Our calculation method is cumulative measurement. For example, if a conflict breaks out between two countries, and there are deaths in the conflict, then the number of conflicts in those two countries in that year would be increased by one, and the number of deaths due to involvement in conflicts would also increase. In addition, in the dimensions of armed conflict and terrorism, we have adopted population-weighted treatment (namely, divided by the country's population), which is conducive to making our research more scientific.

In terms of the conflict agreement, we use the following algorithm to calculate its results. As is well-known, reaching a peace agreement to resolve an armed conflict is very difficult, because a peace agreement often requires long and arduous negotiations and talks. In order to recognize these long-term efforts and contributions, we use retrospective points to assign values to the indicator scores of each peace agreement. For example, assuming that a peace agreement is signed in year *i*, then the score for the same year is *s*. According to the above-mentioned agreement calculation method, in the previous i-1, i-2, i-3 and i-4 years, we will give 0.5 s, 0.3 s, 0.2 s and 0.1 s to the scores for that country, respectively (Table 8).

**Table 8:** Variable code

Indicator	Value	Meaning
mil_prov	0–1	Whether a military agreement is reached
pol_prov	0–1	Whether a political agreement is reached
terr_prov	0–1	Whether an agreement on the territory is reached
justice_prov	0–1	Whether a judicial agreement is reached
outlin	0–1	Whether a negotiation agenda is set
pko	0–1	Whether the agreement specify peace-keeping measures
pa_type	1, 2, 3	Agreement quality:1 = all, 2 = partial, 3 = preliminary

The function to measure “achievements of agreements” is as follows:

Achievements of agreements = mil_prov + pol_prov + terr_prov + justice_prov + outlin + pko + (3 − pa_type) /2.

#### Results

In this section, we present the ranking results of the countries’ contributions to global justice from the conflicts and anti-terrorism perspectives (Table 9). Table 9 shows 9 years of results from 2010 to 2018 in 192 countries.

**Table 9:** Country ranking in the anti-terrorism and conflict aspect of promoting global justice

Country	2010	2011	2012	2013	2014	2015	2016	2017	2018
Ethiopia	24	20	28	20	4	4	1	1	1
Eritrea	3	132	80	79	5	5	2	2	2
South Sudan	2	1	1	1	1	1	3	3	3
China	8	7	7	7	13	7	4	4	4
Japan	10	9	9	8	8	13	7	5	5
Viet Nam	12	10	10	9	6	6	6	7	6
Republic of Korea	32	33	30	23	29	10	9	8	7
Brazil	9	8	8	10	7	8	5	6	8
Uzbekistan	20	17	13	13	12	16	10	9	9
Angola	44	24	16	14	14	14	19	37	10
Poland	45	40	38	28	38	9	16	10	11
Democratic People's Republic of Korea	26	23	17	15	15	15	13	12	12
United Republic of Tanzania	17	13	11	34	37	45	15	19	13
Spain	43	36	36	36	42	11	14	28	14
Kazakhstan	31	79	61	47	17	19	51	15	15
Argentina	35	15	18	16	11	12	12	13	16
Madagascar	29	32	27	27	20	24	26	11	17
Cote d'Ivoire	30	28	20	22	16	25	17	18	18
Romania	73	66	62	56	63	30	24	22	19
Morocco	18	46	12	12	10	38	22	17	20
Cuba	42	44	40	29	31	33	30	24	21
Indonesia	11	26	31	21	18	17	11	14	22
Bangladesh	39	27	23	88	78	120	70	35	23
Dominican Republic	51	52	44	44	45	39	33	45	24
Malawi	37	34	29	40	19	21	18	21	25
Peru	19	18	66	64	55	57	35	44	26
Honduras	58	71	50	72	49	42	48	51	27
Belarus	76	86	72	33	36	41	34	32	28
Germany	13	30	14	11	24	83	66	38	29
Russian Federation	126	112	105	102	56	52	71	53	30
Mexico	16	11	34	25	9	26	8	23	31
Australia	67	62	58	52	90	93	75	70	32
Italy	55	41	49	32	40	18	29	20	33
Zambia	40	38	33	24	23	23	21	31	34
Papua New Guinea	64	60	57	43	44	43	37	95	35
United States of America	22	14	15	17	25	34	40	40	36
Malaysia	53	55	59	75	72	37	73	29	37
Ghana	25	22	37	26	32	32	32	25	38
Cambodia	38	42	32	48	30	40	27	30	39
France	41	48	99	51	59	100	82	85	40
Algeria	143	80	107	105	64	67	39	54	41
Zimbabwe	57	53	35	53	35	28	25	42	42
Azerbaijan	100	95	94	92	101	44	62	76	43
Guatemala	36	35	45	68	22	22	20	16	44
Canada	59	45	53	45	67	54	47	49	45
Lao People's Democratic Republic	69	65	70	50	48	77	88	66	46
Benin	52	54	46	49	43	49	36	43	47
Czechia	93	93	85	93	100	80	55	77	48
Kyrgyzstan	78	69	65	58	52	55	46	50	49
Portugal	92	98	84	82	86	50	38	47	50
Hungary	96	91	88	85	91	46	49	52	51
Turkmenistan	80	72	67	61	76	58	52	55	52
Singapore	119	117	115	111	54	59	53	56	53
Uganda	99	21	55	19	85	56	87	59	54
Slovakia	115	113	114	110	108	61	54	58	55
Congo	85	76	73	84	61	64	144	62	56
Bulgaria	104	109	117	109	102	72	57	41	57
Iran (Islamic Republic of)	86	51	22	39	26	73	59	57	58
Guinea	48	47	41	63	39	66	44	39	59
Costa Rica	81	74	71	66	60	65	61	63	60
Sri Lanka	54	29	51	80	79	74	23	112	61
Oman	97	89	79	74	65	70	64	68	62
Togo	65	63	60	65	53	68	50	67	63
Switzerland	62	82	54	67	41	63	67	60	64
Austria	102	101	95	100	97	60	72	75	65
Panama	89	83	77	73	66	76	81	73	66
Kuwait	98	102	82	78	68	132	114	74	67
Croatia	125	124	121	123	117	71	68	72	68
Serbia	79	61	83	46	46	48	42	61	69
Haiti	50	50	42	31	34	36	56	27	70
Netherlands	83	78	69	62	93	85	84	34	71
Bolivia (Plurinational State of)	49	49	56	30	33	35	31	26	72
El Salvador	71	108	109	107	104	75	60	78	73
United Arab Emirates	105	97	91	97	116	102	101	69	74
India	88	75	68	69	69	78	79	84	75
Senegal	63	94	108	70	58	51	28	33	76
Republic of Moldova	90	85	93	76	70	79	86	81	77
Uruguay	94	88	81	81	73	81	91	83	78
Venezuela (Bolivarian Republic of)	21	19	19	18	28	27	41	79	79
South Africa	14	12	24	41	51	20	65	71	80
Egypt	15	57	76	139	136	157	148	148	81
Norway	123	157	116	114	114	84	69	90	82
Belgium	91	87	89	86	110	96	120	102	83
Jordan	117	68	75	71	118	116	131	123	84
Mongolia	140	141	134	129	129	87	80	88	85
Ecuador	34	43	39	38	21	31	58	36	86
Ukraine	6	4	4	3	2	3	105	105	87
Sweden	106	99	98	95	103	130	111	120	88
New Zealand	124	123	119	115	119	69	76	65	89
Jamaica	101	96	90	90	92	89	83	104	90
Myanmar	60	16	64	54	50	92	108	135	91
Liberia	87	81	87	87	82	90	74	93	92
Rwanda	108	73	103	96	57	47	43	48	93
Denmark	121	111	112	112	128	121	115	80	94
Sierra Leone	66	64	86	77	74	86	85	101	95
Albania	138	139	131	132	135	110	103	97	96
Qatar	112	105	101	98	84	129	119	113	97
Finland	116	114	113	113	111	112	92	96	98
Paraguay	95	100	97	120	121	131	125	109	99
Georgia	152	143	135	127	127	91	110	89	100
Namibia	107	103	102	101	89	97	90	92	101
Botswana	111	106	104	103	94	98	95	94	102
Mauritania	109	116	78	94	77	95	78	91	103
Gabon	120	115	111	108	98	101	99	133	104
Lesotho	110	107	106	104	95	105	97	98	105
Republic of North Macedonia	150	148	150	140	146	123	96	107	106
Slovenia	151	150	144	141	141	99	98	99	107
Sudan	1	3	2	4	150	142	146	134	108
Guinea-Bissau	122	119	125	119	107	114	112	121	109
Lebanon	118	129	140	174	175	161	157	144	110
Turkey	68	92	138	91	99	147	164	130	111
Kenya	74	104	127	125	133	119	109	125	112
United Kingdom of Great Britain and Northern Ireland	103	90	92	117	105	104	107	116	113
Ireland	134	136	157	152	153	145	132	140	114
Tajikistan	84	59	96	60	62	94	63	64	115
Nicaragua	75	67	63	55	80	53	45	46	116
Armenia	139	140	137	131	132	113	124	106	117
Bosnia and Herzegovina	133	134	124	124	131	125	77	100	118
Lithuania	135	137	130	126	130	88	93	103	119
Gambia	113	110	110	106	96	106	104	111	120
Niger	77	58	47	89	75	162	135	126	121
Equatorial Guinea	137	135	128	122	113	111	118	115	122
Timor-Leste	130	130	123	118	112	109	117	117	123
Mauritius	127	126	122	116	109	107	116	114	124
Cyprus	129	128	136	157	143	138	133	119	125
Latvia	149	149	145	142	142	108	100	127	126
Tunisia	46	77	74	130	123	135	127	87	127
Eswatini	131	131	126	121	115	115	121	122	128
Burundi	148	133	100	83	88	168	165	131	129
Chile	72	70	43	57	83	29	89	82	130
Trinidad and Tobago	136	125	120	138	106	117	113	110	131
Greece	154	118	129	149	124	118	126	141	132
Thailand	153	145	146	154	145	133	143	128	133
Saudi Arabia	23	37	52	35	81	141	145	118	134
Fiji	141	142	133	128	122	122	128	132	135
Chad	56	39	48	59	71	156	94	108	136
Pakistan	159	163	164	165	160	155	149	149	137
Estonia	158	161	154	155	154	136	123	137	138
Mozambique	27	25	25	99	87	62	130	86	139
Guyana	144	146	141	133	125	128	140	138	140
Nepal	114	121	118	136	47	103	106	160	141
Solomon Islands	156	152	147	144	134	134	137	143	142
Luxembourg	172	172	169	166	164	137	138	146	143
Suriname	155	153	148	146	137	139	141	147	144
Colombia	142	127	139	137	140	126	122	129	145
Cabo Verde	157	155	149	148	139	140	142	150	146
Bahrain	145	138	176	181	177	174	166	170	147
Democratic Republic of the Congo	7	6	6	6	120	124	134	139	148
Burkina Faso	33	31	26	37	27	82	102	124	149
Comoros	146	147	142	134	126	127	129	136	150
Malta	160	156	152	150	152	154	151	164	151
Djibouti	4	144	153	143	151	152	150	154	152
Bhutan	147	154	143	135	138	143	139	152	153
Brunei Darussalam	161	158	155	151	144	146	153	153	154
Philippines	132	122	132	153	147	151	155	158	155
Nigeria	82	120	151	147	162	163	152	151	156
Bahamas	163	162	156	158	148	148	156	155	157
Belize	164	164	158	160	149	149	158	156	158
Israel	128	160	159	145	173	150	154	142	159
Iceland	179	178	179	173	176	153	160	159	160
Maldives	162	159	160	161	169	160	159	163	161
Vanuatu	167	167	163	159	156	159	163	162	162
Montenegro	168	168	165	167	163	144	136	145	163
Barbados	166	165	161	156	155	158	162	161	164
Cameroon	61	56	21	42	157	166	147	157	165
Sao Tome and Principe	170	170	167	162	158	164	167	166	166
Samoa	169	169	166	163	159	165	169	167	167
Saint Lucia	171	171	168	164	161	167	170	168	168
Mali	47	5	5	5	3	2	161	165	169
Yemen	165	166	171	168	178	181	179	169	170
Kiribati	177	176	174	171	165	170	172	171	171
Central African Republic	5	2	3	2	182	169	171	181	172
Micronesia (Federated States of)	178	177	175	172	168	173	174	172	173
Grenada	175	175	173	170	166	171	173	173	174
Saint Vincent and the Grenadines	174	174	172	169	167	172	175	174	175
Tonga	176	189	189	188	187	175	176	175	176
Seychelles	181	181	178	176	171	177	177	176	177
Antigua and Barbuda	182	180	177	175	170	176	178	177	178
Libya	70	84	162	184	188	188	188	180	179
Andorra	183	182	180	177	172	178	180	178	180
Dominica	184	183	181	179	174	179	181	179	181
Syrian Arab Republic	28	151	170	178	181	187	185	183	182
Iraq	187	186	185	190	192	190	190	189	183
Marshall Islands	185	184	183	182	179	180	182	182	184
Saint Kitts and Nevis	186	185	184	183	180	182	183	184	185
Somalia	173	173	182	180	186	183	186	186	186
Monaco	188	187	187	186	183	185	184	185	187
San Marino	189	188	188	187	185	186	187	187	188
Afghanistan	180	179	186	185	184	184	168	188	189
Palau	190	190	190	189	189	189	189	190	190
Nauru	192	192	192	192	190	191	191	191	191
Tuvalu	191	191	191	191	191	192	192	192	192

The above table shows that from 2010 to 2018, South Sudan, China, Japan, Vietnam, Brazil and other countries have consistently performed well in anti-terrorism and conflicts, ranking among the top 10 globally most of the time. Hot spots such as Tuvalu, Palau, Somalia and Afghanistan have long been at the bottom. The top-ranked countries perform well in the dimensions of counter-terrorism and conflicts, mainly because they are rarely involved in large or small conflicts, and their domestic terrorism is well governed and there is almost no record of casualties in terrorist activities. In other countries, the signing of peace agreements in recent years ended or ameliorated long-standing armed conflicts, which has also greatly improved their scores and ranks among the top few, for example as in Ethiopia, Eritrea and South Sudan, which ranked the top three in 2018. In addition to those low-ranking countries which still frequently suffer from domestic and international turmoil and have more people who died from terrorist attacks and domestic and foreign conflicts, some countries rank lower simply due to having a smaller population. For example, according to our population-weighted algorithm, the average number of deaths and other indicators score particularly lowly for, Palau, Nauru and Tuvalu, which were at the bottom in 2018.

Compared with other topics, there are more fluctuations in the ranking of countries in the field of counter-terrorism and conflict. Countries with significant changes in national rankings are roughly divided into two categories. Sudden changes are often due to the signing of peace agreements. As mentioned previously, these include countries such as Eritrea, Ukraine, and Sudan. The score for the year with the peace agreement often differs from the year without such an agreement by up to 100 places. The main reason why the signing of a peace agreement has such a big impact on the ranking is that the score gap between countries on the two dimensions of anti-terrorism and conflict is not very large. In this case, the difference in the scores of the conflicting agreement dimensions can have a major impact on the final ranking. The logic behind this algorithm is that we hope that more countries can sign agreements to end states of conflict, because regional conflicts and conflicts between countries have a very negative impact on global justice. In addition to some countries with sudden changes in rankings, there are also some countries with large but relatively slow ranking changes, such as Singapore, Russia and Nicaragua. The main reason for the changes in the ranking of these countries is the increase or decrease in the number of casualties due to conflicts and terrorist attacks. This can lead to large changes in the rankings of these countries, which are about 50 points, between 2010 and 2018.

China's ranking was basically stable from 2010 to 2018, with relatively small changes in casualties caused by terrorist attacks and conflicts, and as a result it has long ranked among the top 10 in the world. The Chinese government attaches great importance to social stability, national security and social harmony and stability. Because of the state's emphasis on and investment in counter-terrorism, terrorist activities rarely occur in China, and casualties caused by terrorism are relatively small. South Sudan is a small country in Africa. It became a new sovereign country after gaining independence from Sudan in 2011. Two years after South Sudan's independence, internal conflict broke out in the country, and its society entered into a state of unrest. As such, South Sudan's performance in the dimension of counter-terrorism and conflict should be relatively poor. However, the two sides of the South Sudan civil war signed a peace agreement twice in 2015 and 2018, and they have always hoped to end the civil war through a peace agreement. This has led to serious internal conflicts in South Sudan, but it was able to achieve a very high ranking because of the signing of the peace agreements. The main reasons why countries such as Japan, Vietnam, and Brazil rank very high are that they are rarely involved in regional conflicts, their domestic social situations are very stable, and the number of terrorist activities is very small.

Germany's ranking displayed a downward trend and fluctuated greatly from 2010 to 2018. During this period, Germany was not involved in conflicts with other countries. The main reason why the ranking has shown a downward trend and fluctuated greatly is the increase in terrorist attacks in Germany. For example, in July 2016, Germany experienced a series of violent terrorist attacks over a short period of time, making Germany a "new disaster zone" for terrorism. Some Germans believe that these terrorist activities are related to Germany's open refugee policy. The combination of refugee issues and terrorism has made Germany's domestic security situation very serious. It is worth noting that countries such as Spain, France and Turkey are also facing a similar situation to Germany. Terrorist incidents such as the truck crash in Barcelona, ​​Spain in 2017, the truck crash in Nice on the 2016 French National Day, the 2015 terrorist attack in Paris and the 2017 Turkish nightclub shooting have repeatedly reminded us that European countries are facing a very large amount of terrorism.

The ranking of the United States displayed a downward trend from 2010 to 2018. The United States has the most powerful military in the world, and it is also the world's police force. It has launched and intervened in many wars and regional conflicts over this century. From this perspective, the performance of the United States in conflict is l not satisfactory. However, over the past 10 years, the United States has gradually withdrawn from many regions, deliberately reducing its military power abroad. Moreover, the United States has been stepping up its crackdown on terrorist activities. For example, the United States has successively killed Al Qaeda leader Osama bin Laden and Islamic State leader Baghdadi, making great contributions to anti-terrorism worldwide. The combination of these factors prompted the United States to hover around 30 in the rankings. Although the United States has achieved good results in foreign counter-terrorism, there is no decline in domestic terrorist activities.^36^ Gun attacks have occurred repeatedly, causing large casualties. For example, the 2017 Las Vegas shooting killed 59 people and injured hundreds. This was the most serious case of shooting in the history of the United States. Although it was not ultimately classified as a terrorist attack, it was almost the same as many terrorist attacks in terms of modus operandi and process. The United States will still face many challenges relating to domestic terrorist attacks in future.

The UK was ranked at around 100 from 2010 to 2018, and is relatively lagging among major countries. In many aspects of foreign policy, Britain pursues a policy of following the United States. For example, in the 2003 Iraq War, the United Kingdom resolutely invaded Iraq with the United States despite opposition from many quarters. Facts have proved that the policy of following the United States to war subsequently brought many problems and troubles to Britain. The increase in terrorist attacks is one of the bitter consequences of Britain’s intervention in the Iraq War. The 2005 London bombing in England shocked the world. In 2005, several London Underground stations and buses exploded, causing 56 deaths and more than 700 injuries. Since then, the cloud of terrorism has been hanging over Britain. According to British media reports, from 2010 to 2017, a total of 2,029 terrorists were arrested in the UK. As of the end of June 2018, the British MI5 and counter-terrorism departments have conducted a total of 676 investigations into various terrorism cases. The Minister of State for Security, Ben Wallace, said that since 2017, the number of terrorists, terrorist attacks and the number of cases under investigation have increased, and the UK's counter-terrorism situation is not optimistic.^37^

India has been ranked around 70 for a long time from 2010 to 2018. India's lagging position is due to the border conflict with Pakistan on the one hand, and the growing threat of terrorism on the other. Data from the Global Terrorism Database (GTD) shows that from 1970 to 2017, there were nearly 180,000 terrorist attacks around the world, of which 31,959 terrorist attacks occurred in South Asia, accounting for about 18% of the total. More importantly, the proportion of attacks occurring in South Asia has been increasing year by year in recent years. In 2017, for example, South Asia suffered 3430 terrorist attacks, accounting for 31% of the total.^38^ India is the most important country in South Asia and has suffered the most terrorist attacks. The reason for this is that India’s complexity with regard to religion, ethnic group, caste, language, etc. and its proximity to Middle Eastern countries, make it easy for terrorist organizations to target.

From 2010 to 2018, Russia's ranking was on a sharp upward trend. Russia suffered many terrorist attacks at the beginning of this century. Chechen terrorists (exemplified by the "Black Widow") planned and carried out many terrorist attacks, including the hostage-taking incident in the Moscow Palace of Culture in 2002. Over the past 10 years, although the terrorist attacks carried out by Chechen terrorists have not completely stopped, the number and scale have decreased a lot. Although Russia's performance in counter-terrorism has improved, its performance in the field of conflict has been relatively poor. Russia initiated and intervened in wars with Georgia, Ukraine, and Syria in 2008, 2014, and 2015. The occurrence of these wars led to Russia ranking outside the world's 100 prior to 2013. In recent years, with the reduction of conflicts in wars, Russia's ranking in the field of counter-terrorism and conflict has risen sharply.

#### Regional Analysis

**Fig. 7:**
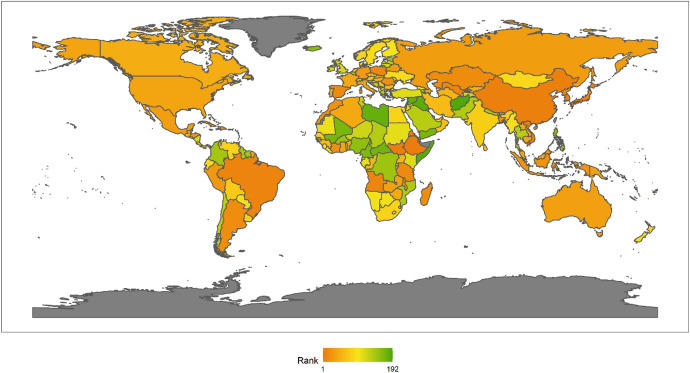
2018 Index ranking of conflicts and terrorism on a world map

In 2018, as mentioned earlier, three African countries ranked in the top three due to the conclusion of agreements (Fig. 7). Among the top ten countries, there are countries that have traditionally performed well, including China, Japan, Vietnam, Brazil, etc., and there are also countries that have steadily increased in recent years, such as South Korea and Uzbekistan. Among the bottom-ranked countries, Afghanistan, Somalia and Iraq are the most populous countries. Syria, Egypt, Papua New Guinea, Sri Lanka, France and Myanmar rose more than 40 places in the rankings, while Nicaragua, Mozambique, Tajikistan, Ecuador, Chile, Bolivia, Rwanda, Senegal and Haiti fell more than 40 places.

Next, we also classify countries according to their continents. These continents include Asia, Europe, North America, Latin America, Africa and Oceania. The ranking of each continent is obtained by calculating the average of the rankings of these countries. We drew a line chart to visually present and compare the differences in the contributions of various continents to counter-terrorism and conflict.

**Fig. 8:**
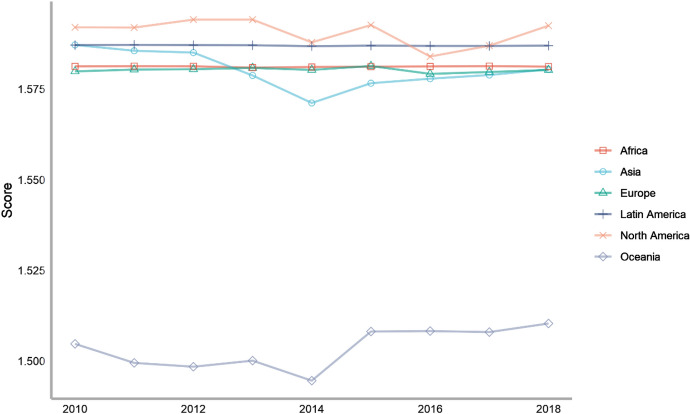
The score of Anti-terrorism and conflicts issue across continents, 2010–2018

From the above figure, we can find that taking 2018 as an example, in terms of counter-terrorism and conflict, if we compare the average performance of countries on each continent, African countries have contributed the most, followed by North American countries, European countries, Latin American countries and Asia. The countries in Oceania have contributed the least (Fig. 8). The main reason why African countries rank high overall is that the frequency of domestic terrorist activities is relatively low and the number of peace agreements they have signed is relatively low. North American countries only include the United States and Canada, and their performance is slightly lower than that of African countries. The main reason why Oceanian countries rank at the bottom, is that some island countries are lagging behind, and there is no country in the region that has performed particularly prominently in the field of counter-terrorism and conflict.

**Asia** Most of the countries with stable top rankings are in Asia, and half of the top 10 countries globally are in Asia. This clearly shows that Asian countries have performed very well in counter-terrorism and conflict. The top 5 Asian countries are China, Japan, Vietnam, South Korea and Uzbekistan. With the exception of Uzbekistan, which is a Central Asian country, the other four are all East Asian countries. China, Japan, and South Korea are also major economies. China, Japan and South Korea are relatively close in culture, and they all pay attention to domestic societal harmony and stability. These countries have made great achievements in preventing terrorist attacks. Therefore, large-scale terrorist attacks have rarely occurred in the past few years. Yemen, Syria, Iraq, and Afghanistan rank at the bottom in Asia, which is consistent with their domestic political chaos and rampant terrorist activities. In the future, Asian countries may continue to show this kind of polarization. Some Asian countries, represented by East Asian countries, continue to perform well in anti-terrorism and conflict, while Middle Eastern countries tend to continue to rank behind because of domestic political instability and the threat of terrorism.

**Europe** European countries generally rank in the middle reaches of the world rankings, and none of them clearly rank among the top 30. The top 5 European countries in the field of counter-terrorism and conflict include Poland, Spain, Romania, Belarus and Germany. The reason why Poland ranks No. 1 in Europe is mainly due to its stable domestic environment and few terrorist attacks. While Western European countries are suffering from terrorist attacks, Poland has not had a terrorist attack for a long time. The Polish government and people hold a negative attitude towards accepting refugees and have a strong sense of guarding national borders, thus reducing the chance of many terrorists entering Poland. The last five European countries in the field of counter-terrorism and conflict are Iceland, Montenegro, Andorra, Monaco and San Marino. These countries are small in terms of population and land area, and are affected by our population weighting algorithm and, therefore, perform poorly in rankings. In terms of the absolute number of terrorist events and conflicts, there are not many terrorist attacks or conflicts in these countries, and they are peaceful and safe.

Western European and Nordic countries have performed poorly in counter-terrorism. In the twenty-first century, Western Europe has been an important target of extreme terrorist attacks. Two terrorist forces have emerged in Western Europe. The first force is Islamic extremism and religious fundamentalists. These people often have close ties with Al Qaeda, the Islamic State and other Islamic extremists and fundamentalists in the Middle East and North Africa. The 2004 Madrid train bombings in Spain and the 2005 London bombings that shocked the world are typical cases in this regard. The London bombing in the UK in 2005 greatly changed the thinking of European countries in response to the threat of terrorism.^39^ Such terrorist activities have been widely reported in the outside world, and they are also a main focus in Europe. The second force of terrorism is the indigenous Racially or Ethnically Motivated Terrorism (REMT). This type of terrorism is a terrorist attack against other races and ethnic groups launched by people with extreme hatred and desire to exclude. The 2011 bombings and shootings in Norway are typical cases of such terrorist activities. The 32-year-old Norwegian Anders Behring Breivik has strong ideals of ​​xenophobia and Christian terrorism. He meticulously prepared and carried out an explosion against a Norwegian government office building and shootings against hundreds of teenagers, which eventually caused a tragic f 69 deaths and 66 injuries. This terrorist attack was the largest attack that Norway had suffered since its invasion by Germany in World War II. Because this terrorist attack was planned by one person independently, it has also been called a lone-wolf terrorist attack.^40^ Lone-wolf terrorist attacks are proliferating in Europe and other regions, and they are a new serious challenge for the world's anti-terrorism efforts.

**North America** In 2018, among North American countries, the United States outperformed Canada in counter-terrorism and conflict. The United States has rapidly stepped up its crackdown on terrorist activities since the September 11 terrorist attacks in 2001. Bin Laden's Al Qaeda and the later rise of the Islamic State (ISIS), the two core Islamic terrorist forces, were basically eliminated by the United States. It is expected that Canada's performance in counter-terrorism and conflict is slightly worse than that of the United States. Canada has actively participated in US-led foreign wars and conflicts, but its performance in counter-terrorism has been weaker than that of the US. Therefore, Canada lags behind the United States in rankings. At the same time, the United States and Canada both face the threat of indigenous ethnic terrorism. But the United States may face more threats than Canada.^41^

**Latin America** In 2018, we found that the performance of Latin America was the same as that of Asia. The top five countries in the region are Brazil, Argentina, Cuba, Dominica and Peru, while the countries that lag behind are the small Caribbean countries such as Antigua and Barbuda, Dominica and Saint Kitts and Nevis. In the field of counter-terrorism and conflict, the regional powers of Latin America outperformed the small countries in the region. There are not many terrorist activities in Latin America as a whole, but the regional situation is not very peaceful either. There are armed conflicts and large-scale protests in countries such as Venezuela, Bolivia, Peru and Colombia. The internal situation of those small island countries that are lagging behind is not very stable, and because of the existence of population weighting factors, although they have not experienced many conflicts and terrorist attacks, they lag behind in relative terms.

**Africa** In 2018, we found that the top 5 African countries were Ethiopia, Eritrea, South Sudan, Angola and Tanzania. These countries are also among the highest in the world rankings in the field of counter-terrorism and conflict. Ethiopia, Eritrea, and South Sudan were able to rank in the top 3 mainly because of the peace agreements they have signed, which ended past conflicts. Compared with other regions, the security situation in Africa is relatively unstable and there are more variables. Take Ethiopia as an example: although the country ranked first in the world and Africa in 2018, its ranking was out of top 20 before 2014. In 2020, conflicts between the federal government and local state governments broke out in Ethiopia, causing hundreds of casualties. If the situation is not effectively controlled, Ethiopia's latest ranking will drop significantly in future. The situation in Ethiopia is a relatively common phenomenon in African countries. Although Africa is also facing the threat of terrorism, on the whole, it is the internal conflict and security situation that affects the performance of African countries in the field of counter-terrorism and conflict. Conflicts caused by ethnic, cultural, religious, resource and territorial issues are likely to plague African countries for a long time.

**Oceania** In 2018, we found that the top 3 countries in Oceania are Australia, Papua New Guinea and New Zealand. At the bottom of the ranking are small island countries such as Palau, Nauru, and Tuvalu. Oceanian countries such as Australia and New Zealand have long given the outside world the impression of being very harmonious and stable. But in recent years, the threat of terrorism has also shown an upward trend in the region. Terrorist attacks carried out by Islamic extremists in Europe and terrorist attacks caused by indigenous racism and xenophobia have also appeared in countries such as Australia and New Zealand. For example, in March 2019, the Christchurch Mosque shooting occurred in New Zealand. The incident resulted in 51 deaths and 49 injuries, shocking the world. New Zealand is a country that allows citizens to own guns, and gun controls are relatively light. Although the New Zealand shooting in 2019 was not included in our research, this terrorist act is similar to the shooting carried out by the Norwegian Anders Behring Breivik, and belongs to the homegrown category of racially and ethnically motivated terrorism. Both New Zealand and Australia are countries that welcome refugees, and refugee policy has become a major issue within these countries. If it is not handled properly, it may trigger more terrorist attacks.

#### Conclusion

Terrorism and conflicts have deep religious, ideological and cultural roots,^42^so it is difficult to eliminate them in a short time. Both developed and developing countries will face long-term threats from terrorism and conflict. The terrorist threat caused by Islamic extremism will continue to pose a major threat to Europe and North America in the short term. At the same time, local racial and ethnically driven terrorism may develop further. In the context of xenophobia, racial hatred and anti-globalization sentiments, the developmental momentum of indigenous terrorism may surpass Islamic terrorism. Regional and internal conflicts are also issues that need to be focused on in the future. In the context of the raging novel coronavirus pandemic, the gap between rich and poor and social conflicts may be further widened and intensified, which will cause more regional conflicts and domestic social conflicts. In recent years, some countries in Latin America have faced intensified internal conflicts. The "Black Lives Matter" movement in the United States has also caused many conflicts and confrontations in the country. In the future, these issues may further ferment and affect more countries.

### Issue 5: Cross-national Criminal Police Cooperation

#### Introduction

Transnational crimes, i.e. crimes that involve more than one country in their planning and organization and have effects across national borders, has been called “the dark side of globalization”.^43^ Taking advantage of economic liberalization, technological progress and the freer movement of money, goods and services, “profit-driven crime (e.g., money laundering, drug trafficking, gaming and the sex trade) responds—much like legitimate economic activity—to local regulation by shifting to the territorial jurisdictions in which it incurs lower expected sanctions, making it most profitable for criminals”.^44^ As a response to the explosive growth of transnational crime, there is increasing cooperation and collaboration between national governments and organizations to combat transnational crime, which has resulted in the rise of the international legal field of global crime control.^45^ However, different national governments react to the international mobility of criminal activities differently. Broude and Teichman distinguish between two types of transnational crime that relate to different incentives of the government to adopt control policies. The first type is “insourcing crime”, “whose production process might carry economic benefits so that some national governments have an incentive to adopt lenient crime control policies towards [those crimes]”; the other one is “outsourcing crime”, which are those crimes “the jurisdictions will perceive as harmful” and “undesirable” and thus adopt “harsher sanctions at the domestic level”.^46^ However, because of the suggested cross-national harm to citizens, transnational crimes have caused serious challenges to global justice, and we argue that it is the obligation of national governments to facilitate international cooperation to combat criminal activities no matter whether these activities are insourcing crime or outsourcing crime. Fighting transnational crimes is a major domain of global cooperation to improve global justice. As a result, we include this issue in our global justice index and measure each country’s contributions to fighting transnational crimes. In the following sections we elaborate our indicators, methods and results.

#### Dimensions and Indicators

Last year, we used two major categories to measure transnational criminal cooperation, and each comprised several indicators. The first category, contribution, measures financial contribution to Interpol (the International Criminal Police Organization), and the second category, performance, is the ratification status of each country of UN treaties relating to cooperation against transnational crime. This year, we added seven new indicators to refine the index. First, we make the measurement of performance more detailed: instead of focusing merely on the general UN treaties, we examine various types of criminal activities, including drug trafficking, corruption and hostage-taking. Second, we extend the measurement of contribution by including donations to UNODC (United Nations Office on Drugs and Crime) and FATF (Financial Action Task Force) membership, in addition to the initial calculation of donations to Interpol. A summary of the newly added indicators is given below (Table 10).

**Table 10:** Newly added indicators of cross-national criminal police cooperation

Indicators	Country coverage	Year coverage
Donation to United Nations Office on Drugs and Crime (UNODC)	Full coverage (184) (45 donors)	2018
Membership of the Financial Action Task Force (FATF)	Full coverage (184) (39 member states)	2018
Treaty ratification of the Single Convention on Narcotic Drugs of 1961 as amended by the 1972 Protocol	Full coverage (184) (54 signatories)	1972 ~ 2018
Treaty ratification of the Convention on Psychotropic Substances of 1971	Full coverage (184) (34 signatories)	1971 ~ 2018
Treaty ratification of the United Nations Convention against Illicit Traffic in Narcotic Drugs and Psychotropic Substances of 1988	Full coverage (184) (87 signatories)	1988 ~ 2018
Treaty ratification of the United Nations Convention against Corruption	Full coverage (184) (140 signatories)	2003 ~ 2018
Treaty ratification of the International Convention Against the Taking of Hostages	Full coverage (184) (39 signatories)	1979 ~ 2018

As a result, we now have 14 indicators in all. For the performance category, we measure the ratification status of each country to the UN treaties. These treaties include general treaties against transnational organized crime (United Nations Convention against Transnational Organized Crime, Protocol to Prevent, Suppress and Punish Trafficking in Persons, Especially Women and Children, supplementing the United Nations Convention against Transnational Organized Crime, Protocol against the Smuggling of Migrants by Land, Sea and Air, supplementing the United Nations Convention against Transnational Organized Crime, Protocol against the Illicit Manufacturing of and Trafficking in Firearms, Their Parts and Components and Ammunition, supplementing the United Nations Convention against Transnational Organized Crime), treaties against drugs and psychotropic substances (Single Convention on Narcotic Drugs of 1961 as amended by the 1972 Protocol, Convention on Psychotropic Substances of 1971, United Nations Convention against Illicit Traffic in Narcotic Drugs and Psychotropic Substances of 1988), the treaty against corruption (United Nations Convention against Corruption) and the treaty against taking of hostages (the International Convention Against the Taking of Hostages).

These treaties request nation states to take a series of measures to cooperatively combat transnational organized crimes, including information sharing, adopting legal frameworks in favor of law enforcement cooperation, and police force and expert training plans. It also provides legal and technical assistance to help nation states to build and upgrade the necessary capacity.^47^ For example, in regard to money laundering, the UN Convention against Transnational Organized Crime stipulates that each state “shall institute a comprehensive domestic regulatory and supervisory regime for banks and non-bank financial institutions and, where appropriate, other bodies particularly susceptible to money-laundering, within its competence, in order to deter and detect all forms of money-laundering, which regime shall emphasize requirements for customer identification, record-keeping and the reporting of suspicious transactions”.^48^ As a result, ratification of the treaties denotes compliance with the related requirements and the promise to offer relevant assistance. Moreover, the ratification status of each country also shows their contribution to cross-national cooperation on crime.

For the category of contribution, we measure donations to Interpol donations to UNODC and FATF membership. First, as we discussed in our report last year, since the combatting of transnational crime relies on the actions of more than one country, it is necessary to have an international organization with a well-established communication system to connect all countries, which is what Interpol does. Interpol is the biggest organization in the world t providing technical and operational support in the fight against transnational crime.^49^ As a result, financial donations to Interpol reflect the determination and contribution of a country to transnational cooperation on crime. Secondly, we include donations to UNODC this year. UNODC is also an important organization in the international cooperation against organized crime. It organizes seminars, conferences and working groups under the Organized Crime Convention, which brings together specialists and scholars with relevant expertise and experience to promote the implementation of the Convention. It also provides technical assistance and a platform for cooperation to strengthen governments’ capabilities in combating organizational crimes.^50^ In this sense, financial donations to UNODC facilitate transnational cooperation on crime. Third, we include FATF membership in our measurement, which plays a major role in the global effort to tackle money laundering.

Please see below the details of all the indicators in our measurement of global cooperation against transnational crime.

As shown in Table 10, due to data limitations, we obtained the data for 2018 for the indicators of donations to UNODC and FATF membership; for the rest of the indicators, we obtained the data from 2010 to 2018 (Table 11). As a result, we generate two versions of the rankings for this issue. In the first version (see Table 12), we include all of the indicators to provide a more precise result of each countries’ performance and contribution to police cooperation against transnational crime. However, due to the inconsistency in the indicators’ year coverage, there is an obvious variation between the results of 2017 and 2018. As a result, we provide a complementary second version. In the second version (see Table 13), we remove these two indicators (donation to UNODC and FATF membership) to ensure year coverage consistence. This version of the ranking provides a more accurate account of trends in each countries’ ranking over time.

**Table 11:** Indicators of police cooperation against transnational crime

Issue Area	Category	Dimension	Indicator
Cross-national Criminal Police Cooperation	Performance	General	United Nations Convention against Transnational Organized Crime
			Protocol to Prevent, Suppress and Punish Trafficking in Persons, Especially Women and Children, supplementing the United Nations Convention against Transnational Organized Crime
			Protocol against the Smuggling of Migrants by Land, Sea and Air, supplementing the United Nations Convention against Transnational Organized Crime
			Protocol against the Illicit Manufacturing of and Trafficking in Firearms, Their Parts and Components and Ammunition, supplementing the United Nations Convention against Transnational Organized Crime
		Drugs and Psychotropic Substances	Single Convention on Narcotic Drugs of 1961 as amended by the 1972 Protocol
			Convention on Psychotropic Substances of 1971
			United Nations Convention against Illicit Traffic in Narcotic Drugs and Psychotropic Substances of 1988
		Corruption	United Nations Convention against Corruption
		Taking of Hostages	International Convention Against the Taking of Hostages 1979.12.17
	Contribution	Donation to Interpol	Donation to Interpol / GDP per capita
		Donation to UNODC	General purpose fund / GDP per capita
			Special purpose fund / GDP per capita
			Pledges / GDP per capita
		FATF membership	The Financial Action Task Force Membership

**Table 12:** Country ranking in police cooperation against transnational crime (version 1)

Country	2010	2011	2012	2013	2014	2015	2016	2017	2018
United States of America	1	1	1	1	1	1	1	1	1
Japan	5	5	3	2	2	2	2	2	2
Germany	2	2	2	3	3	3	3	3	3
United Kingdom of Great Britain and Northern Ireland	4	4	4	4	4	6	5	5	4
Sweden	9	11	10	10	11	14	15	15	5
Belgium	12	14	14	16	16	17	20	20	6
Italy	6	6	6	6	5	4	6	6	7
Greece	42	12	11	11	10	12	13	14	8
Finland	13	15	15	17	17	18	18	19	9
New Zealand	18	19	21	22	20	23	21	22	10
Brazil	11	13	13	13	13	9	7	7	11
Norway	22	24	26	26	26	26	27	27	12
Luxembourg	41	43	32	33	33	32	32	30	13
Canada	16	17	16	15	15	16	16	16	14
China	3	3	5	5	6	5	4	4	15
France	8	8	8	8	8	8	9	9	16
India	17	7	7	7	7	7	8	8	17
Netherlands	20	20	20	20	21	22	24	25	18
Austria	23	25	25	25	28	28	29	32	19
Portugal	30	32	30	30	29	30	30	31	20
Switzerland	26	28	29	31	32	33	33	33	21
Turkey	29	31	33	34	34	27	26	21	22
Russian Federation	25	33	34	29	25	11	10	10	23
Israel	34	36	37	37	38	40	40	40	24
Argentina	21	27	28	28	31	35	36	39	25
Australia	35	38	39	39	39	37	37	37	26
Mexico	28	22	19	14	14	13	12	13	27
Spain	31	29	23	21	23	21	23	24	28
Denmark	45	46	47	48	48	49	50	48	29
Indonesia	44	45	45	44	41	38	38	35	30
Egypt	10	10	12	12	12	15	14	11	31
Togo	7	9	9	9	9	10	11	12	32
Chile	15	18	18	19	18	19	17	17	33
Republic of Korea	101	93	91	87	95	44	45	47	34
South Africa	59	59	59	54	53	50	52	54	35
Philippines	14	16	17	18	19	20	19	18	36
Senegal	19	21	22	23	22	24	22	23	37
Guatemala	24	26	27	27	27	29	28	28	38
Panama	27	30	31	32	30	31	31	29	39
Haiti	57	23	24	24	24	25	25	26	40
Saudi Arabia	82	83	87	84	86	85	83	84	41
Honduras	33	35	36	36	35	36	35	34	42
Jamaica	36	37	38	38	37	39	39	38	43
Malaysia	100	103	102	101	102	102	101	100	44
Mauritius	37	39	40	40	40	41	41	41	45
Ireland	106	106	107	108	111	116	120	121	46
Gabon	40	42	43	45	44	42	43	42	47
Lesotho	43	44	44	42	42	43	42	43	48
Ghana	96	102	46	50	47	48	48	46	49
Bolivia (Plurinational State of)	46	48	50	52	51	51	53	51	50
El Salvador	48	50	51	53	52	53	55	53	51
Paraguay	50	52	52	56	54	57	56	56	52
Dominican Republic	51	51	53	55	55	58	57	57	53
Costa Rica	52	53	56	59	57	59	58	59	54
Poland	32	34	35	35	36	34	34	36	55
Liberia	38	41	42	43	43	45	44	44	56
Singapore	143	145	147	147	149	142	142	142	57
Ukraine	58	60	63	65	62	55	54	55	58
Hungary	53	55	55	58	60	60	59	60	59
Peru	56	57	60	61	63	63	62	61	60
Nicaragua	54	56	58	60	61	62	63	64	61
Cote d'Ivoire	123	114	73	76	79	84	84	62	62
Tunisia	61	61	65	66	66	65	66	65	63
Belarus	63	64	67	68	69	67	67	69	64
Ecuador	62	62	66	67	67	68	69	71	65
Trinidad and Tobago	64	65	69	70	71	70	71	73	66
Cyprus	65	66	70	71	70	69	72	75	67
Colombia	95	101	103	104	104	96	93	93	68
Afghanistan	89	88	92	85	73	73	75	52	69
Madagascar	49	49	48	47	46	46	47	50	70
Sierra Leone	102	99	105	120	56	52	51	49	71
Democratic Republic of the Congo	39	40	41	41	45	47	46	45	72
Sudan	126	124	123	124	101	105	100	94	73
Rwanda	55	54	57	57	59	61	61	63	74
Nigeria	73	73	76	78	78	77	70	66	75
United Republic of Tanzania	60	58	61	64	64	64	68	72	76
Algeria	74	75	77	77	77	74	73	74	77
Uganda	66	63	64	63	65	66	65	67	78
Cameroon	69	69	71	72	74	71	76	76	79
Zambia	72	72	74	75	75	72	74	77	80
Kuwait	79	81	85	88	87	87	86	82	81
Cuba	128	129	132	83	85	86	87	85	82
Suriname	81	80	84	89	90	89	88	86	83
Bulgaria	78	79	83	86	88	88	89	87	84
Uruguay	80	82	86	90	92	90	90	90	85
Bahrain	83	84	90	93	94	92	91	91	86
Morocco	97	76	82	82	82	82	81	80	87
Malawi	68	67	54	46	50	56	49	58	88
Jordan	84	85	89	92	93	93	92	92	89
Pakistan	71	70	72	69	72	76	77	78	90
Central African Republic	70	68	68	51	58	54	60	68	91
Mozambique	67	71	75	73	76	75	64	70	92
Ethiopia	98	89	62	62	68	78	80	83	93
Burkina Faso	75	77	80	80	81	79	78	81	94
Mali	77	78	81	81	83	81	85	88	95
Guinea	76	74	78	79	80	83	82	89	96
Myanmar	88	92	95	94	97	98	99	98	97
Timor-Leste	85	86	88	95	96	99	102	99	98
Benin	90	91	94	97	99	94	94	95	99
Kyrgyzstan	86	90	96	98	98	95	97	96	100
Czechia	138	142	142	99	91	91	95	97	101
Kenya	87	87	93	96	100	100	103	103	102
Sao Tome and Principe	91	94	97	100	103	104	104	104	103
Libya	105	97	109	107	106	97	96	101	104
Slovakia	93	100	100	105	108	106	106	108	105
Lao People's Democratic Republic	92	95	101	103	105	107	107	107	106
Romania	110	110	111	115	117	110	108	106	107
Djibouti	99	98	99	106	109	108	109	110	108
Angola	151	154	154	148	120	118	116	118	109
Cabo Verde	108	108	108	110	112	111	112	112	110
Iraq	114	116	119	122	124	117	113	111	111
Azerbaijan	117	118	120	123	125	122	114	114	112
Mongolia	104	109	113	113	115	114	118	115	113
Eswatini	152	153	114	112	114	113	115	117	114
Armenia	107	107	110	111	113	112	117	116	115
Republic of Moldova	103	105	106	109	110	109	110	113	116
Albania	111	111	112	114	116	115	119	119	117
Croatia	118	119	122	125	126	125	125	120	118
Namibia	115	115	117	119	121	121	121	122	119
Republic of North Macedonia	112	112	115	118	122	120	124	125	120
Bosnia and Herzegovina	113	113	116	117	119	119	122	124	121
Serbia	116	117	118	121	123	123	123	123	122
Lithuania	121	123	126	129	130	128	127	126	123
Latvia	120	122	125	128	128	127	128	127	124
Seychelles	119	120	124	127	127	129	129	128	125
Barbados	165	164	167	167	129	130	130	129	126
Malta	122	125	127	130	132	131	131	130	127
Monaco	124	126	128	131	133	132	132	132	128
Iceland	172	171	172	175	175	175	175	176	129
Yemen	125	121	121	126	131	126	111	105	130
Lebanon	129	130	133	136	138	136	136	134	131
Brunei Darussalam	130	131	134	137	139	138	137	137	132
Thailand	139	139	143	116	118	124	126	131	133
Zimbabwe	131	132	136	133	135	133	133	133	134
Niger	109	104	104	102	107	103	105	109	135
Viet Nam	146	146	131	135	137	135	135	135	136
United Arab Emirates	132	133	135	138	142	139	139	138	137
Sri Lanka	140	143	145	145	146	141	140	140	138
Qatar	134	136	139	142	144	145	144	144	139
Cambodia	127	128	130	134	136	137	138	139	140
Mauritania	133	134	137	140	141	140	141	141	141
Burundi	145	141	79	74	84	101	98	102	142
Nepal	136	127	129	132	134	134	134	136	143
Guyana	137	137	141	143	145	146	147	146	144
Comoros	135	135	138	141	143	143	143	143	145
Bahamas	142	144	146	146	147	148	148	148	146
Gambia	144	138	140	139	140	144	145	145	147
Tajikistan	141	140	144	144	148	147	146	147	148
Kazakhstan	161	162	164	166	166	162	153	153	149
Papua New Guinea	147	148	151	152	152	150	151	150	150
Bhutan	149	149	150	151	151	151	152	151	151
Georgia	150	151	152	154	155	153	154	154	152
Belize	153	152	153	153	154	154	155	155	153
Oman	159	160	161	163	163	156	156	156	154
Fiji	179	182	182	185	185	184	183	157	155
Turkmenistan	154	155	157	160	160	157	157	158	156
Saint Vincent and the Grenadines	155	156	155	156	156	160	159	159	157
Dominica	181	184	184	157	157	164	163	160	158
Botswana	156	159	158	159	159	159	161	163	159
Slovenia	160	161	160	162	161	163	160	161	160
Montenegro	157	158	156	158	158	158	158	162	161
Nauru	180	183	162	164	164	161	162	164	162
Maldives	174	175	175	173	172	171	164	165	163
Grenada	158	157	159	161	162	165	165	166	164
Estonia	163	166	168	169	169	167	168	168	165
Antigua and Barbuda	162	163	165	165	167	166	167	167	166
Saint Kitts and Nevis	164	165	166	168	168	168	169	169	167
San Marino	166	167	169	170	170	169	170	170	168
Kiribati	167	168	170	171	171	170	171	171	169
Bangladesh	169	147	149	150	153	155	166	172	170
Chad	168	169	163	155	165	152	150	149	171
Guinea-Bissau	148	150	148	149	150	149	149	152	172
Uzbekistan	170	170	171	172	173	172	172	173	173
Equatorial Guinea	173	173	173	176	176	174	173	174	174
Saint Lucia	182	185	185	174	174	173	174	175	175
Micronesia (Federated States of)	175	174	174	177	177	176	176	177	176
Marshall Islands	177	176	176	178	178	177	177	178	177
Samoa	184	180	181	183	179	178	179	180	178
Tonga	178	181	183	184	180	179	178	179	179
Andorra	183	177	178	179	182	181	180	181	180
Vanuatu	175	178	179	180	183	182	181	182	181
Congo	176	179	180	182	184	183	182	183	182
Tuvalu	184	186	186	186	186	185	184	184	183
Solomon Islands	184	186	186	186	186	185	184	184	183
Palau	184	186	186	186	186	185	184	184	183
Venezuela (Bolivarian Republic of)	47	47	49	49	49	NA	NA	NA	NA
South Sudan	184	186	177	181	181	180	NA	NA	NA
Eritrea	171	172	NA	NA	NA	NA	NA	NA	NA
Iran (Islamic Republic of)	94	96	98	91	89	80	79	79	NA

**Table 13:** Country ranking in police cooperation against transnational crime (version 2)

Country	2010	2011	2012	2013	2014	2015	2016	2017	2018
United States of America	1	1	1	1	1	1	1	1	1
Japan	5	5	3	2	2	2	2	2	2
Germany	2	2	2	3	3	3	3	3	3
China	3	3	5	5	6	5	4	4	4
United Kingdom of Great Britain and Northern Ireland	4	4	4	4	4	6	5	5	5
Brazil	11	13	13	13	13	9	7	7	6
Italy	6	6	6	6	5	4	6	6	7
India	17	7	7	7	7	7	8	8	8
France	8	8	8	8	8	8	9	9	9
Russian Federation	25	33	34	29	25	11	10	10	10
Egypt	10	10	12	12	12	15	14	11	11
Togo	7	9	9	9	9	10	11	12	12
Mexico	28	22	19	14	14	13	12	13	13
Greece	42	12	11	11	10	12	13	14	14
Sweden	9	11	10	10	11	14	15	15	15
Turkey	29	31	33	34	34	27	26	21	16
Canada	16	17	16	15	15	16	16	16	17
Philippines	14	16	17	18	19	20	19	18	18
Chile	15	18	18	19	18	19	17	17	19
Finland	13	15	15	17	17	18	18	19	20
Belgium	12	14	14	16	16	17	20	20	21
New Zealand	18	19	21	22	20	23	21	22	22
Senegal	19	21	22	23	22	24	22	23	23
Spain	31	29	23	21	23	21	23	24	24
Netherlands	20	20	20	20	21	22	24	25	25
Haiti	57	23	24	24	24	25	25	26	26
Norway	22	24	26	26	26	26	27	27	27
Guatemala	24	26	27	27	27	29	28	28	28
Panama	27	30	31	32	30	31	31	29	29
Luxembourg	41	43	32	33	33	32	32	30	30
Austria	23	25	25	25	28	28	29	32	31
Portugal	30	32	30	30	29	30	30	31	32
Switzerland	26	28	29	31	32	33	33	33	33
Argentina	21	27	28	28	31	35	36	39	34
Indonesia	44	45	45	44	41	38	38	35	35
Honduras	33	35	36	36	35	36	35	34	36
Poland	32	34	35	35	36	34	34	36	37
Australia	35	38	39	39	39	37	37	37	38
Jamaica	36	37	38	38	37	39	39	38	39
Israel	34	36	37	37	38	40	40	40	40
Mauritius	37	39	40	40	40	41	41	41	41
Liberia	38	41	42	43	43	45	44	44	42
Gabon	40	42	43	45	44	42	43	42	43
Lesotho	43	44	44	42	42	43	42	43	44
Republic of Korea	101	93	91	87	95	44	45	47	45
Afghanistan	89	88	92	85	73	73	75	52	46
Madagascar	49	49	48	47	46	46	47	50	47
Sierra Leone	102	99	105	120	56	52	51	49	48
Ghana	96	102	46	50	47	48	48	46	49
Denmark	45	46	47	48	48	49	50	48	50
Democratic Republic of the Congo	39	40	41	41	45	47	46	45	51
South Africa	59	59	59	54	53	50	52	54	52
Bolivia (Plurinational State of)	46	48	50	52	51	51	53	51	53
El Salvador	48	50	51	53	52	53	55	53	54
Sudan	126	124	123	124	101	105	100	94	55
Malawi	68	67	54	46	50	56	49	58	56
Paraguay	50	52	52	56	54	57	56	56	57
Dominican Republic	51	51	53	55	55	58	57	57	58
Ukraine	58	60	63	65	62	55	54	55	59
Costa Rica	52	53	56	59	57	59	58	59	60
Hungary	53	55	55	58	60	60	59	60	61
Rwanda	55	54	57	57	59	61	61	63	62
Nicaragua	54	56	58	60	61	62	63	64	63
Peru	56	57	60	61	63	63	62	61	64
Cote d'Ivoire	123	114	73	76	79	84	84	62	65
Central African Republic	70	68	68	51	58	54	60	68	66
Uganda	66	63	64	63	65	66	65	67	67
Nigeria	73	73	76	78	78	77	70	66	68
Tunisia	61	61	65	66	66	65	66	65	69
Mozambique	67	71	75	73	76	75	64	70	70
Belarus	63	64	67	68	69	67	67	69	71
United Republic of Tanzania	60	58	61	64	64	64	68	72	72
Ecuador	62	62	66	67	67	68	69	71	73
Algeria	74	75	77	77	77	74	73	74	74
Trinidad and Tobago	64	65	69	70	71	70	71	73	75
Cyprus	65	66	70	71	70	69	72	75	76
Pakistan	71	70	72	69	72	76	77	78	77
Cameroon	69	69	71	72	74	71	76	76	78
Zambia	72	72	74	75	75	72	74	77	79
Ethiopia	98	89	62	62	68	78	80	83	80
Morocco	97	76	82	82	82	82	81	80	81
Burkina Faso	75	77	80	80	81	79	78	81	82
Kuwait	79	81	85	88	87	87	86	82	83
Cuba	128	129	132	83	85	86	87	85	84
Saudi Arabia	82	83	87	84	86	85	83	84	85
Mali	77	78	81	81	83	81	85	88	86
Suriname	81	80	84	89	90	89	88	86	87
Burundi	145	141	79	74	84	101	98	102	88
Bulgaria	78	79	83	86	88	88	89	87	89
Uruguay	80	82	86	90	92	90	90	90	90
Guinea	76	74	78	79	80	83	82	89	91
Bahrain	83	84	90	93	94	92	91	91	92
Jordan	84	85	89	92	93	93	92	92	93
Colombia	95	101	103	104	104	96	93	93	94
Timor-Leste	85	86	88	95	96	99	102	99	95
Benin	90	91	94	97	99	94	94	95	96
Kyrgyzstan	86	90	96	98	98	95	97	96	97
Myanmar	88	92	95	94	97	98	99	98	98
Czechia	138	142	142	99	91	91	95	97	99
Malaysia	100	103	102	101	102	102	101	100	100
Kenya	87	87	93	96	100	100	103	103	101
Sao Tome and Principe	91	94	97	100	103	104	104	104	102
Libya	105	97	109	107	106	97	96	101	103
Yemen	125	121	121	126	131	126	111	105	104
Lao People's Democratic Republic	92	95	101	103	105	107	107	107	105
Romania	110	110	111	115	117	110	108	106	106
Niger	109	104	104	102	107	103	105	109	107
Slovakia	93	100	100	105	108	106	106	108	108
Djibouti	99	98	99	106	109	108	109	110	109
Angola	151	154	154	148	120	118	116	118	110
Cabo Verde	108	108	108	110	112	111	112	112	111
Iraq	114	116	119	122	124	117	113	111	112
Azerbaijan	117	118	120	123	125	122	114	114	113
Mongolia	104	109	113	113	115	114	118	115	114
Eswatini	152	153	114	112	114	113	115	117	115
Armenia	107	107	110	111	113	112	117	116	116
Republic of Moldova	103	105	106	109	110	109	110	113	117
Albania	111	111	112	114	116	115	119	119	118
Croatia	118	119	122	125	126	125	125	120	119
Ireland	106	106	107	108	111	116	120	121	120
Namibia	115	115	117	119	121	121	121	122	121
Republic of North Macedonia	112	112	115	118	122	120	124	125	122
Bosnia and Herzegovina	113	113	116	117	119	119	122	124	123
Serbia	116	117	118	121	123	123	123	123	124
Lithuania	121	123	126	129	130	128	127	126	125
Latvia	120	122	125	128	128	127	128	127	126
Seychelles	119	120	124	127	127	129	129	128	127
Barbados	165	164	167	167	129	130	130	129	128
Thailand	139	139	143	116	118	124	126	131	129
Malta	122	125	127	130	132	131	131	130	130
Monaco	124	126	128	131	133	132	132	132	131
Zimbabwe	131	132	136	133	135	133	133	133	132
Lebanon	129	130	133	136	138	136	136	134	133
Viet Nam	146	146	131	135	137	135	135	135	134
Brunei Darussalam	130	131	134	137	139	138	137	137	135
Nepal	136	127	129	132	134	134	134	136	136
United Arab Emirates	132	133	135	138	142	139	139	138	137
Cambodia	127	128	130	134	136	137	138	139	138
Sri Lanka	140	143	145	145	146	141	140	140	139
Mauritania	133	134	137	140	141	140	141	141	140
Singapore	143	145	147	147	149	142	142	142	141
Comoros	135	135	138	141	143	143	143	143	142
Qatar	134	136	139	142	144	145	144	144	143
Gambia	144	138	140	139	140	144	145	145	144
Tajikistan	141	140	144	144	148	147	146	147	145
Guyana	137	137	141	143	145	146	147	146	146
Bahamas	142	144	146	146	147	148	148	148	147
Chad	168	169	163	155	165	152	150	149	148
Papua New Guinea	147	148	151	152	152	150	151	150	149
Guinea-Bissau	148	150	148	149	150	149	149	152	150
Bhutan	149	149	150	151	151	151	152	151	151
Kazakhstan	161	162	164	166	166	162	153	153	152
Georgia	150	151	152	154	155	153	154	154	153
Belize	153	152	153	153	154	154	155	155	154
Oman	159	160	161	163	163	156	156	156	155
Fiji	179	182	182	185	185	184	183	157	156
Turkmenistan	154	155	157	160	160	157	157	158	157
Saint Vincent and the Grenadines	155	156	155	156	156	160	159	159	158
Dominica	181	184	184	157	157	164	163	160	159
Botswana	156	159	158	159	159	159	161	163	160
Slovenia	160	161	160	162	161	163	160	161	161
Montenegro	157	158	156	158	158	158	158	162	162
Nauru	180	183	162	164	164	161	162	164	163
Maldives	174	175	175	173	172	171	164	165	164
Grenada	158	157	159	161	162	165	165	166	165
Antigua and Barbuda	162	163	165	165	167	166	167	167	166
Saint Kitts and Nevis	164	165	166	168	168	168	169	169	167
Estonia	163	166	168	169	169	167	168	168	168
San Marino	166	167	169	170	170	169	170	170	169
Bangladesh	169	147	149	150	153	155	166	172	170
Kiribati	167	168	170	171	171	170	171	171	171
Uzbekistan	170	170	171	172	173	172	172	173	172
Equatorial Guinea	173	173	173	176	176	174	173	174	173
Saint Lucia	182	185	185	174	174	173	174	175	174
Iceland	172	171	172	175	175	175	175	176	175
Micronesia (Federated States of)	175	174	174	177	177	176	176	177	176
Marshall Islands	177	176	176	178	178	177	177	178	177
Samoa	184	180	181	183	179	178	179	180	178
Tonga	178	181	183	184	180	179	178	179	179
Andorra	183	177	178	179	182	181	180	181	180
Vanuatu	175	178	179	180	183	182	181	182	181
Congo	176	179	180	182	184	183	182	183	182
Tuvalu	184	186	186	186	186	185	184	184	183
Palau	184	186	186	186	186	185	184	184	183
Solomon Islands	184	186	186	186	186	185	184	184	183
Iran (Islamic Republic of)	94	96	98	91	89	80	79	79	NA
Venezuela (Bolivarian Republic of)	47	47	49	49	49	NA	NA	NA	NA
Eritrea	171	172	NA	NA	NA	NA	NA	NA	NA
South Sudan	184	186	177	181	181	180	NA	NA	NA

#### Results

According to the results, the Unites States performs excellently in both performance and contribution in police cooperation against transnational crime. It has maintained first place from 2010 to 2018. European countries such as Germany, Britain and Italy also perform well. Japan and China achieve high ranking in Asia. Countries in Latin America with a serious transnational crime problem, such as Brazil, also perform well.

The United States has a long history in combating transnational crime. From America’s first-ever International Crime Control Strategy, announced by the President Clinton in 1998, to the 2011 Strategy to Combat Transnational Organized Crime and the 2017 Presidential Executive Order on Enforcing Federal Law with Respect to Transnational Criminal Organizations and Preventing International Trafficking, the US government has initiated various policies and programs to combat transnational crimes and ensure national security. Some of these policies are designed to investigate, disrupt and dismantle transnational criminal networks, and others facilitate bilateral and multilateral law enforcement cooperation with international organizations and government institutions. Against this backdrop, the Trump administration also intensified previous efforts by “exercising and expanding prosecutions, sanctions and enforcement agencies’ legal authorities; allocating additional investigative resources; and demonstrating the political will to bring TCOs to justice, seize their assets and deny them access to the international financial system.”^51^ Congress is the main institution that formulates and directs strategies and policies regarding transnational crime. Federal agencies including the Departments of Defense, Justice and Homeland Security are responsible for the implementation of these policies, including criminal investigations, coordination, etc.

**Fig. 9:**
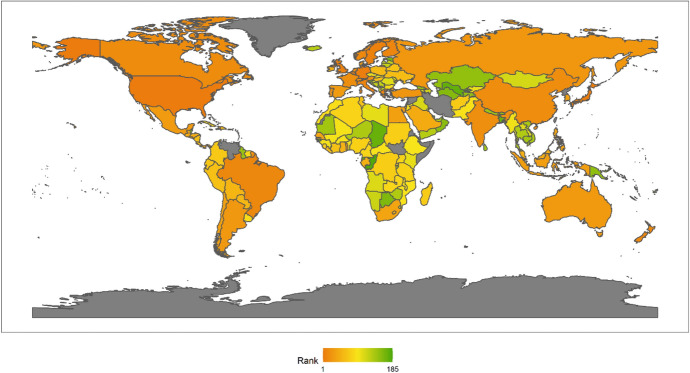
2018 Index ranking of police cooperation against transnational crime on a world map

European countries also have relatively long histories in combating transnational crime. Alongside the collapse of the Soviet Union and the subsequent changes in Russia and the Commonwealth of Independent States, the phenomenon of transnational organized crime has moved from the Eastern Europe to Western Europe and became an apparent threat to many democratic Western countries.^52^ The existence of the EU provides a platform for facilitating bilateral and multilateral cooperation among member states. Regarding legislation, it is possible for the EU to issue continental level legislation such as “Europol Convention”, which aims to improve the “effectiveness and cooperation of the competent authorities in the Member States in preventing and combating terrorism, unlawful drug trafficking and other serious forms of international crime”.^53^Furthermore, the European Police College provides policing officials training and assistance, while the Organization for Security and Co-operation in Europe strengthens policing-related capacity of member nations (Fig. 9).

The top 10 countries in 2018 (according to the first version of the index) were the Unites States, Japan, Germany, Great Britain, Sweden, Belgium, Italy, Greece, Finland and New Zealand. These countries performed well in both performance and contribution. The above map shows the ranking of each country in 2018. Compared with the results for 2017 in our last annual report, little has changed. Countries in North America, South America, Europe and Australia made relatively greater contributions compared with nations in Africa and Southeast Asia. In Africa, Algeria, Libya and South Africa contributed relatively more than did the other countries.

#### Regional Analysis

See Fig. 10.

**Fig. 10:**
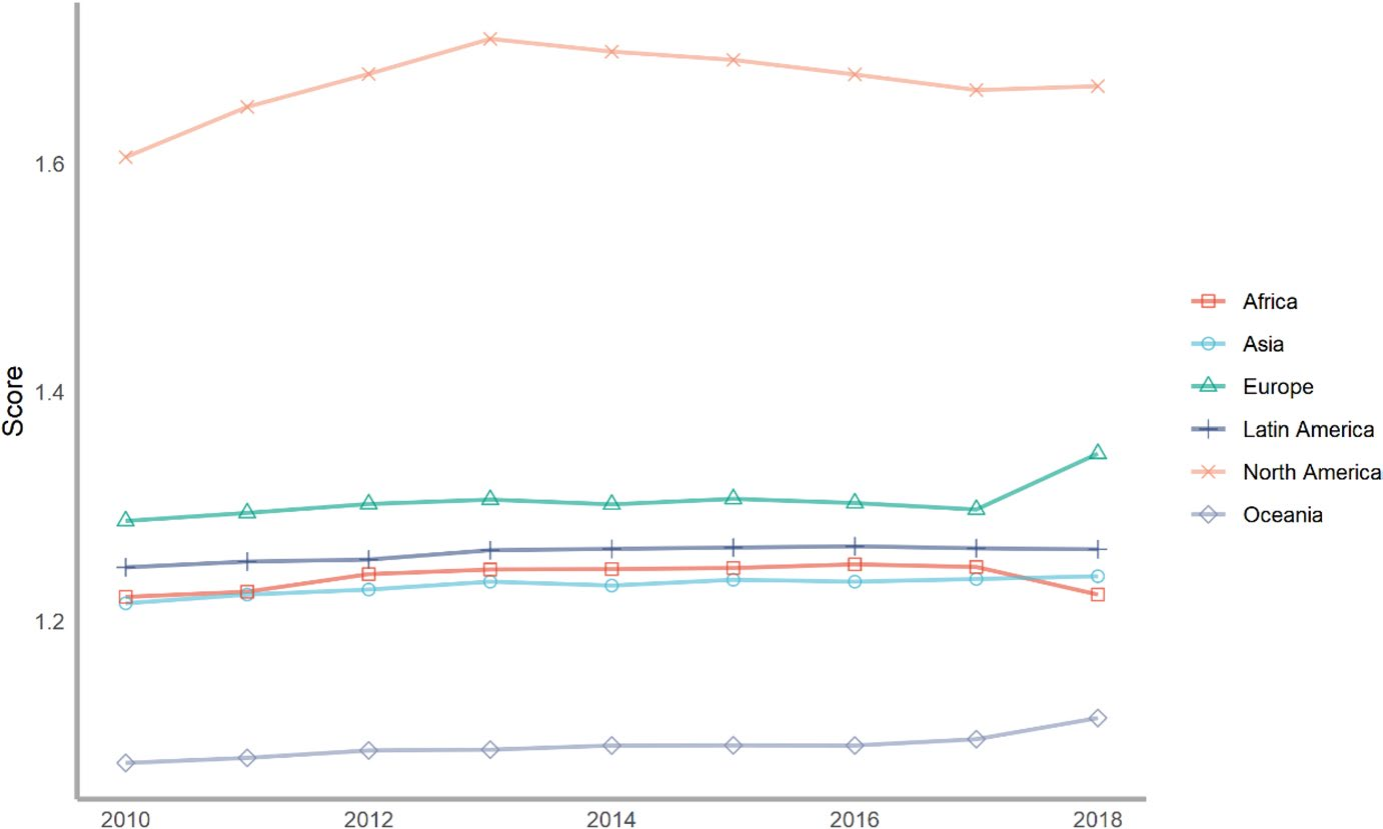
Scores for police cooperation against transnational crimes across continents, 2010–2018

**Asia** Within Asia, Southeast Asia is the region with highly developed transnational organized crime. One reason for this is that border management is relatively weak and cross-border corruption is a serious problem. The four most active transnational organized crime markets in the Southeast Asian region are: drugs and precursor chemicals (methamphetamine and heroin, trafficking in persons and smuggling of migrants, environmental crimes (wildlife and timber trafficking), and counterfeit goods and falsified medicines.^54^ Meanwhile, criminal activities and networks in Southeast Asia have achieved global reach and affected surrounding regions. As a result, regional cooperation is crucial in combating transnational organized crime in Asia, and the Association of Southeast Asia (ASEAN) plays a very important role. ASEAN was established on 8 August 1967 in Bangkok, Thailand. The initial member states included Thailand, Indonesia, Malaysia, Philippines and Singapore. The main purposes of ASEAN are to accelerate economic growth, promote regional peace and active collaboration and provide mutual assistance. Combating transnational crime has been one of its major aims since 1976, but the focus from 1976 to 1997 was limited to the illegal drug trafficking, which led to it having only a weak impact. On 20 December 1997, ASEAN issued the ASEAN Declaration on Transnational Crime, which was the beginning of a series of cooperative activities between governments. After that, combatting money laundering and counter-terrorism were included in the ASEAN agenda against transnational organized crime.^55^

ASEAN has also been in cooperation with countries in other parts of Asia. For example, Japan is a long standing partner of ASEAN. In 2004, ASEAN and Japan issued the ASEAN-Japan Joint Declaration for Cooperation to Combat International Terrorism, and in 2014 they put forward the ASEAN–Japan Joint Declaration for Cooperation to Combat Terrorism and Transnational Crime. Both of the declarations emphasize the importance of strengthening cooperation at bilateral, regional and international levels to combat transnational crime through information exchange and intelligence sharing. Japan has the highest score in Asia in our ranking. Other East Asian countries also performed relatively well and ranked highly under our measurement, including China and Korea. In China, a particular problem relating to organized crime corruption. The Chinese government has facilitated many bilateral and multilateral cooperative efforts with other countries to combat transnational crime, especially corruption, money laundering and drug smuggling and ensure national security. In contrast to many other countries, such as EU member states, informal cooperation is the most prominent type of cooperation in combating transnational crimes in China.^56^

However, there exists research showing that, compared with other regions, the major problem in Asia is that consensus is limited among Asian authorities on what the main organized crime-related problems are. Different Asian countries have different opinions about which specific type of organized crimes should be regarded as the most important. Transnational crime may not be a priority to all of them. “Consistent with this view, Asian authorities do not see much linkage between the local or regional criminal groups about which they are most concerned and transnational organized crime. There is limited collaboration or linkage between transnational organized crime groups and terrorists.”^57^ As a result, reinforcing cooperation and establishing operating networks is still a method through which Asian authorities could improve their capability and efficienscy in combating transnational crimes.

**Europe** The existence of the EU provides a platform for bilateral and multilateral cooperation among member states, which partially explains why European countries performed generally well on this issue. There are a large number of programs, plans and associations regarding combating transnational crimes among EU member states. For example, the Central European Initiative (CEI), the Organization for Security and Cooperation in Europe (OSCE), the European Commission, the European Union Council and its secretariat, the Council of Europe, Europol, the Southeast European Cooperation Initiative (SECI), the Stability Pact and the Adriatic Sea Initiative, Southeast European Law Enforcement Center (SECI) for the Fight against Organized Crime in Bucharest and the Stability Pact initiative on Organized Crime (SPOC).^58^

Compared with Western Europe, the problem of transnational crime is more serious in Southeastern Europe. Transnational organized crime and corruption have been identified by the EU as key problems in Southeastern Europe and obstacles to European integration.^59^ Multiple policies have been issued to attempt to solve the problem. Taking the corruption in customs agencies as an example, states in Southeastern Europe, including Estonia, Hungary, Latvia, Lithuania, Slovenia, Poland, Romania and Bulgaria, have introduced various regulations to establish control mechanisms and improve transparency. More specifically, “Hungary set up a Central Investigation Office in 2000. Latvia introduced the following customs measures: a system of electronic declaration of goods, a more precise delineation of duties and authorities of customs officers, a cooperation scheme with the Border Guard, and staff rotation. Customs departments in Lithuania and Slovenia also undertook a series of reforms.”^60^

**North America** Both the US and Canada performed well in combating transnational organized crimes. According to our ranking, the US maintained first place from 2010 to 2018 on this issue, and Canada also ranked 14th in 2018. Organized crime in North America is not singular phenomenon. In most cases, it is perpetrated by large and small groups which cooperate to make a given crime possible. There are five major categories of transnational criminals that have been most widely discussed: Cosa Nostra groups, Russian groups, Chinese groups, Mexican groups, and Canadian groups.^61^ As discussed above, in respect to nationwide transnational crimes, there have been a large number of policies, programs and initiatives between North American countries and Latin American countries to facilitate cooperative combating of transnational organized crime. Taking cooperation between the US and Mexico as an example, Amy Pope summarized three reasons why successful cooperation has taken place. She compared two human trafficking cases which were successfully investigated and prosecuted in both the US and Mexico. According to her research, the first important factor is commitment from key actors. In the two cases, senior leadership within the Mexican attorney general’s office greatly supported the investigation and regarded it as a priority, which lead to the Mexican prosecutors participating at the working level. Second, mentoring and training is needed. Likewise, in these two cases the US provided training and assistance on law enforcement and other capacities. Third, it is crucial for the two countries to efficiently share formal and informal information and evidence.^62^

**Latin America** Among the various types of organized crimes, the problem of illegal drugs is the most serious transnational crime in Latin America. It is complex and dynamic, and response measures have changed over the years. Additionally, the problem of illegal drugs in Latin America has achieved global reach and played a crucial role in global illicit drug markets. For example, “South America is the sole producer of cocaine for the global market; Mexico and Colombia are the primary sources of opiates in the United States; Mexico and the Caribbean are major foreign sources of cannabis (marijuana) consumed in the United States; and Mexico is the primary source of foreign methamphetamine in the United States.”^63^ As a result, North American countries and as EU countries have worked closely with Mexico, Colombia, and the Caribbean through information exchange and intelligence sharing to establish cooperative frameworks to combat drug trafficking. For example, the US Congress first enacted a chapter in the Foreign Assistance Act in 1971 that mentioned drug control programs in Latin America and the Caribbean. In the mid-1970s, the US has begun to provide training and equipment assistance to drug source countries of Colombia, Bolivia, Peru and Later Mexico to improve counternarcotic law enforcement capabilities.^64^ The Plan Colombia and Plan Mexico (The Merida Initiative in Mexico) are also examples of US efforts to improve the professionalism of police forces and judicial system capacity by providing financial and personnel assistance. The former, which is a security cooperation agreement between the United States, Mexico and other countries in Central America, builds on the experience of the latter, which aimed at combating Colombian drug trafficking. The flourishing of such institutions, programs and plans are not only in the interests of Latin America, but also contribute to the improvement of global justice. Besides direct assistance through policy tools, another way for the US and EU countries to better assist Latin America in combating drug trafficking is by “expanding efforts to prevent, treat, and reduce the harm associated with drug use, while also reprioritizing enforcement efforts.”^65^

**Africa** Countries in Africa stood at the bottom according to our ranking on combating transnational crime. Research by Hatchard analyzed three challenges faced by Africa in combating transnational crime and discussed how African countries can address these challenges. The first challenge is “how to deal with crimes that emanate from outside the jurisdiction”. The second challenge concerns “investigating crimes with a transnational element”. And the third challenge is “tracing and then recovering the proceeds of crime that have been moved out of the country where the crime occurred”.^66^ According to Hatchad, one way to cope with these challenges is by prosecuting the perpetrators. In addition, African countries can take advantage of Interpol, which counts all sub-Saharan African states as members, since “it facilitates cross-border police cooperation, even where diplomatic relations do not exist between the requested and requesting countries”. Finally, there are three other points that are worth taking notice of. First, the need for political will to tackle transnational crime is fundamental. Second, the investigation and prosecution of such cases is often an expensive business and it is entirely justifiable that African states look to other countries to assist with this heavy financial burden. Third, the issue of immunities remains an obstacle to the prosecuting of serving heads of state/government suspected of involvement in transnational criminal activities.

**Oceania** Oceanian countries have a relatively low level of crime prevalence. However, given the pace of globalization, Oceanian countries have also been influenced by global criminal networks and foreign criminal groups, and transnational crime has become increasingly common. The most significant category of transnational organized crime in the Oceanian region is the illicit drug industry. Indeed, Oceania has the highest rate of use of amphetamine-type stimulants and cannabis in the world.^67^ As a result, it is also important for Oceanian countries to participate into the global network against transnational crime in order to obtain access to information and intelligence. There have been bilateral legal and cooperative initiatives between Australia and regional states, such as China, Malaysia, Thailand, Vietnam and Indonesia. The cooperation can improve the capacity of Australia to prevent and combat criminal activities and ensure the safety and security of the state.

#### Conclusion

Transnational organized crime threatens the interests, stability and national security of every state, which makes combating transnational crime an important part of improving global justice. In this section, we measure the performance and contribution of each nation state in combating transnational crime with 14 indicators in all. Nine of the indicators relate to the ratification status of each country of relevant treaties, while the remainder of the indicators belong to the category of contribution, which measures donations to Interpol, donations to UNODC and FATF membership. According to our results, the Unites States has ranked at the top from 2010 to 2018. European countries also ranked highly according to our measurement. In Asia, Japan and China performed well. Generally speaking, countries in North America, South America, Europe and Australia made relatively greater contributions compared with nations in Africa and Southeast Asia.

### Issue 6: Refugees

#### Introduction

The world has witnessed an unprecedented level of human mobility over decades. Millions of people around the world have moved to escape armed conflicts, terrorism, persecution, poverty, food insecurity, environmental disasters and other life challenges. These people are usually treated as “refugees”. According to the UN refugee agency, refugees are “people who have fled war, violence, conflict or persecution and have crossed an international border to find safety in another country.”^68^

Today, the refugee crisis is one of the most complex issues in the world: it not only has a range of economic, social, political and environmental impacts on both the home and host countries,^69^ but has also posted great challenges to global justice. Therefore, we include refugee issue as one of the key issue areas in this year’s global justice index, to improve the measurement of developments in global justice.

The most recent UN figures report that forcibly displaced people are estimated at 79.5 million worldwide by the end of 2019, representing about 1% of the world’s population. Of these, 45.7 million are internally displaced persons, and 25.9 million are refugees who have been forced to flee their countries, around half of whom are under the age of 18. Statistics from 2010 to 2018 further indicate that the volume of refugee movements across international borders has more than doubled and has reached a thirty-year high.^70^ During 2018 alone, 1.1 million people were recognized as new refugees. The refugee crisis is a global challenge that individual states cannot address alone. “Refugee crises call for a global sharing of responsibility,” said Filippo Grandi, the head of the United Nations High Commissioner for Refugees (UNHCR). As of December 2019, 146 and 147 UN member states have, respectively, ratified the key international legal framework for the protection of refugees, the 1951 Convention relating to the Status of Refugees and its updating Protocol adopted in 1967. Today, as refugee numbers continue to grow, the need for more comprehensive, more coherent and more coordinated policies to manage refugee flows and protect the rights and well-being of forced migrants is greater than ever.

As a truly “whole-of-international community affair”, the governance of the refugee problem needs to adopt the principle of “Common but Differentiated and Respective Capabilities (CBDR-RC)” proposed by our project. Achieving global justice by safeguarding the rights and well-being of refugees and their families^71^not only requires cooperation and dialogue between and among countries, but also calls for actions and contributions by individual countries,^72^ including both countries of origin and countries of destination. Efforts by individual countries to combat the refugee crisis are a significant aspect of the global justice agenda. This sub-index is designed and constructed to rank individual countries according to their level of performance in and contribution to global justice in the field of refugee governance.

#### Dimensions and Indicators

The refugee issue is a multidimensional. It is highly challenging, if not impossible, to construct a sub-index to rank and compare individual states in managing the refugee crisis. As a first attempt, we try to use two categories, performance and contribution, to measure individual countries’ influence on global justice in the issue area of refugee governance. For the category of performance, we use the size of the exported refugee population per 1000 inhabitants to measure a country’s performance in reducing and governing refugees. For the category of contribution, we use five dimensions to measure each country’s investments and efforts toward global refugee governance. These five dimensions are as follows: (1) the number of imported refugees divided by the natural logarithm of GDP^73^; (2) implementation of RSD (refugee status determination), measured by the number of decisions made and proportion of positive decisions made; (3) participation in international refugee governance measured by membership of UNHCR and the signing of international agreements, including the 1951 Convention relating to the Status of Refugees and its 1967 Protocol; (4) national policies on managing refugee issues, including indicators such as systems for receiving, processing and identifying refugees, planning for displaced populations, specific measures to provide assistance, disaster risk reduction strategies and granting of permission for temporary stay or temporary protection; (5) standard of living for refugees, measured by type of refugee accommodation provided. Data are obtained from the World Bank, UNHCR Statistical Yearbook, UNHCR-Annex of Global Appeal, and UN Report of World Population Policies respectively (see Table 14). Relying on these multidimensional data and the measurement strategies, we try to present a more comprehensive picture of global justice in the domain of refugee governance.

**Table 14:** Data on refugees

Category	Dimension	Indicators	Data Source	Coverage
Performance	Refugee population	Exported refugee population to per 1,000 inhabitants	World Bank; UNHCR Statistical Yearbook	187 countries (2010–2018)
Contribution	Refugee population	Imported refugees to per log (GDP)	World Bank; UNHCR Statistical Yearbook	211 countries (2010–2018)
	Implement of Refugee Status Determination	Number of decisions made Proportion of positive decisions	UNHCR Statistical Yearbook	169 countries (2010–2018)
	Participation in international refugee governance	Membership of UNHCR Signing international agreements	UNHCR-Annex of Global Appeal	Membership: 157 countries (2019) Agreements: 157 countries (1951–2019)
	National policies on refugee issues	Systems for receiving, processing and identifying refugee; planning for displaced populations; Specific measures to provide assistance; Disaster risk reduction strategies; Granting of permission for temporary stay or temporary protection	World Population Policies	102 countries 99 countries 96 countries 92 countries 101 countries (2019)
	Standard of living	Type of refugee accommodation	UNHCR Statistical Yearbook	122 countries (2010–2018)

#### Results

Using the index construction methods developed by this project (see the methodology section), this sub-index ranks 192 countries from 2010 to 2018 according to their level of performance in and contribution to global justice in the issue area of refugee governance (see Table 15).

**Table 15:** Country ranking in refugee aspect of promoting global justice

Country	2010	2011	2012	2013	2014	2015	2016	2017	2018
Sweden	3	2	1	1	1	1	1	3	1
France	1	1	2	2	2	3	4	8	2
Germany	9	8	8	8	11	5	3	7	3
Spain	6	4	4	4	3	2	2	5	4
United Kingdom of Great Britain and Northern Ireland	2	3	3	3	4	4	5	9	5
Switzerland	8	9	11	10	8	9	7	4	6
Finland	7	7	5	5	5	7	6	2	7
Canada	5	6	7	6	6	6	9	11	8
Ireland	10	10	10	11	12	12	13	6	9
Belgium	25	17	18	17	18	14	12	12	10
Norway	4	5	6	9	9	8	8	1	11
Denmark	12	14	13	15	15	11	10	14	12
Argentina	19	19	15	18	17	15	17	17	13
Italy	11	11	9	7	7	10	14	15	14
Philippines	20	20	16	16	14	16	18	18	15
Mozambique	14	21	21	12	16	17	19	19	16
Brazil	32	13	12	13	10	19	15	16	17
Austria	13	12	14	14	13	13	16	10	18
Zambia	16	24	17	21	20	20	22	22	19
Greece	22	16	20	23	22	21	23	26	20
Portugal	34	32	25	28	24	25	26	23	21
Japan	43	38	29	20	21	22	21	29	22
Uruguay	45	41	40	33	32	29	24	24	23
Thailand	24	31	24	19	19	23	20	28	24
Australia	27	23	23	22	23	24	27	27	25
United States of America	28	26	35	35	41	43	30	33	26
Paraguay	38	40	37	44	25	27	31	32	27
Malta	31	36	38	29	39	18	11	13	28
Luxembourg	15	18	19	27	31	33	33	20	29
Samoa	29	27	26	30	27	30	32	21	30
Netherlands	23	22	27	25	26	26	28	30	31
Lesotho	37	37	31	36	36	49	34	31	32
United Republic of Tanzania	21	35	33	24	30	28	25	25	33
Republic of Korea	35	42	42	45	34	38	38	42	34
Malawi	26	34	36	38	35	31	40	43	35
New Zealand	30	28	28	31	28	32	35	40	36
Lithuania	64	73	74	50	46	45	37	35	37
South Africa	18	25	22	26	29	35	29	44	38
Peru	82	81	76	76	55	50	41	41	39
Slovenia	33	33	34	34	37	41	46	37	40
Chile	53	44	54	40	40	37	44	45	41
Czechia	39	29	30	37	43	42	45	48	42
Romania	77	67	66	54	49	46	42	46	43
Latvia	87	85	88	66	63	66	53	47	44
Kenya	54	59	59	60	64	57	62	59	45
Benin	85	46	49	47	44	44	48	51	46
Jordan	63	70	72	59	50	53	54	57	47
Costa Rica	98	57	55	71	59	61	52	55	48
Israel	65	80	78	81	53	67	55	58	49
India	48	49	45	41	42	48	50	53	50
Qatar	60	47	69	32	33	34	39	39	51
Mexico	55	51	60	52	75	72	59	63	52
Poland	52	50	51	53	56	60	63	61	53
Uganda	47	45	39	43	38	36	43	36	54
Turkmenistan	88	79	75	69	65	65	64	60	55
Madagascar	58	66	62	49	47	51	56	66	56
Malaysia	42	43	48	51	51	55	57	65	57
Bangladesh	44	54	43	39	54	56	51	52	58
Russian Federation	135	108	118	97	60	63	69	72	59
Niger	71	63	52	48	45	39	36	50	60
Papua New Guinea	46	58	44	74	70	58	71	64	61
Iceland	75	68	67	62	52	40	47	49	62
Botswana	40	48	56	80	86	95	91	82	63
China	68	74	65	82	84	82	85	73	64
Algeria	94	90	108	83	77	74	75	74	65
Timor-Leste	51	55	57	65	68	73	73	70	66
Marshall Islands	17	15	46	56	57	59	61	78	67
Cyprus	78	76	80	85	74	62	60	54	68
Estonia	66	72	86	89	81	78	70	69	69
Belarus	106	109	109	99	128	84	78	80	70
Tajikistan	56	60	58	61	69	70	68	83	71
Ghana	110	131	101	120	103	104	96	89	72
Gabon	57	65	61	58	66	54	65	68	73
Morocco	80	84	81	68	72	69	72	76	74
Kazakhstan	83	97	83	78	82	75	76	84	75
Sierra Leone	144	128	99	102	98	101	82	86	76
Egypt	61	64	70	70	78	76	86	87	77
Panama	59	53	47	46	48	47	49	56	78
Chad	132	124	123	131	135	107	106	122	79
Vanuatu	62	77	53	84	85	81	94	62	80
Ecuador	67	69	77	79	80	85	84	94	81
Singapore	79	86	79	86	83	80	80	91	82
Bulgaria	115	127	95	103	110	111	88	97	83
Turkey	119	118	121	98	96	79	77	79	84
Angola	155	145	110	91	92	77	83	77	85
Oman	72	78	73	57	58	71	66	67	86
Tunisia	101	91	93	88	87	88	90	98	87
Suriname	95	87	84	92	95	89	98	85	88
Indonesia	109	106	96	93	91	83	81	92	89
United Arab Emirates	123	88	85	63	62	68	67	88	90
Solomon Islands	91	83	104	105	88	86	87	81	91
Monaco	84	93	97	90	94	91	92	38	92
Burkina Faso	70	56	64	42	67	52	58	71	93
Eswatini	74	71	71	87	116	113	95	95	94
Saudi Arabia	81	82	87	75	73	96	79	90	95
Nepal	96	98	90	107	104	100	99	99	96
Slovakia	50	61	50	67	61	64	74	93	97
Trinidad and Tobago	114	130	122	111	124	121	112	105	98
Bolivia (Plurinational State of)	76	75	68	73	89	97	89	102	99
Republic of North Macedonia	125	134	131	101	100	106	103	101	100
Nigeria	49	52	41	55	79	102	104	103	101
Togo	141	111	113	125	99	103	109	114	102
Fiji	128	116	105	132	101	125	115	109	103
Palau	36	39	32	77	76	94	93	34	104
Kuwait	137	138	130	124	107	105	102	118	105
Senegal	118	99	98	109	102	93	100	107	106
Cambodia	121	117	114	123	117	112	105	104	107
Kyrgyzstan	131	104	111	113	106	109	111	112	108
Ethiopia	97	100	91	96	109	99	101	100	109
Nicaragua	120	121	117	94	93	87	107	108	110
Cote d'Ivoire	143	141	138	118	115	119	130	106	111
Hungary	105	95	94	108	112	108	133	130	112
Cameroon	113	94	124	121	97	90	97	96	113
Belize	93	101	103	104	111	92	126	126	114
Republic of Moldova	166	147	134	110	105	110	116	116	115
Uzbekistan	146	150	144	135	137	133	128	123	116
Mauritius	89	89	100	115	118	122	134	111	117
Liberia	162	146	146	138	136	138	125	134	118
Congo	142	142	142	137	120	134	114	115	119
Azerbaijan	108	113	119	117	121	114	113	120	120
Georgia	159	125	125	129	129	115	124	131	121
Armenia	150	136	127	122	123	120	123	119	122
Guatemala	127	92	82	95	90	98	108	110	123
Guinea-Bissau	107	120	106	127	127	130	118	117	124
Dominica	133	135	136	140	133	128	120	121	125
Lebanon	177	153	152	126	119	117	131	136	126
Pakistan	130	102	92	100	153	151	127	138	127
Jamaica	90	96	116	112	131	129	121	133	128
Montenegro	136	144	143	119	122	131	132	139	129
Dominican Republic	86	105	115	116	126	123	110	135	130
Serbia	145	143	150	141	142	139	137	142	131
Yemen	73	62	63	72	71	116	117	113	132
Namibia	140	152	148	139	141	135	143	141	133
Guyana	165	177	174	174	179	171	158	143	134
Zimbabwe	148	155	149	144	144	136	129	132	135
Brunei Darussalam	124	123	132	133	134	124	138	126	136
Equatorial Guinea	161	162	156	154	154	145	145	144	137
Djibouti	99	103	89	106	125	126	136	146	138
Venezuela (Bolivarian Republic of)	102	107	102	114	108	118	119	137	139
Kiribati	182	181	181	179	151	147	146	126	140
Grenada	170	154	157	155	156	148	135	140	141
Somalia	151	164	158	161	160	159	150	151	142
Ukraine	117	119	120	64	149	156	151	152	143
Guinea	134	139	137	146	143	141	148	148	144
Sao Tome and Principe	152	149	153	151	148	143	149	149	145
Tonga	122	129	128	142	147	144	147	154	146
Bahrain	100	112	135	143	138	146	139	155	147
Gambia	103	132	112	134	113	137	122	145	148
El Salvador	129	140	129	148	146	140	144	153	149
Colombia	154	158	160	160	163	164	160	158	150
Cabo Verde	157	159	159	158	161	165	152	126	151
Tuvalu	69	137	139	153	157	157	155	75	152
Bahamas	92	160	147	157	145	153	157	168	153
Democratic People's Republic of Korea	153	151	155	156	159	163	164	161	154
Albania	149	157	151	147	152	150	153	160	155
Saint Kitts and Nevis	111	114	141	150	140	154	173	169	156
Mauritania	147	156	166	163	158	162	161	164	157
Mali	104	110	175	170	171	176	169	166	158
Andorra	167	166	167	164	170	167	154	126	159
Iran (Islamic Republic of)	116	133	107	130	130	127	140	147	160
Seychelles	176	170	171	176	173	170	141	150	161
Cuba	163	165	164	166	165	161	159	163	162
Mongolia	164	169	162	162	162	160	163	157	163
Rwanda	171	168	161	167	169	168	175	176	164
Democratic Republic of the Congo	175	176	163	159	166	152	165	167	165
Antigua and Barbuda	139	115	133	149	139	132	142	162	166
San Marino	160	148	154	152	155	155	166	126	167
Honduras	112	122	126	136	114	142	156	156	168
Croatia	172	174	173	175	178	175	172	171	169
Barbados	126	126	140	145	150	149	168	165	170
Libya	138	161	145	128	132	158	167	159	171
Bosnia and Herzegovina	181	183	184	168	168	169	170	170	172
Iraq	180	172	178	169	164	166	162	173	173
Central African Republic	174	178	176	178	180	179	178	174	174
Maldives	158	163	165	165	167	172	174	172	175
Afghanistan	173	175	172	177	172	174	179	177	176
Burundi	169	171	169	171	175	180	176	179	177
Sudan	156	167	168	172	176	173	171	175	178
South Sudan	41	30	170	173	177	178	182	184	179
Sri Lanka	183	184	179	181	181	181	177	178	180
Vietnam	168	173	182	182	184	184	184	181	181
Saint Vincent and the Grenadines	178	179	180	183	183	182	180	180	182
Nauru	191	188	190	190	174	177	185	183	183
Saint Lucia	179	180	177	180	182	183	181	185	184
Eritrea	187	190	183	184	185	185	183	182	185
Haiti	188	187	188	188	187	189	187	189	186
Comoros	184	185	185	186	189	188	188	186	187
Micronesia (Federated States of)	186	186	186	185	188	186	186	187	188
Lao People's Democratic Republic	189	189	187	189	190	190	190	190	189
Syrian Arab Republic	185	182	189	187	186	187	189	188	190
Bhutan	191	192	192	192	192	192	192	191	191
Myanmar	190	191	191	191	191	191	191	192	192

The total number of refugees in the world has been rising over the past ten years, with the number almost doubling from 2010 to 2018. The year 2018 brought many challenges to global refugee governance, including the crises in Bangladesh (Rohingya refugees), Venezuela, Syria and Yemen, as well as conflicts in the Democratic Republic of the Congo and the Central African Republic, which have forced a growing number of destitute and vulnerable people to flee their home countries and take refuge beyond their borders. Regarding the geographic distribution of the refugees' countries of origin, the three regions that generated the most refugees were West Asia, East Africa, and Southeast Asia. West Asia (the Middle East) exported 7.27 million refugees in 2019, East Africa exported 4.5 million, and Southeast Asia exported 3.1 million. Meanwhile, the three regions that hosted the most refugees are West Asia, Southeast Asia, and East Africa. There were nearly 5.8 million refugees that moved to West Asia in 2019, 3.54 million in Southeast Asia, and 3.42 million in East Africa. Therefore, it is easy to identify that most refugee flows were actually between countries in the same region, rather than across regions. For example, in 2019, 76% of all refugees from West Asia flowed into neighboring countries in West Asia, and 80% of refugees from South Asia flowed into other countries in South Asia.

**Fig. 11:**
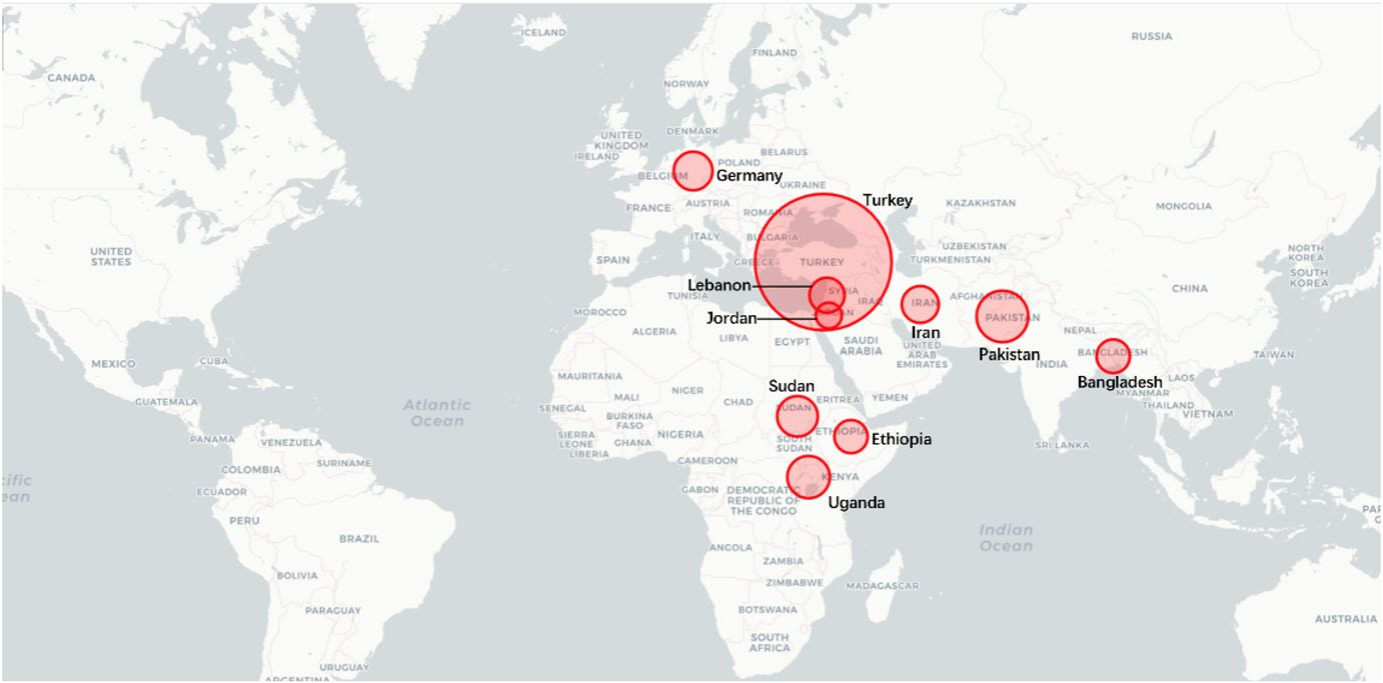
Refugee Host Countries

In 2018, the countries that exported the most refugees worldwide were Syria, Afghanistan, South Sudan, Myanmar, and Somalia. The first four countries exported more than 1 million refugees, of which Syria exported 6.65 million refugees (accounting for 33% of all refugees), which is nearly 2.5 times the second place origin country of Afghanistan (2.68 million). In 2018, the countries that received the most refugees in the world were Turkey, Pakistan, Uganda, Sudan and Germany. Turkey leads the world, and has received nearly 3.68 million refugees (45 refugees per 1,000 citizens. As a share of the population, Lebanon hosts by far the most with 156 refugees per 1000 citizens. Jordan came second in 2018, receiving 72 per 1,000 of its national population.^74^ In recent years, the flow of refugees toward the European Union generated by the prolonged conflict in Syria has captured headlines across the world, but they are in fact only part of a much broader story. For the past decade, most refugees actually cannot afford to travel to these developed countries, rather they have no choice but to flee into neighboring poorer countries. The largest refugee host countries are the developing countries next to the countries of origin (see Fig. 11). The Norwegian Refugee Council has noted that since 2018 “borders have closed for families seeking protection, refugee quotas slashed, and poor host countries left with little international support.”^75^ The situation for these developing countries hosting refugees is worrying, but they have not attracted sufficient attention in the media and academia.

**Fig. 12:**
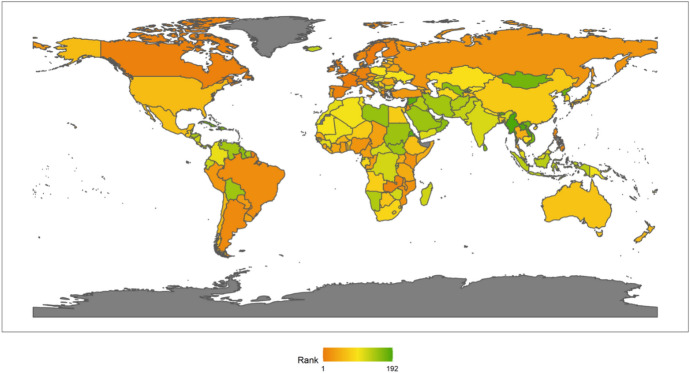
2018 Index ranking of refugee governance on a world map

The intensified refugee crisis is a global issue that calls for common participation and differentiated contribution by individual countries. Over the past decade, varying degrees of progress have been made by individual countries in governing the refugee issue. Among the ten countries ranked top in 2018 (see Fig. 12), nine are in Europe (including Sweden, France, Germany, Spain, the UK, Switzerland, Finland, Ireland, and Slovenia) and one belongs to North America (Canada). The world map below shows the ranking of all observed countries in 2018.

Sweden ranked top in 2018 according to its performance and contribution to refugee governance, a position it has held since 2012 (with the exception of 2017). Sweden has taken refugees for decades. The number of refugees seeking shelter in Sweden has increased over the past 10 years. During 2015 Sweden accepted more refugees per capita than any other country in the EU (163,000 people, almost 2% of the total population).^76^ This is followed by other developed countries, including France, Germany, Spain, the United Kingdom, Switzerland, Finland, Canada, Ireland and Belgium. These traditional resettlement states have expanded the numbers of refugees they host and put forward policy measures to protect refugees’ rights and ensure their access to basic and essential goods. However, there is a mixed trend with regard to refugee policies in these countries. On the one hand, these refugee-hosting countries have overall moved considerably towards more comprehensive policies to save lives and protect newcomers, and many have recognized the potential economic and social contributions refugees can make. Some host countries have even undertaken dedicated measures to facilitate refugee integration, minimize disparities between refugees and nationals, and help build a future for refugee families. For example, Sweden tries to project a strong welfare state by offering refugees publicly funded integration programs which help the newcomers get Swedish language lessons and learn about the culture.^77^ On the other hand, more restrictive legal approaches have been introduced and implemented in recent years in these countries to block influx of outsiders as the politics around refugee issues became more polarized in these places. Many nationalist, far-right and anti-immigrant political parties have surged and tried to reshape state policies across Europe.^78^ For example, fueled by the refugee crisis since 2015, politicians and parties on the radical right in France, Netherlands and Germany have experienced renewed vigor, pushing for policies to fight “an invasion of foreigners.” As a result, the past few years have recorded a deterioration in the situation of refugees in some European countries.

Refugee governance and state stability are linked together.^79^ As of mid-2020, more than two-thirds of the world’s refugees come from five countries experiencing civil wars and fragile governments: Syria (6.6 million), Venezuela (3.7 million), Afghanistan (2.7 million), South Sudan (2.3 million) and Myanmar (1.0 million).^80^ Among the states ranked bottom in 2018, the number of people displaced by conflicts reached a record high in Myanmar, Syria, South Sudan, Afghanistan and Central Africa. Ongoing conflicts, weak states, terrorist attacks, rebels, as well as religious and ethnic fragmentation continued to uproot millions of people, forcing them to leave their homeland. For example, Myanmar is a religiously and ethnically diverse country that has experienced a wide range of conflicts and violence during recent decades. These conflicts have produced a growing number of refugees fleeing across borders. As result of the conflicts that erupted in Myanmar’s Rakhine state in August 2017, approximately 720 000 Rohingya refugees, nearly 80% of whom are women and children, have been forced to flee their homes to escape targeted violence and religious repression, causing a humanitarian crisis on a catastrophic scale. In August 2018, the UN Secretary-General António Guterres emphasized that Myanmar’s refugee problem has become “one of the world’s worst humanitarian and human rights crises”.^81^ In Syria, the country has been caught up in prolonged civil war since March 2011. The increased violence and weak state governance have forced a large number of Syrian citizens to flee for refuge, creating the largest refugee population in the world. In 2018, an estimated more than 6.6 million Syrians were on the run after fleeing the conflict, with 5.5 million refugees living in countries bordering Syria, including Turkey, Lebanon, Joran, Iraq, and Egypt.^82^ Around half of all registered Syrian refugees are actually under the age of 18. UNHCR High Commissioner Filippo Grandi called Syria "the biggest humanitarian and refugee crisis of our time, a continuing cause for suffering." Even worse, with no end in sight, a vicious circle remains with respect to refugee issues and regional conflict. The refugee crisis will further exacerbate concerns about regional destabilization in the Middle East, Southeast Asia and East Africa.

#### Regional Analysis

**Fig. 13:**
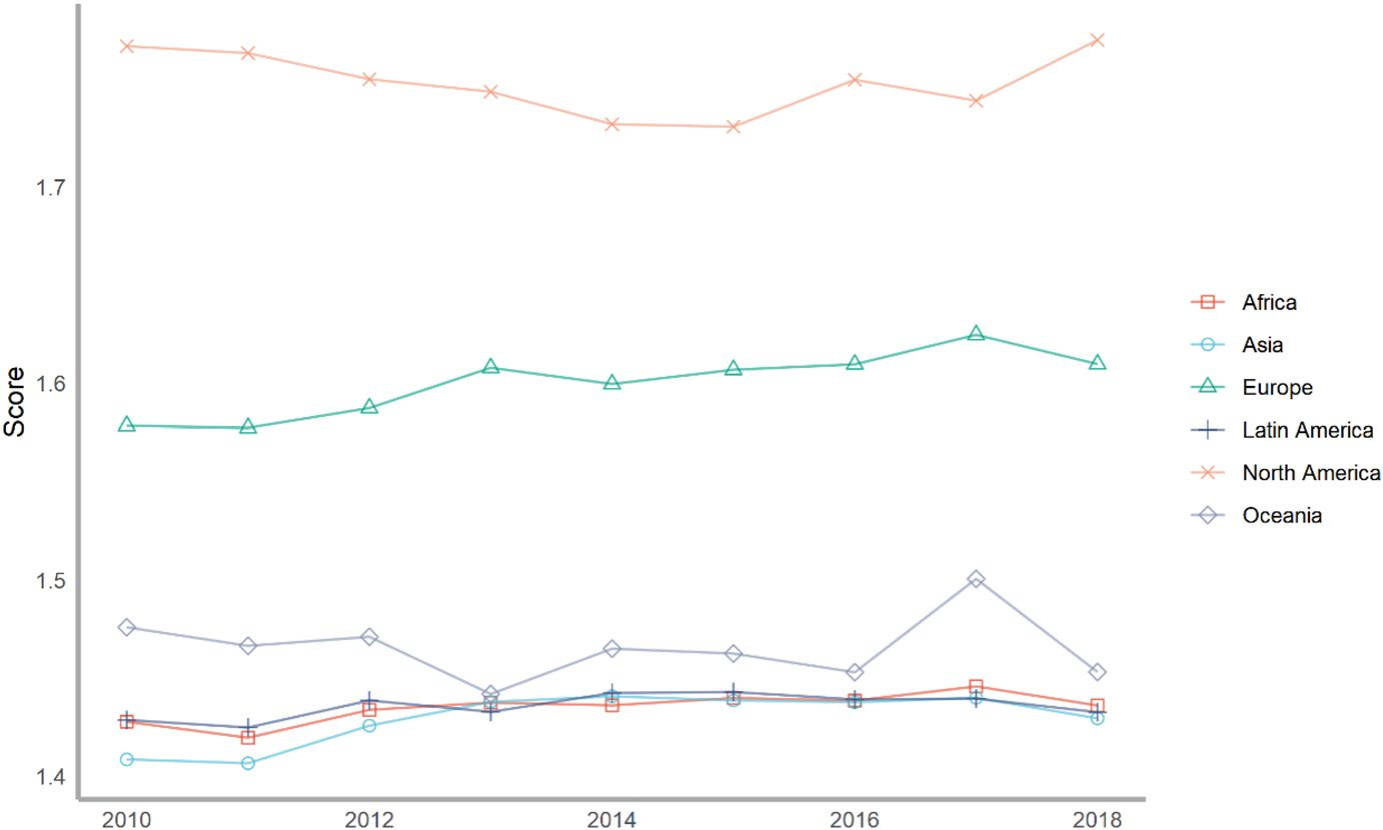
The score of refugee governance issue across continents, 2010–2018

As for regional performance, North America was still ahead in average ranking in 2018, followed by Europe, Oceania, Latin America, Asia and Africa. Nonetheless, the latter four had little difference between them, with the average index scores being at 1.45 and average rankings being in the early 100 s. As shown from Fig. 13, only one of the six regions in the world improved in the global justice of refugee governance in 2018.

**Asia** Asia is a region with large refugee flows (especially in West Asia and Southeast Asia) but limited legal protection. It has done a great job in the RSD Positive Ratio but was scored low in the RSD Number and International Agreement Participation. Less than half of the countries in this region have acceded to the 1951 Convention and its 1967 Protocol. In the past decade Asia's average ranking has risen slightly, mainly driven by particular improvement in Asia's Refugee Housing Situation. As a large region of tremendous economic, political and social diversity, refugee governance varied substantially between Asian countries. Five countries in Asia continue to rank in the top 50 of the sub-index: The Philippines, Japan, Thailand, South Korea and Jordan. The Philippines ranks first in the region and 15th globally in 2018. It scored highly in International Agreement Participation, Refugee Policy, and RSD Positive Ratio, despite its low level of refugee hosting. Japan has risen from 43 in 2010 to 22 in 2018, mainly due to the increase in RSDs and a significant improvement in refugees' accommodation situation. Thailand has performed very well in hosting external refugees and developing refugee policies, but did not fully engaged in international agreements and provided less sufficient accommodations for refugees. Turkey continues to be the most generous host state in the world, but it also generated a large number of refugees fleeing from the country and its RSD Positive Rate was kept very low. Jordan represents a similar case: it ranks second in the world in hosting refugees (most are Syrian) but this is offset by its large magnitude of refugee production and its conservatism in refugee policies. Political conflicts and civil wars have been at the root of the largest refugee outflows in the region. Suffering from unresolved conflicts and insecurity, Afghanistan, Syria and Myanmar remained the three largest refugee origin countries of concern to the UN in Asia. There were about 2.2 million Afghan refugees worldwide in 2018, approximately 95% of whom moved to neighboring Pakistan and Iran.^83^ Myanmar was ranked last in the region and in the global ranking, owing to the Rohingya refugee crisis as well as its poor performance in other respects.

**Europe** Europe's performance was strong, especially in the two indicators of RSD Number and International Agreement Participation, and it was also at the forefront of other indicators. Over the past 9 years, the average ranking of Europe has slightly improved, from 67 to 61. This rise was mainly attributed to its decline in exported refugees and increase in international agreement participation. Since the Treaty of Lisbon entered into force in 2009, Europe has developed a comprehensive institutional framework and burden-sharing mechanisms to protect refugees.^84^ While Europe’s overall efforts toward promoting global justice in the field of refugee governance can be appreciated, gaps in national refugee governance persist. At the national level, Western Europe occupied 9 of the 10 highest ranking countries globally. Countries like Sweden, France, Germany, Spain, the United Kingdom and Finland have been very stable in the rankings, and are almost always in the top 10. In comparison, Eastern European countries, such as Hungary, Croatia, Slovakia and Bulgaria, ranked relatively low. As the refugee crisis in Europe continued throughout 2018, Eastern European countries always bear the brunt. However, these countries either have very limited institutions and resources to accommodate the high arrival of refugees or have simply adopted restrictive policies to stop newcomers at the borders. For example, in 2018, Hungary closed its borders to nearly all the refugees as Prime Minister Viktor Orban and his ruling Fidesz Party adopted an anti-refugee policy (“zero refugee” policy).^85^

**North America** North America was leading the world and maintained its ranking of around 20th from 2010 to 2018, with a slight overall improvement in score of 0.04. Both Canada and the United States have performed well and steadily, although there is a small disparity in refugee governance between the two countries in the North America. Canada has maintained a ranking of around 10th in the past 9 years, and the United States has held its position at around 30th. Canada has done very well in RSD Number and exporting few refugees, ranking in the top 20th in the world. It has also done well in Receiving Refugees, RSD Positive Ratio, Domestic Refugee Policy, and Housing Situation, ranking around 25th to 50th. A relatively lower-scoring aspect was its participation in the international convention, which ranked around the middle globally. Over the past 9 years, Canada's score in Receiving Refugees has dropped by 0.06, and its score in RSD Number has dropped significantly by 0.33, but the RSD Positive Ratio experienced an improvement in score of more than 0.1. The United States has done very well in the three aspects of Receiving Refugees, Exporting Refugees, and RSD Number, which were all ranked in the top 20th in the world. Besides, it ranked in the middle of the world in RSD Positive Ratio and Housing Situation. However, the United States participated in relatively few international conventions, which has dragged down its whole score. In the past few years, it has declined in Receiving Refugees.

**Latin America** Latin America performed relatively poorly, with a sub-index average score of 1.143 in 2018, a score which dropped by 0.1 over the past 4 years, mainly due to the decline in RSDs. The continent performed moderately in areas like Housing Situation, but was falling far behind in areas like National Policies and RSD Positive Ratio. Within the region, there was an obvious performance disparity among the different sub-regions. South American countries such as Argentina, Brazil, Uruguay, Paraguay, Peru, Chile were performing relatively better than Central America and much better than the Caribbean countries. For example, Argentina was ranked 13th globally in 2018, while Mexico was 52nd and Haiti ranked at the bottom, 186th in 2018. The main drivers of displacement in the Caribbean region included natural disasters, gang violence, persistent poverty and fragile states. Natural disasters especially severe storms caused by the effects of global climate change constituted a major cause refugee movement in the Caribbean. For instance, in Haiti, the earthquake of 2010 killed more than 310,000 Haitians and displaced at least 1.5 million. Meanwhile, years of hurricanes, floods and mudslides coupled with weak state governance further exacerbated the refugee crisis in Haiti. An exception in South America is Venezuela which is ranked 139th in the world. Venezuela has been stuck in political and economic crisis for several years, which has produced a large number of refugees, with most travelling towards the Caribbean and other South American countries. After the 2018 election, the situation got even worse, which forced 350 000 new refugees to flee Venezuela in 2018 alone due to violence, insecurity, extreme levels of unemployment and shortages of daily necessities and medication.^86^

**Africa** Africa also scored low, with an average ranking of 108 in 2018, a performance which has been very stable over the past decade. Africa has done very well in terms of Receiving Refugees and RSD Positive Ratio. However, it is also the continent with the most severe export of refugees and the lowest score for Refugee Accommodation Situation. There are some notable intra-regional variations with regard to refugee governance in Africa. The refugee situations in Central Africa and East Africa continued to deteriorate due to widespread violence and conflicts between various armed groups, coupled with other factors including famine, drought, poverty, public health crises and failed states. The Central African Republic, the Democratic Republic of the Congo, Libya, Somalia, Burundi and South Sudan exported the largest number of refugees in the region because of their severe humanitarian situations. In the Central African Republic alone, at least 568 000 refugees had fled to neighboring countries as of mid-2018. The instability of South Sudan created one of the most protracted displacement situations in the world, forcing an estimated 2.5 million refugees to flee their homes.^87^ These countries were also ranked low in International Agreement Participation, Refugee Policies, and Refugee Accommodation Situation. Some West African countries are also facing similar crises. Prolonged armed conflicts, terrorism and ethnic repression, compounded by growing poverty, food shortages, and the climate crisis, have triggered significant refugee movements across borders from Nigeria, Mali and Mauritania to neighboring states. In North Africa, Morocco and Egypt performed well in receiving new refugee arrivals and provide asylum policies. The unstable situation in Libya, characterized by armed conflicts and political fragmentation, has displaced hundreds of thousands of people to other African countries and to Southern Europe (through the Central Mediterranean route). It is also not party to the 1951 Convention and has no concrete refugee policies, let alone the provision of decent accommodation facilities.^88^ Southern Africa has been a relatively stable sub-region in the African continent. Mozambique and Zambia are the best performers in Africa, maintaining a ranking of around 20. These two countries have achieved full marks in the two indicators of International Agreement Participation and Domestic Refugee Policy. Zambia continued to host a large Congolese refugee population.

**Oceania** Oceania performed reasonably well, with an average ranking of 100. It was the continent producing and exporting least refugees, but it scored low in receiving refugees and international refugee-agreement participation. Simultaneously, despite a slight improvement in its averaged score (see Fig. 6.2) the overall ranking of Oceania has deteriorated slightly over the past 9 years, from 87 in 2010 to 100 in 2018, mainly due to the decline in the number of RSDs and the significant fluctuations in RSD's positive ratio during the past decade. Australia and Samoa are ranked first and second at the national level in the region, with their rankings maintaining above 30th place over the past decade. New Zealand follows closely behind, stabilizing in the top 40. According to the Refugee council of Australia, Australia recognized or resettled 23,002 refugees in 2018 (1.39% of the global total). However, Australia was also criticized for its refugee policies which did not respond well to the large number of asylum-seeker arrivals and therefore not a model for the world as its leader claimed.^89^ Moreover, Australia has introduced a policy of “offshore processing centers” in Nauru and Papua New Guinea since 2012, which was designed to stop refugees arriving in Australia by sea without a valid visa. This policy has been increasingly condemned by many international organizations.

It can be observed that the origin and distribution of refugees have showcased certain obvious regional features. This is largely driven by the fact that unresolved regional conflicts and insecurity have resulted in a growing number of forced migrations and presented major challenges to the effective governance of refugee crises. Therefore, the issue of how regional bodies and individual countries in the same region might cooperate with each other to manage refugees, respond to the needs of people on the move, and create new initiatives to protect newcomers, has become more important and relevant than ever.^90^ Much effort has already been given to regional cooperation on refugee governance, for instance in the Solutions Strategy for Afghan Refugees, the Nairobi Declaration and Plan of Action on Durable Solutions for Somali Refugees and Reintegration of Returnees in Somalia, the Comprehensive Regional Protection and Solutions Framework for Central America and Mexico, and Asia Pacific Refugee Rights Network’s Vision for Regional Protection. With record numbers of people displaced around the world, comprehensive regional approaches with contributions from individual countries have become increasingly crucial in addressing this common crisis.

#### Conclusion

Refugee problems continue to pose a great challenge to the fulfillment of global justice. Forced and irregular displacement will lead to economic, social and political crisis in countries of origin, transit and destination, as well as insecurity for refugees themselves. Individual countries play a pivotal role in providing policies and assistance for managing the crisis in the short, medium, and long term. This sub-index ranks countries’ performance and contribution to refugee governance, which is designed to encourage both origin and host countries to address refugee protection and enhance global justice in a more comprehensive manner.

As shown in the above analysis, there has been a great disparity in state performance in multiple aspects of refugee governance among different regions and sub-regions, between developed countries and developing countries, between origin countries and host countries and between neighboring countries and far-away countries. The occurrence and deterioration of the refugee crisis is closely associated with armed conflicts, growing poverty, natural disasters and weak state capacity.

In order to effectively improve global justice in the domain of refugee governance, cooperation between various actors, including host countries, countries of origin, regional organizations, NGOs and UN agencies, will be undoubtedly critical to offering sustainable and comprehensive responses.

For refugee host countries, although large refugee flows have fueled xenophobic reactions and conservative migration policies in the West, these developed countries are responsible for doing more in either meeting basic needs and addressing logistical issues in short term or providing a complex institutional framework and policy measures in the long run. Because the refugee issue is a global humanitarian crisis requiring more equal burden-sharing. Developing countries next to the center of origin currently host much larger refugee populations than the Western countries that control the media discourse. For origin countries, many governments have long suffered political instability, weak state capacity, fragmented power, armed conflicts, widespread poverty, resource shortages and a lack of governance. Prevention is always better than cure. In order to address the root causes of refugee problems, more concerted and thorough efforts need to be conducted by origin countries (with support from international organizations) to promote peacebuilding, enhance economic development, reduce poverty and improve good governance. Only through these measures on multiple levels can individual countries escape the cycle of bad governance, exile, worse governance and more exile. In addition, conflict prevention and good governance will also attract more returnees back to their homelands and strengthen the resilience of their livelihoods, which will in the long term alleviate the severity of the refugee crisis in other places.

The global refugee crisis represents one of the key challenges towards achieving the objective of the 2030 Agenda for Sustainable Development adopted by the United Nations General Assembly. In today’s world where anti-migration sentiments, nationalism and populism have gained ground, it seems more critical to develop differentiated but concerted measures for refugee governance and ensure solidarity and partnership among relevant stakeholders. More country-level plans and actions, with support from regional actors and the international community,^91^ are expected to be implemented, in order for individual national states to contribute to global justice by supporting people of concern and transforming the way they respond to refugee situations.

### Issue 7: Anti-poverty

#### Introduction

Global poverty is one of the very worst and most urgent global justice problems currently facing the world. It presents a great threat to human development and to social, economic and political stability. According to estimates by the World Bank, as of 2018, at least 9% of the world’s people lived in extreme poverty, which is defined as living on only US$1.90 a day or less based on 2011 purchasing power parity (PPP). If we raise it to more moderate poverty lines, roughly 24% of the world’s population live on less than $3.20 a day and 43% on less than $5.50 a day.^92^ Poverty alleviation is of substantial importance to improving global justice. “Leave no one behind” is the central promise and the rallying cry of the 2030 Agenda for Sustainable Development. The past three decades have witnessed remarkable and unprecedented progress toward the goal of poverty alleviation, with the share of the global population living in extreme poverty continuously plunging from 36% in 1990 to less than 10% in 2018. Many national governments, especially those in East Asia and Pacific or South Asia, have invested tremendous efforts to confront poverty and reduce inequality. Meanwhile, continuous economic growth and widespread improvements in well-being in middle-income countries have made great contributions to helping tens of millions of desperately poor people escape poverty every year.

Despite this optimistic picture of world poverty reduction, the fight against global poverty is far from successful, and in certain ways is even getting more challenging. There are at least three alarming reasons for concern. First, although global poverty rates have been largely decreasing over the past 30 years, the progress of reducing poverty has been very uneven across different regions and countries of the world. For instance, in Sub-Saharan Africa, the number of extreme poor is unacceptably high and is going to rise further in the coming years, leading to regional concentration of the global poor. Second, the pace of poverty alleviation is gradually slowing down. The poverty reduction effects brought about by economic growth have begun to decrease. The fight against poverty has entered more difficult “deep water areas” where fragile states, poor governance, unresolved conflicts and low-quality infrastructure have become the biggest obstacles to a more equitable and sustainable society.^93^ Third, in 2020, the global crisis of the COVID-19 pandemic posts new challenge to poverty governance. The COVID-19 crisis has disproportionally impacted the world’s poor and is estimated to lead to an additional 150 million people falling into extreme poverty over the next 2 years.^94^ This will be the first time in over 20 years that the world will see more new poor than the number of people lifted out of poverty. Growing poverty could cause social tensions, induce political conflicts and jeopardize human development in areas such as health, education and mortality. All the above issues make the fight against poverty a more urgent and challenging global project, in which nation-states should shoulder more responsibilities and make more contributions. Without comprehensive plans and swift, significant and substantial policy actions by individual countries, years of achievement in poverty reduction will likely soon be erased. This anti-poverty sub-index, as part of the global justice index, is designed to evaluate individual countries’ efforts and performance in poverty reduction, as a means to improve global justice.

#### Dimensions and Indicators

Poverty is a state in which a person lacks a commonly acceptable amount of financial resources and essentials for a minimum standard of living in a particular place. It centers on material deprivations and the inability to satisfy their basic needs. Poverty is closely related to, but must be treated differently from*inequality* and *vulnerability*. Inequality emphasizes income or welfare distribution within the whole population; it is often measured by the Gini index. Vulnerability highlights the risk of falling into poverty in the future, which is often influenced by external shocks such as a financial crisis, a natural disaster, or a pandemic.^95^ This sub-index is focused on assessing individual countries’ performance in and contribution to global poverty reduction, referring to their achievements in helping the poor to meet basic needs. Therefore, the sub index will restrict itself from stretching its key concept of poverty too much to include dimensions of inequality and vulnerability. Of course, that is by no means to say that inequality and vulnerability are not as important as the problem of poverty; they are certainly of great significance to enhancing social justice. However, given its theoretical definitions, this project will concentrate on the issue area of absolute poverty, measuring and comparing individual countries’ contribution to global justice in the domain of protecting the most vulnerable and satisfying people’s minimum essential needs.

Although the theoretical and conceptual underpinnings of poverty measurement are anchored in social sciences,^96^ measuring and comparing nation states’ performance in poverty governance is still a big challenge. There is no single commonly accepted way to operationalize poverty measurement. Governments around the world have adopted their own indigenous methods to evaluate poverty and set poverty thresholds so as to serve their policy purposes and political aspirations. As a consequence, big cross-national variations can be observed in poverty survey methods, the indicators used, the types of data collected and the ways data are aggregated, thus making it notoriously difficult, if not impossible, to make comparisons of poverty governance across countries and across time. Moreover, there are two basic approaches to considering and measuring poverty. One is to take a “thin” perspective on poverty by measuring it through defining a threshold of individuals’ income or consumption below which people are considered as poor. The other is to take a “thick” perspective of poverty by defining it as the command over various specific types of goods such as food, education, health care, longevity and employment. The former is monetarily valued and based on a well-established poverty threshold, such as the measurement used by the World Bank. The latter is often non-monetary and relies on a sophisticated set of indicators,^97^ for example the Human Development Index (HDI) and the Multidimensional Poverty Index (MPI).^98^

Based on the goods-based conception of global justice and the principle of CDDR (as elaborated in our concept paper), we assume that efforts to combat global poverty should respect the action of individual countries involved in improving the living conditions for the least advantaged within their respective jurisdictions. Therefore, to assess the contributions made by each country to global poverty eradication, we measure their progress in poverty reduction by focusing on two thematic indictors: (1) poverty rate reduction, which measures the “contribution” dimension; and (2) poverty gap, which measures the “performance” dimension.^99^ Doing so achieves a middle ground between a single indicator of poverty measurement (the “thin” approach) and a sophisticated set of indicators for multidimensional measurement (the “thick” approach).^100^ In addition, the present sub-index of anti-poverty is only one of the ten issue areas used to construct the final global justice index. Many indicators included in multidimensional poverty measurement have already be assessed in other issue areas, such as education, public health and protection of women and children.

The World Bank is the main source of globally comparable data on global poverty^101^ and it has defined the widely agreed-upon “International Poverty Line”. These poverty lines are set as a scale, ranging from extreme to moderate levels. It is important to emphasize that the international threshold of extreme poverty (consumption expenditure at $1.9 per day in 2011 PPP) is mainly based on and reflects the situations in some of the poorest countries, which is too low to gain a comparable sense of poverty in all countries of the world. In 2017, the World Bank supplemented the international extreme poverty lines with two new ones, tracking global poverty at $3.20 a day (the line for lower-middle-income countries) and $5.50 a day (for upper-middle-income countries). In order to ensure the comparability, coverage and quality of the poverty measurement data, we use the international poverty line at $5.50 a day (see table 7.1). This higher-valued poverty threshold not only covers most underdeveloped countries but also includes many upper-middle-income countries and high-income economies, which is more relevant to current economic condition and makes the global comparisons possible.

However, the World Bank data suffer severe problems of missing values, with data appearing in certain years and missing in other years. Technical approaches can be applied to address some of the data shortcomings. More specifically, we use trends in GDP or national poverty statistics to impute missing poverty estimates by the World Bank.^102^ In order to construct the sub-index, we calculate the ranking score by assessing poverty governance in two dimensions: (1) contribution, which is measured by poverty rate reduction, referring to the extent to which a country’s effort in reducing poverty in a given year has improved compared with the year before; (2) performance, which is measured by poverty gap, representing the achievement a country has made in poverty reduction (see Table 16). The data sources currently available limited our ability to rank all nation-states, but we did our best to cover as many countries as possible.

**Table 16:** Data on anti-poverty

Category	Indicator	Data source	Coverage
Contribution	Poverty rate reduction ($5.5, population weighted)	World Bank	154 countries (145 countries have no missing values; 9 countries have 1 ~ 4 missing values)
Performance	Poverty gap ($5.5)	World Bank	154 countries (145 countries have no missing values; 9 countries have 1 ~ 4 missing values)

#### Results

Following the index construction processes and methods developed by this project (see the methodological section), this sub-index ranks 154 countries from 2010 to 2018 according to their level of performance in and contribution to global justice in the issue area of anti-poverty (see Table 17).

**Table 17:** Country ranking in anti-poverty aspect of promoting global justice

Country Name	2010	2011	2012	2013	2014	2015	2016	2017	2018
China	33	1	1	1	1	1	1	1	1
Iceland	2	13	10	11	9	2	2	2	2
Switzerland	5	2	3	2	3	3	3	3	3
Azerbaijan	1	4	4	3	4	4	4	4	4
Cyprus	11	8	2	7	6	13	6	5	5
Slovenia	16	12	15	17	14	14	10	7	6
Norway	3	6	7	6	7	5	5	6	7
Belgium	17	14	16	14	8	7	7	8	8
France	7	9	9	12	12	6	8	9	9
Netherlands	9	5	11	8	18	19	9	10	10
Czechia	22	23	24	15	27	18	24	18	11
Austria	8	19	18	5	20	12	12	12	12
Malta	14	10	17	9	2	8	11	11	13
Luxembourg	13	7	8	4	5	10	14	13	14
Finland	10	3	12	10	11	9	13	14	15
Germany	6	11	13	13	10	11	15	15	16
Denmark	4	25	6	19	23	16	16	16	17
Ireland	24	22	23	27	21	17	19	19	18
Australia	15	17	14	16	15	15	17	17	19
Slovakia	20	21	21	20	22	23	23	25	20
United Kingdom of Great Britain and Northern Ireland	23	15	5	18	13	25	18	20	21
Croatia	19	20	22	21	24	22	21	21	22
Canada	12	18	19	24	17	21	22	22	23
Sweden	21	16	20	23	19	20	20	23	24
Belarus	32	32	28	25	16	27	26	27	25
United States of America	26	27	27	28	28	28	28	24	26
Japan	18	24	25	22	25	24	25	26	27
Republic of Korea	25	26	26	26	26	26	27	28	28
Poland	39	37	38	39	41	37	29	30	29
Italy	29	28	30	29	29	29	30	29	30
Hungary	35	33	33	35	33	32	33	31	31
Spain	28	29	29	31	30	31	31	32	32
Malaysia	45	43	41	34	35	34	34	34	33
Portugal	30	31	36	36	34	30	32	33	34
Russian Federation	31	30	31	30	31	35	35	35	35
Bosnia and Herzegovina	34	34	34	37	36	36	37	36	36
Lithuania	41	40	40	40	40	39	39	38	37
Latvia	42	42	42	42	43	42	42	40	38
Montenegro	37	41	44	51	42	40	38	37	39
Lebanon	NA	NA	NA	32	32	33	36	39	40
Israel	36	36	35	38	37	38	40	41	41
Uruguay	43	38	39	41	39	41	41	42	42
Thailand	53	51	54	53	53	44	47	44	43
Bulgaria	40	44	43	46	45	45	44	45	44
Greece	27	35	37	44	44	43	43	43	45
Serbia	49	52	53	54	51	47	49	47	46
Seychelles	48	47	46	47	47	46	48	46	47
Ukraine	38	39	32	33	38	52	46	48	48
Estonia	47	49	48	50	48	48	50	49	49
Kazakhstan	56	45	47	43	46	49	52	51	50
Iran (Islamic Republic of)	44	46	45	45	55	53	45	50	51
Chile	52	53	51	48	49	50	53	53	52
Turkey	50	48	52	49	50	54	51	52	53
Costa Rica	46	50	50	52	52	51	54	54	54
Mauritius	57	57	57	58	58	57	57	55	55
Republic of North Macedonia	51	55	56	56	57	55	56	57	56
Panama	54	54	55	57	56	56	55	56	57
Republic of Moldova	70	66	67	61	62	58	59	58	58
Romania	64	65	65	69	65	63	60	59	59
Dominican Republic	68	69	72	73	69	64	62	62	60
Paraguay	62	61	58	59	59	59	58	61	61
Brazil	55	56	49	55	54	60	63	60	63
Bolivia (Plurinational State of)	60	59	59	60	61	61	61	64	64
Viet Nam	73	89	80	81	78	74	70	67	65
Peru	61	62	60	62	64	62	65	65	66
Ecuador	66	64	64	67	63	65	64	66	67
Tonga	67	67	68	72	72	69	67	68	68
Tunisia	72	70	69	71	70	71	71	71	69
Colombia	69	68	70	70	68	68	69	70	70
Palestinian Territories	59	60	62	64	66	67	68	69	71
Jordan	58	58	61	65	67	70	72	72	72
Mongolia	83	74	66	63	60	72	80	77	73
Morocco	86	76	77	78	76	76	78	73	74
El Salvador	76	81	78	77	79	78	74	74	75
Albania	89	90	93	95	87	85	83	79	76
Jamaica	71	71	73	74	75	77	75	76	77
Algeria				75	74	75	73	75	78
Gabon	91	86	81	82	81	81	79	78	79
Mexico	74	73	75	84	93	80	77	81	80
Central African Republic	88	88	92	76	77	79	81	82	81
Tuvalu	NA	93	97	98	98	86	87	85	82
Burundi	82	83	84	88	88	84	84	84	83
Madagascar	75	75	76	79	80	82	82	83	84
India	130	96	99	113	103	99	91	89	85
Honduras	78	79	90	92	90	94	86	86	86
Venezuela (Bolivarian Republic of)	63	63	63	66	71	73	76	80	87
Samoa	85	78	88	93	96	91	85	87	88
Bhutan	98	97	94	96	95	93	92	90	89
Armenia	141	140	132	131	115	110	101	95	90
Sri Lanka	125	120	112	111	104	100	95	93	91
Democratic Republic of the Congo	65	72	74	80	82	83	88	88	92
Georgia	115	109	106	100	99	101	102	98	93
Botswana	92	91	89	87	85	90	93	94	94
Malawi	80	77	79	83	83	87	90	91	95
Namibia	96	98	95	97	94	89	89	92	96
Guatemala	77	82	85	91	97	95	96	97	97
Zambia	79	80	82	85	86	88	94	96	98
South Africa	90	92	91	94	92	96	99	99	99
Lesotho	81	84	87	90	91	98	100	100	100
Fiji	108	106	107	108	110	103	104	103	101
Mozambique	87	85	86	89	89	97	98	101	102
Guinea-Bissau	84	87	83	86	84	92	97	102	103
Maldives	104	103	105	103	100	105	107	105	104
Cabo Verde	97	100	98	101	105	104	105	106	105
Tajikistan	131	132	131	128	116	116	113	107	106
Indonesia	111	121	134	123	128	128	103	104	107
Ghana	116	112	109	109	112	113	114	109	108
Comoros	102	105	104	105	106	106	108	110	109
Eswatini	100	101	102	104	107	108	110	111	110
Benin	101	104	101	102	101	102	106	108	111
Cameroon	117	114	110	110	111	109	111	112	112
Nigeria	99	102	103	107	109	112	117	114	113
Philippines	124	127	127	122	123	123	119	118	114
Congo	109	110	108	112	113	114	118	116	115
Togo	95	99	100	106	108	111	116	117	116
Nicaragua	123	125	118	116	114	115	115	113	117
Yemen	143	144	145	148	144	141	132	122	118
Rwanda	93	94	96	99	102	107	112	115	119
Chad	112	115	116	117	119	119	121	120	120
Uganda	114	116	122	130	125	120	122	121	121
Zimbabwe	NA	NA	119	121	121	121	123	123	122
Myanmar	NA	NA	NA	NA	NA	NA	141	135	123
Angola	120	124	121	120	120	122	125	124	124
Sudan	135	131	117	124	124	117	120	119	125
Papua New Guinea	NA	123	125	125	127	127	126	125	126
Sierra Leone	110	113	128	138	138	118	124	127	127
Liberia	103	108	115	134	136	135	133	130	128
Kenya	118	118	120	119	122	126	127	128	129
Micronesia (Federated States of)	113	117	123	132	131	131	130	129	130
Mauritania	122	126	126	129	126	132	135	132	131
Egypt	145	148	149	153	149	144	143	142	132
Iraq	132	135	133	126	135	138	109	126	133
Niger	105	119	124	127	130	130	131	131	134
Cote d'Ivoire	126	129	130	135	133	134	137	136	135
Mali	119	122	114	114	117	125	129	133	136
United Republic of Tanzania	106	107	111	115	118	124	128	134	137
Senegal	127	128	129	133	132	133	136	138	138
Haiti	NA	NA	NA	136	134	136	138	139	139
Kiribati	139	143	141	141	143	139	140	140	140
Burkina Faso	107	111	113	118	129	129	134	137	141
Ethiopia	136	136	135	137	137	137	139	141	142
Vanuatu		137	139	140	139	140	142	143	143
Timor-Leste	133	138	140	144	147	148	149	145	144
Solomon Islands	128	133	137	143	140	142	144	144	145
Gambia	129	134	136	139	141	145	145	146	146
Nepal	140	146	147	150	150	150	151	148	147
Uzbekistan	134	139	144	147	146	146	147	147	148
Guinea	121	130	138	142	142	143	146	149	149
Lao People's Democratic Republic	138	142	143	145	145	147	148	150	150
Pakistan	146	149	150	152	153	152	152	153	151
Bangladesh	144	145	146	149	151	151	153	152	152
Sao Tome and Principe	137	141	142	146	148	149	150	151	153
Kyrgyzstan	142	147	148	151	152	153	154	154	154

The second decade of the twenty-first century has witnessed great progress in reducing global poverty, with an unprecedented number of people around the world being lifted above basic needs. According to the World Bank, in 2010, about 54% of the world population was living on less than US$5.50 a day. By 2018, this figure had fallen to approximately 43%, compared with roughly 24% and slightly less than 10%, respectively, living below the US$3.20 and US$1.90 poverty lines. Meanwhile, the poverty gap at US$5.50 a day, which reflects the intensity of poverty in a nation, decreased from 0.27 in 2010 to estimated 0.19 in 2018. However, millions of individuals are still trapped in poverty and suffer from life challenges, not only in low-income countries but also in middle-income and even high-income countries.

**Fig. 14:**
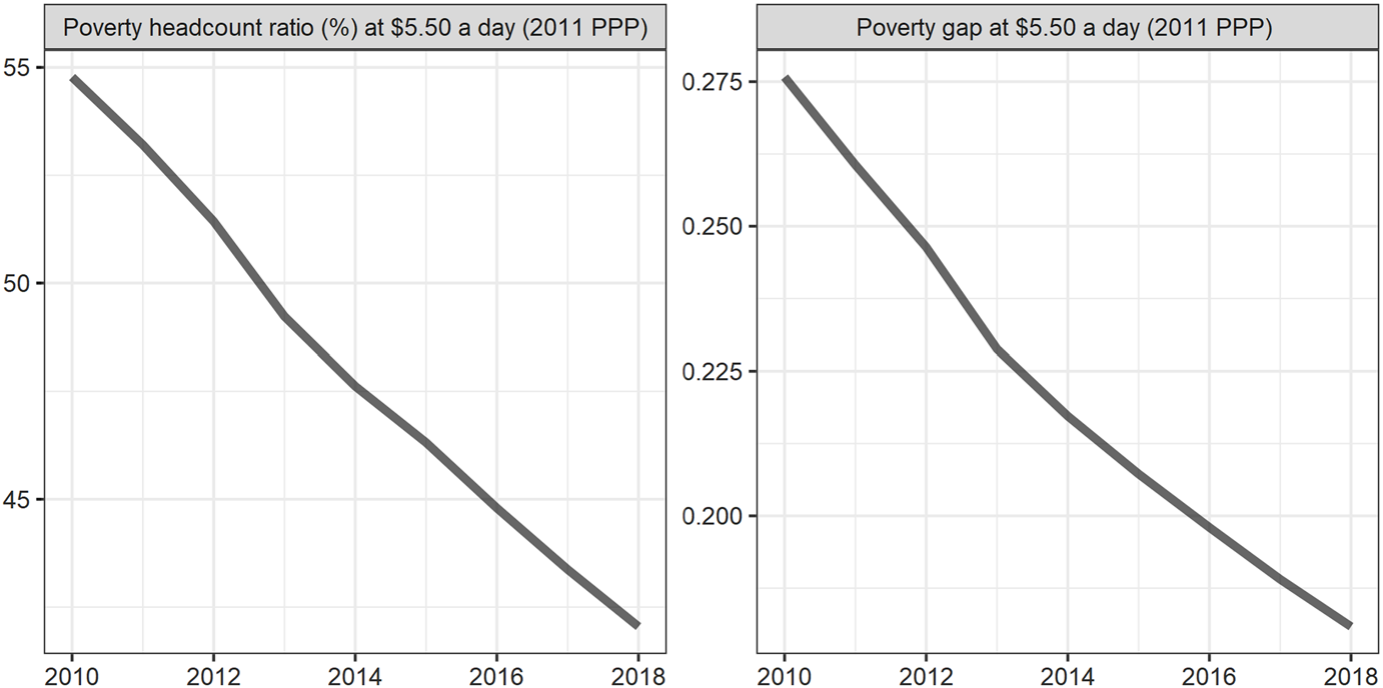
The world’s poverty headcount rate and poverty gap in 2010–2018

Despite having reduced considerably, progress in poverty alleviation has been very uneven. The countries in East Asia (especially China) experienced the largest reductions in the proportion of people living on less than US$5.50 a day (Fig. 14). However, in comparison, more than two-thirds of the population in Africa are still living below the same threshold. Moreover, although steady progress has been made towards the US$5.50 a day threshold, a slower pace in poverty reduction at this higher line is observed compared to the thresholds of US$1.90 a day and US$3.20 a day.^103^ This illustrates that as the world has grown richer, fighting extreme poverty is not sufficient for people to live a life free of poverty, and the task of poverty alleviation becomes even more difficult if we set the goals higher. In addition, the year 2018 brought some new challenges to global poverty governance, including the conflicts and violence in Syria, Afghanistan, Yemen, Democratic Republic of the Congo and the Central African Republic, economic crises in Venezuela and some Southern European countries, as well as rising sea levels and temperatures caused by the changing climate all constitute main drivers of the slow-down in global poverty reduction. Therefore, as we celebrate remarkable successes in lifting millions of people out of poverty, we must realize that more challenges lie before us and some of these challenges may even lead to a reversal of poverty reduction.

According to the anti-poverty index constructed in this project (seetable 7.2), China is leading the world in promoting global justice in the issue area of poverty alleviation, a position it has maintained since 2011. The poverty headcount rate at US$5.50 a day in China dramatically dropped from 53.4% to 18.9%, with the poverty gap simultaneously falling from 1.67 to 0.771. This may not be surprising given the fact that the rapid and continuous economic growth in China over the past decade has lifted millions out of poverty. More importantly, this tremendous progress is also attributed to the huge investment and efforts made by the Chinese government in poverty eradication. Over the past few years, the Chinese government’s efforts against poverty have greatly intensified as the leadership of President Xi Jinping proposed an ambitious campaign of “targeted poverty alleviation”, as one of four “tough battles”, to eliminate absolute poverty and build a moderately prosperous society by the end of 2020.^104^ Based on China’s national poverty threshold, the number of people living in absolute poverty nationwide has decreased from 98.99 million at the end of 2012 to 5.51 million at the end of 2019, with the poverty headcount being reduced by more than 10 million annually for seven consecutive years. UN Secretary-General António Guterres called China’s success as “the greatest anti-poverty achievement in history”.^105^ Although many obstacles remain ahead of China’s efforts to attain its ambitious goal and maintain its sustainable success, the country has presented the world with a role model in a sense that the government takes up its responsibility to help impoverished people. In 2018, the Chinese government launched a “Global Poverty Reduction & Inclusive Growth Platform”^106^ to promote the exchange of experience and knowledge sharing on poverty reduction with the rest of the world. European states and other OECD countries also have made great contributions to global justice in the domain of poverty reduction. This largely results from their relatively high income levels and stable economic development, as well as their income distribution policies and social welfare projects.

**Fig. 15:**
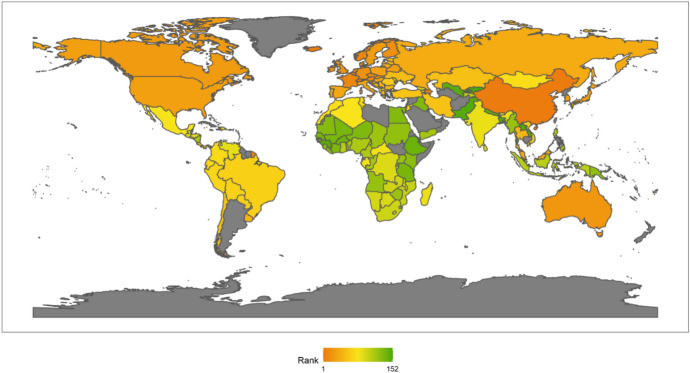
2018 Index ranking of anti-poverty on a world map

Among the ten countries ranked top in 2018 (see Fig. 15), three belong to Asia (including China, Azerbaijan, and Cyprus) and seven are in Europe (including Iceland, Switzerland, Slovenia, Norway, Belgium, France and the Netherlands). The above world map shows the ranking of all observed countries in 2018. Compared with the results for 2017 in our last annual report, the change is obvious. This is largely due to changes in the way the anti-poverty sub-index is constructed and measured. The 2018 index focuses only on absolute poverty and does not include the Gini coefficient, an indicator of relative poverty. In doing so, it is more reflective of the contribution and performance of individual countries in promoting global justice by addressing the basic needs of humankind. With the optimization of our index construction methods, it is normal for ranking fluctuations to occur between years.

Among the bottom-ranked are developing countries in Africa, Asia and the Pacific region. In these underdeveloped countries, more than half of the civilian population are living in conditions far below the international poverty threshold of the US$5.50 a day. For example, in Kyrgyzstan, the poverty headcount rate has been higher than 64% and the poverty gap has stayed larger than 2.45 over the past 9 years. In Bangladesh, the poverty headcount rate reached a peak of 87.73% in 2010 and it has slowly decreased to 79.31% during the past decade. However, at the same time, the country’s poverty gap increased from 2.45 to 2.52. A similar pattern was observed in Pakistan, Laos, and Sao Tome and Principe. Various pressing issues including rural underdevelopment, fast-growing population, economic and political uncertainty, lack of education and climate change pose barriers to poverty reduction in these countries.

#### Regional Analysis

**Fig. 16:**
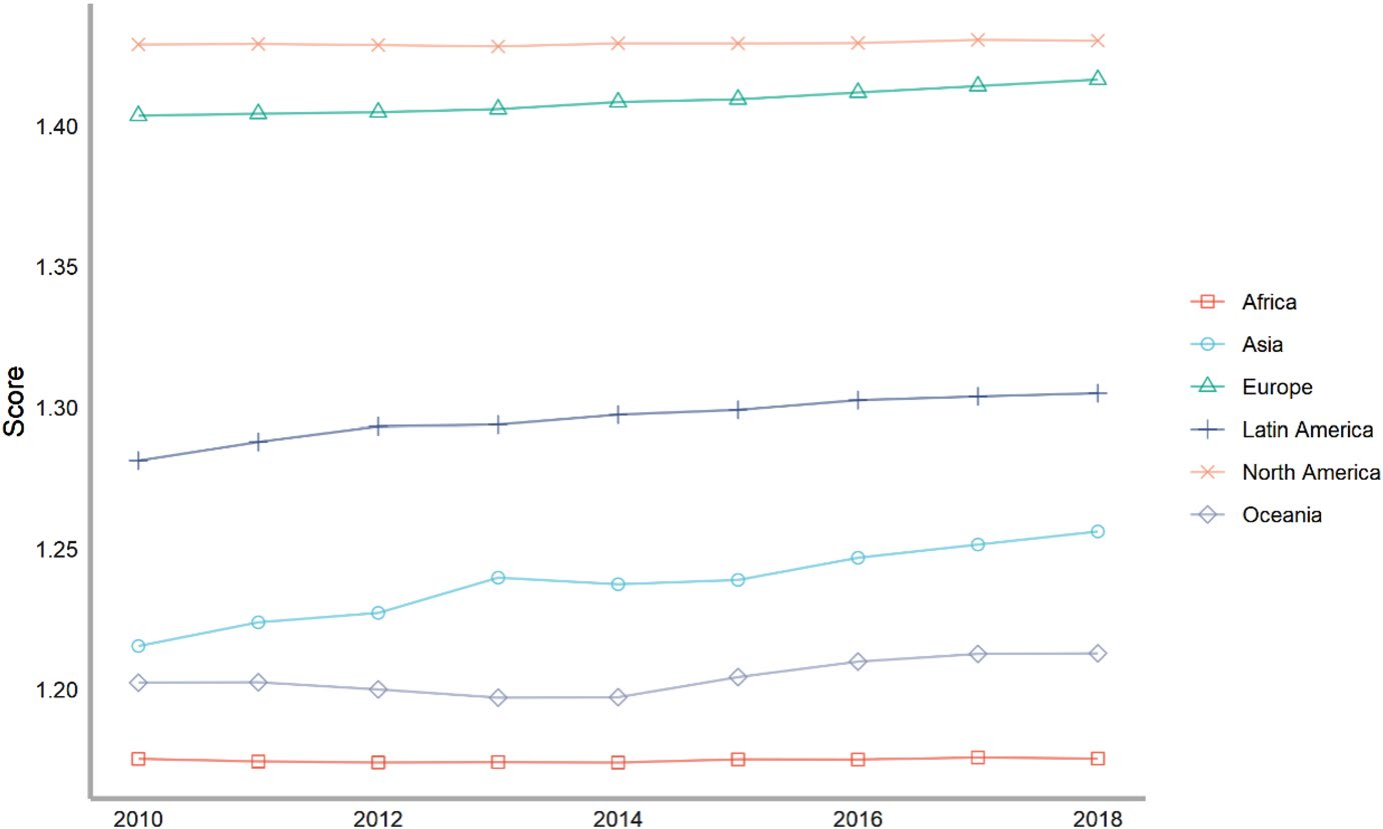
The score of anti-poverty issue across continents, 2010–2018

As shown in Fig. 16, all of the six regions in the world have experienced slight improvements in poverty governance aspect of global justice in recent years. However, this progress has been regionally uneven. The geographic breakdown of regions with the ranking of anti-poverty aspect of promoting global justice ranging from best to worst include: North America, Europe, Latin America, Asia, Oceania and Africa.

**Asia** Asia as whole has a relatively high poverty headcount ratio and poverty gap compared to other continents. However, the average Asian score has shown the largest improvement compared to other regions over the past decade, with its poverty headcount rate dropping dramatically from 64.68% to 45.22% and the poverty gap steadily falling from 0.310 to 0.173, measured by the international poverty line of the US$5.50 a day. This is because Asia has achieved a remarkable economic rise which has served to help hundreds of millions of Asians rise out of poverty. Furthermore, national governments like China, India, and Malaysia have invested heavily in implementing comprehensive anti-poverty policies, taking up their responsibilities to meet people’s basic needs. At the sub-regional level, different sub-regions of Asia have performed quite unevenly in promoting global justice through poverty reduction. East Asia is the star performer, showing the strongest sub-regional score and the biggest decline in the poverty headcount ratio, and maintaining the lowest poverty gap in the region. It is not surprising that the leading contributor of East Asian poverty decline is China, whose fast economic growth and ambitious targeted poverty alleviation program have helped lift millions of people out of absolute poverty. The two more prosperous countries in East Asia, Japan and South Korea, have kept their poverty at a low level. Much of the progress has also been in Southeast Asia. Countries like Malaysia, Thailand, and Vietnam outperformed the world average. South Asia and the Central Asia have been relatively poor regions, but their poverty ratio and poverty gap have also been improving at a moderate rate over the past years. However, it must be noted that Asia is still far away from ending moderate poverty, given the reality that there are still over 1900 million Asians living below the US$5.50 poverty line. Moreover, slowing economic growth, unresolved sub-regional instability, deepening rural–urban division and the devastating COVID-19 pandemic pose new challenges to the ongoing fight against poverty.

**Europe** Europe’s average index score wins second place, closely following North America. European countries account for 15 of the top 20 countries in the 2018 ranking, with Iceland being the highest ranked country in the region and the second worldwide. During the observation years, the anti-poverty index score has recorded a slow and steady increase, driven by progress in reducing both the poverty headcount ratio and poverty gap on the continent. In 2018, Europe has reached its lowest ever poverty headcount ratio of 2.38% and lowest poverty gap of 0.00794, measured by the international poverty line of US$5.50 a day. In terms of sub-regional variations, Western Europe and Northern Europe maintained their leading performance in keeping the poverty headcount ratio and the poverty gap low. Eastern European countries are lagging behind but had the biggest improvements in the region in recent years, with the poverty headcount ratio decreasing from 6% to approximately 3% and the poverty gap from about 0.36 to 0.17 over the past decade. This was mainly due to steady economic growth in this sub-region following European integration. Southern Europe recorded a slight deterioration in the average index score, with rising poverty from 2010 to 2013. This was largely attributed to the European debt crisis in which debt-laden countries such as Greece, Italy and Spain suffered heavily from stagnant economies and a high unemployment rate. As its economic situation became relatively more stabilized, both the poverty headcount ratio and the poverty gap in southern Europe started to fall from 2013.

**North America** The North American region has maintained the world’s highest average index score with little variation from 2010 to 2018. The poverty headcount ratio below the international poverty line in North America has remained at around 1.6% and its poverty gap has been kept as low as roughly 0.011. Within the region, Canada performed better than the United Studies, ranked 23rd versus 26th globally in 2018, thus contributing more to global justice in the domain of poverty governance.

**Latin America** Latin America, which includes 19 countries in this research, shows an overall moderate increase in the anti-poverty index score. Both the poverty headcount ratio and the poverty gap have experienced an obvious reduction in the region over the observation years, despite some temporary fluctuations in some countries. This progress was largely driven by the well-known poverty reduction strategy of Conditional Cash Transfers (CCTs) which has long been implemented in countries across the region. CCTs are an innovative poverty alleviation policy which is designed to support the poor by offering them cash payments upon conditions that the recipients participate in activities that develop human capital, such as school attendance, health-care checkups, and nutritional services. This policy helps to break the intergenerational transmission of poverty and will generate long-term positive effects on poverty eradication. Latin American countries including Chile, Mexico, Peru, Brazil, Colombia, Jamaica, Honduras and Nicaragua have used CCTs programs to fight poverty since the late 1990s,^107^ and their effects have become increasingly apparent in the twenty-first century. Looking at the sub-regions, South America outperformed Central America and the Caribbean in contributing to global justice in terms of poverty governance. However, the pace of poverty reduction has obviously slowed in all the sub-regions in recent years. After years of sustained economic growth, some Latin American countries have fallen into an economic crisis, confronting growing unemployment rates and deteriorating welfare. An illustrative case is Venezuela. Although Venezuela used to be the strongest economy in Latin America (because it has the largest oil reserves in the world), the poverty rate and poverty gap have increased dramatically as a result of the economic collapse since the new president Nicolas Maduro came into office in 2013.

**Africa** Africa is the poorest continent and remains the bottom performer in promoting global justice in the domain of poverty governance. Although some African countries have made significant accomplishments in the fight against extreme poverty, the region as a whole recorded the highest poverty headcount ratio and poverty gap in the world. The past few years have witnessed the slowing pace of poverty reduction and a growing number of poor populations in Africa. Africa now accounts for most of the world’s poor. The main drivers behind these major poverty challenges include the increasing number of violent conflicts, political unrest and terrorism events since 2010, the much slower economic growth rate, and the poor governance of fragile states. These factors render poverty more entrenched and harder to root out in Africa. Meanwhile the region’s rapidly expanded population growth,^108^ especially in Sub-Saharan Africa, further makes the total number of people living in poverty continue to increase by millions each year. It is forecast that global poverty will become increasingly concentrated in Africa,^109^ which makes it even harder for the continent to escape poverty trap. Despite the region-wide trend, the status of poverty governance shows remarkable variations across different sub-regions in Africa. Not surprisingly, North Africa performs much better than its other African counterparts, and has displayed significant poverty reduction over the years studied. The average yearly number of people lifted from in Egypt and Morocco is 0.773 million and 0.399 million, respectively. By contrast, the poverty headcount ratio in the East, West and Central Africa has been higher than 85%. Nigeria, the Democratic Republic of Congo, Tanzania, Ethiopia, Uganda and Kenya account for about half of the number of poor in Africa. West Africa has the highest poverty gap and continues to increase, with its average poverty gap reaching 0.490 in 2018. These regional disparities are predicted to become even larger over time because of high population growth and continued armed conflicts in the East, West and Central Africa.

**Oceania** The poverty situation in Oceanian countries has not changed much over the years. Australia and New Zealand have been the two best performers in controlling poverty while the other countries (mostly island countries) lag far behind. Australia, the biggest country in Oceania, was ranked first in the region and 19th globally in 2018. As shown in Fig. 7.2, the Oceanian overall anti-poverty index score first recorded a very slight deterioration during 2010–2014 but then increased in 2014–2018. Oceania encompasses twelve other island nations with small populations. The Solomon Islands was ranked lowest in the region. Natural disasters, ethnic tensions, income disparities, rural underdevelopment and physical isolation from the international markets have exacerbated poverty in the country.^110^ As a result of climate change, some Oceanian countries like Tuvalu and Kiribati are starting to disappear under the rising sea. These environmental challenges render the people of these island countries at risk.

#### Conclusion

A world with global justice is a world free of poverty. As Mandela described, “it[poverty] is man-made, and it can be overcome and eradicated by the action of human beings. And overcoming poverty is not a gesture of charity. It is an act of justice.”^111^ For the past decade, absolute poverty has been steadily declining, which shows in the reduction of both the poverty headcount ratio and poverty gap in most parts of the world. However, still too many are trapped in extreme poverty and struggling for basic human needs. This reveals there is still much work to be done in the future. To further complicate this situation, economic crisis, armed conflict, and climate change are generating far-reaching implications for poverty reduction in the sense that they not only offset the previously achieved anti-poverty progress but also throw a growing number of people into poverty. Moreover, the disruption of the COVID-19 pandemic has brought about a new obstacle to global poverty governance, adding millions of new poor to the already-decelerated pace of poverty reduction.^112^ The decreases in the poverty ratio and poverty gap are projected to be the worst setbacks for a generation. All these factors make it increasingly challenging to maintain the momentum of progress and meet the global target under the 2030 Sustainable Development Agenda on poverty reduction.

This sub-index is mainly focused on a “thin” concept of poverty. If we take a broader view of poverty by considering additional non-monetary dimensions, the current situation could become even more alarming and worrying. The fight against poverty is not only about solving people’s basic survival needs, but also working to provide people with more welfare and rights for human development. Viewed from this perspective, eradicating poverty in all its forms calls individual countries to develop a more comprehensive strategy, ranging from multidimensional anti-poverty policies to human capital building and to institutional reforms. In many places in the world, sovereign states cannot adequately address all these issues by acting alone. Concerted and collective efforts within a country or across countries are needed to further improve the effectiveness of poverty governance.

### Issue 8: Education

#### Introduction

Education is not only a fundamental human right in itself but also an indispensable means of realizing other human rights, and thus is not only one of the elements of global justice but also an important tool for achieving global justice. Educational justice itself is an important factor embedded in the development of human society. On the one hand, more equitable education will build a more harmonious and civilized society by deepening people's awareness of public health, gender equality and political participation. On the other hand, educational inequality is closely related to the unbalanced distribution of power and wealth, and the intergenerational transmission of poverty caused by educational inequality will even aggravate the serious polarization of power and wealth, hinder social class mobility, and affect the progress of human society.

However, educational inequality in education is still one of the most significant challenges facing mankind in today's world. It is estimated that approximate 262 million of the world’s school-age population were out of school in 2017, at 64 million (9%) for children of primary school age, 61 million for adolescents of lower secondary school age (16%), and 138 million (36%) for youth of upper secondary school age.^113^ The situation is particularly bad in low-income countries. Fewer than three out of four children began school on time in low-income countries in the period of 2000–2016.^114^

In this report, we focus on the role of states in protecting the citizens’ right of access to basic education in terms of global justice. First, governments are “*the primary duty bearers of the right to education*”.^115^ As protector of rights and provider of basic goods and services, states play a key role in protecting citizens’ right to education and promoting equality in education by making rules for allocating educational resources and directly and indirectly investing education. Thus, the Convention on the Rights of the Child and the International Covenant on Economic, Social and Cultural Rights explicitly highlight states’ obligations to realize the right to children’s education, which is consistent with the principle of “cosmopolitan but due-diligent responsibilities” (CDDR).^116^ Second, education as a human right usually refers to basic primary education. If children have obtained primary basic education, they will likely be literate and numerate, then will have the basic skills necessary to get a job in order to have a fulfilling life.^117^ Therefore, UNESCO’s aim to construct a twenty-first-century learning society by promoting the Millennium Development Goal (MDG) to achieve universal primary public education for all by 2015.

#### Dimensions and Indicators

We measure educational justice from two perspectives. The first is the performance of each country in education. As discussed earlier, from the perspective of global justice, we pay attention to the performance in basic education. We, therefore, use the primary education-related indicators to measure the performance of each country in primary education, as last year's report did. In many countries, lower secondary education is compulsory for school-age children. Therefore, incorporating lower secondary education into educational performance will enhance the effectiveness of the measurement. Specifically, we will use four indictors to measure the performance in both primary and secondary schools, namely, completion rate, school enrollment, pupil-teacher ratio and dropout rate. The second is the contribution of each country to education. We use the government education expenditures of each country to measure its efforts to promote its people’s education. All education-related indexes come from the World Bank.^118^ The details are shown in Table 18.

**Table 18:** Data on education

Category	Dimension	Indicator	Data Source	Coverage
Performance	Primary education	Primary completion rate, total (% of relevant age group)	World Bank	137–142 (2010–2017)
		School enrollment, primary (% net)		
		Pupil-teacher ratio, primary		
		Children out of school (% of primary school age)		
	Secondary education	Lower secondary completion rate, total (% of relevant age group)		
		School enrollment, secondary (% net)		
		Pupil-teacher ratio, secondary		
		Adolescents out of school (% of lower secondary school age)		
Contribution	Government expenditure on education	Government expenditure on education, total (% of GDP)		

#### Results

We improved our model of global justice this year, which enabled more countries to be included in the education rankings. Compared with the 2010–2017 ranking results in the 2019 report that only included 76–105 countries, this year’s report includes 137–142 countries’ ranking results ranging from 2010 to 2018, making our results have better representative.

**Fig. 17:**
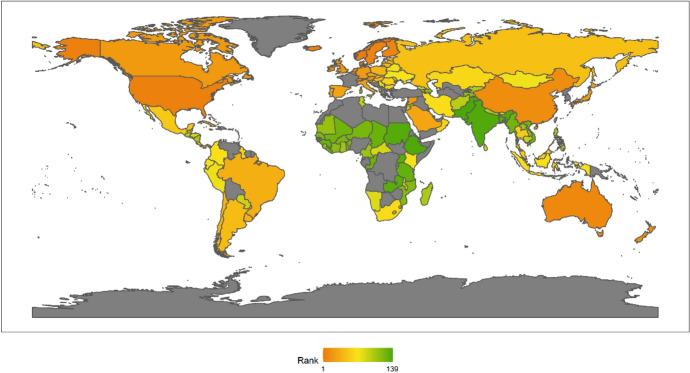
2018 Index ranking of education on a world map

Table 19 shows the ranking of countries in education issues from 2010 to 2018. Taking 2018 as an example, the top 10 countries of the ranking are Norway, Iceland, Denmark, Switzerland, the United States, Sweden, Finland, the Netherlands, Australia and Belgium, all of which are developed countries. And the bottom ten countries are Pakistan, Ethiopia, India, Sudan, Uganda, Zambia, Gambia, Bangladesh, Liberia and United Republic of Tanzania, all of which is underdeveloped countries. And Fig. 17 shows the distribution of the countries this issue covers and the ranking of education across countries in 2018.

**Table 19:** Country ranking in education aspect of promoting global justice

Country	2010	2011	2012	2013	2014	2015	2016	2017	2018
Norway	1	1	1	1	1	1	1	1	1
Iceland	11	11	8	8	6	7	3	2	2
Denmark	2	2	4	2	2	2	2	3	3
Switzerland	3	3	5	4	5	3	5	5	4
United States of America	5	6	6	6	7	6	6	6	5
Sweden	4	4	2	3	4	5	4	4	6
Finland	9	8	9	7	8	9	7	7	7
Netherlands	7	7	11	9	10	11	8	8	8
Australia	6	5	7	5	9	8	9	9	9
Belgium	12	12	16	14	13	12	12	11	10
Ireland	13	17	17	19	18	18	19	18	11
China	16	16	10	11	11	14	13	12	12
Monaco	20	15	14	18	22	21	10	13	13
New Zealand	14	13	13	13	14	13	11	10	14
Austria	15	14	18	15	17	16	14	14	15
Qatar	8	10	15	10	16	17	20	23	16
Israel	24	22	23	21	20	19	17	16	17
United Kingdom of Great Britain and Northern Ireland	17	18	20	16	15	10	15	17	18
Syrian Arab Republic	61	52	44	48	40	32	26	21	19
Canada	10	9	12	12	12	15	16	15	20
Germany	19	20	21	17	19	20	18	19	21
Japan	18	19	19	20	21	22	21	20	22
Cyprus	21	21	22	23	24	23	23	22	23
Andorra	23	25	24	27	26	24	22	24	24
Malta	27	24	28	22	23	27	25	25	25
Italy	22	23	25	24	25	25	27	26	26
Spain	25	26	27	26	28	28	30	28	27
Saudi Arabia	30	28	26	25	27	26	28	27	28
Slovenia	28	30	30	29	30	31	33	30	29
Portugal	26	29	31	28	31	33	31	31	30
Estonia	34	36	39	35	34	36	36	34	31
Czechia	33	33	37	38	41	30	32	32	32
Cuba	36	38	38	37	37	34	34	33	33
Barbados	29	32	34	34	32	29	35	38	34
Brazil	31	31	32	30	33	38	37	35	35
Latvia	52	46	36	32	39	41	44	41	36
Uruguay	48	37	35	31	35	35	38	36	37
Chile	49	48	48	42	46	43	41	40	38
Poland	37	40	41	40	42	45	42	42	39
Slovakia	39	42	46	43	43	40	45	43	40
Hungary	50	47	55	52	49	48	49	44	41
Lithuania	41	41	45	44	45	46	51	46	42
Argentina	43	39	40	39	44	37	40	37	43
Costa Rica	51	51	49	45	47	39	39	39	44
Panama	69	74	67	56	58	52	43	52	45
Russian Federation	35	34	33	33	36	44	48	45	46
Saint Kitts and Nevis	46	50	50	47	48	55	54	49	47
Croatia	45	49	54	50	53	53	55	50	48
Seychelles	57	78	114	63	57	49	46	47	49
Lebanon	47	56	53	51	54	56	50	55	50
Mexico	44	44	43	41	38	42	47	48	51
Bahrain	42	53	51	49	52	47	53	54	52
Thailand	54	54	52	54	63	67	56	59	53
Malaysia	53	45	47	46	51	51	57	57	54
Bulgaria	56	55	56	53	55	58	62	60	55
Mauritius	73	71	69	66	56	54	58	56	56
Romania	63	66	71	71	67	71	70	71	57
Oman	40	43	42	36	50	50	59	58	58
Guyana	77	79	82	69	64	64	60	61	59
Kazakhstan	70	59	60	60	66	62	65	63	60
Saint Vincent and the Grenadines	67	80	103	67	72	63	63	62	61
Saint Lucia	66	67	68	64	62	59	52	53	62
South Africa	55	57	58	58	61	60	61	66	63
Iran (Islamic Republic of)	71	63	65	76	79	82	73	70	64
Indonesia	75	64	62	61	70	78	85	82	65
Colombia	59	58	61	57	65	72	72	68	66
Kenya	65	73	66	78	74	75	68	75	67
Eswatini	79	72	72	77	77	74	77	74	68
Belize	74	75	70	74	75	68	66	64	69
Ukraine	68	69	64	65	73	81	81	78	70
Ecuador	80	76	73	68	68	66	67	67	71
Belarus	62	62	59	59	60	70	74	72	72
Mongolia	64	60	57	55	59	69	69	73	73
Peru	82	83	77	70	69	61	71	69	74
Fiji	89	90	84	87	84	79	79	80	75
Jamaica	60	65	74	72	76	73	76	76	76
Serbia	78	70	75	73	78	76	78	77	77
Vanuatu	72	68	63	62	71	65	64	65	78
Bhutan	96	93	88	85	81	80	83	79	79
Republic of Moldova	85	81	78	79	82	83	90	81	80
Namibia	58	77	76	81	86	84	87	85	81
Honduras	NA	NA	NA	94	93	93	NA	NA	82
Cabo Verde	84	92	83	83	85	85	89	86	83
Central African Republic	94	99	92	99	105	103	92	93	84
Georgia	83	84	91	88	91	91	88	83	85
Malawi	88	86	80	82	88	95	108	113	86
Comoros	81	85	79	75	80	86	80	88	87
Paraguay	109	98	95	108	109	109	107	106	88
Sri Lanka	98	97	93	103	108	97	75	89	89
Cambodia	115	113	107	116	113	115	100	118	90
Tunisia	97	100	101	104	106	92	97	96	91
Albania	90	88	85	84	87	88	86	87	92
El Salvador	93	104	97	93	94	87	91	91	93
Djibouti	99	118	109	110	111	99	98	97	94
Jordan	NA	NA	NA	NA	NA	NA	84	95	95
Afghanistan	86	91	81	100	90	106	93	92	96
Lesotho	91	87	86	86	89	89	96	107	97
Kyrgyzstan	92	95	90	89	95	98	99	99	98
Nepal	124	125	121	120	124	119	115	115	99
Guatemala	103	101	94	96	98	94	102	100	100
Madagascar	100	105	105	106	107	108	94	105	101
Nicaragua	101	102	96	97	96	96	101	98	102
Maldives	132	129	128	129	132	118	117	112	103
Philippines	107	109	104	102	92	90	95	94	104
Ghana	87	89	89	91	100	101	104	104	105
Lao People's Democratic Republic	106	108	100	98	99	104	105	103	106
Timor-Leste	105	107	102	101	102	102	103	102	107
Cameroon	113	114	106	111	112	112	116	110	108
Armenia	102	94	87	95	101	107	106	101	109
Mauritania	114	82	98	90	104	105	109	108	110
Sierra Leone	108	111	110	107	118	111	110	109	111
Tajikistan	120	122	120	121	123	117	119	121	112
Azerbaijan	104	103	99	105	103	110	118	116	113
Togo	112	110	108	109	110	113	120	119	114
Cote d'Ivoire	121	121	118	123	115	121	113	111	115
Rwanda	127	106	117	112	121	116	114	117	116
Burundi	110	117	115	115	116	114	112	114	117
Mozambique	119	119	116	118	122	126	123	125	118
Benin	123	123	123	119	125	123	121	122	119
Senegal	126	124	124	122	126	124	125	126	120
Burkina Faso	118	112	113	113	117	120	122	120	121
Niger	122	120	119	124	120	125	126	132	122
Viet Nam	125	130	129	131	131	128	130	128	123
Myanmar	NA	96	NA	NA	NA	NA	NA	127	124
Mali	116	115	112	114	114	122	124	124	125
Congo	129	126	125	128	129	131	127	123	126
Guinea	117	116	111	117	119	127	129	130	127
Chad	131	128	126	126	128	129	131	133	128
Sao Tome and Principe	95	61	138	92	97	100	133	84	129
United Republic of Tanzania	128	127	122	127	130	132	132	129	130
Liberia	NA	NA	127	130	133	NA	NA	134	131
Bangladesh	133	131	130	132	134	133	134	135	132
Gambia	134	132	131	133	135	134	136	136	133
Zambia	135	133	132	134	137	135	135	137	134
Uganda	137	136	135	136	139	139	137	138	135
Sudan	138	135	134	138	140	136	139	139	136
India	140	137	137	135	136	137	138	140	137
Ethiopia	136	134	133	137	138	138	140	141	138
Pakistan	139	138	136	139	141	140	141	142	139
San Marino	32	35	NA	NA	NA	NA	NA	29	NA
Grenada	NA	NA	NA	NA	NA	NA	NA	51	NA
Uzbekistan	NA	NA	NA	80	83	77	82	90	NA
Democratic Republic of the Congo	130	NA	NA	125	127	130	128	131	NA
Republic of Korea	NA	NA	NA	NA	NA	NA	24	NA	NA
Brunei Darussalam	38	27	29	NA	29	NA	29	NA	NA
Samoa	NA	NA	NA	NA	NA	NA	111	NA	NA
Luxembourg	NA	NA	3	NA	3	4	NA	NA	NA
Dominica	NA	NA	NA	NA	NA	57	NA	NA	NA
Angola	76	NA	NA	NA	NA	NA	NA	NA	NA
Solomon Islands	111	NA	NA	NA	NA	NA	NA	NA	NA

#### Regional Analysis

**Fig. 18:**
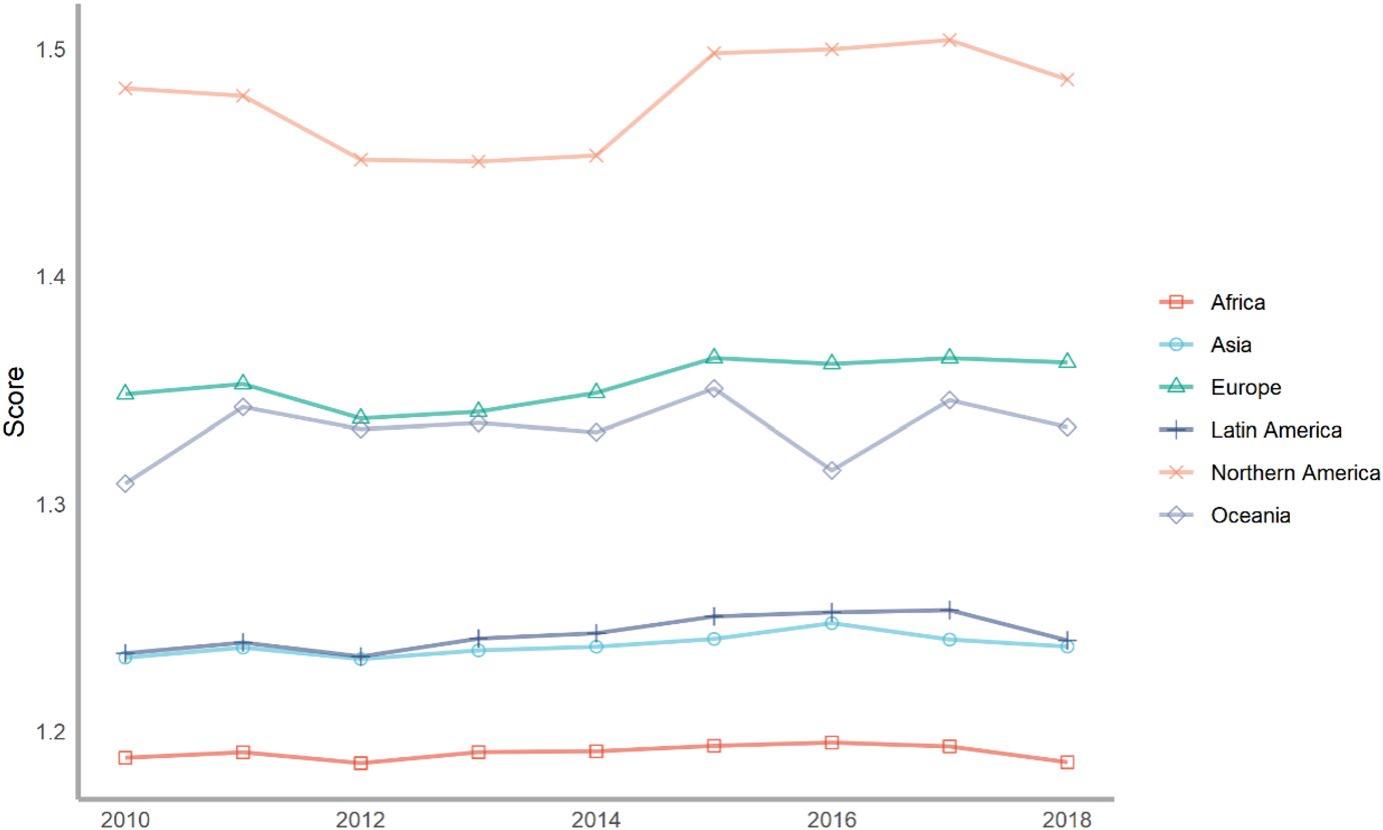
The score in the education issue area across continents, 2010–2018

Figure 18 shows the average score of each continent in the education issue area. The score clearly highly correlates to the level of economic development. First, as shown in Fig. 18, the score of North America, which is composed of the United States and Canada, in education is much better than that of the rest of the world. Second, Europe and Oceania also performed better; finally, Latin America, Asia and Africa performed poorly. In general, we found that all continents have been relatively stable in their education scores over the years.

However, it is worth noting that the rankings within continents vary greatly except in North America and Oceania. Generally speaking, North America, Western Europe, Australia and New Zealand and Northern Europe are in the first tier. And South Asia, West Africa, Central Asia, Central Africa, and North Africa rank low in the world.

**Asia** Similar to public health, the rank of Asia as a whole in the education issue area is only slightly higher than that of Africa, and within-continent variations exist. In Asia, for example, East Asia is second only to Northern Europe and does better than Southern and Eastern Europe. In particular, East Asia has an outstanding performance in the education performance dimension. For example, the scores of both China and Japan in the unweighted performance on education issue are among the best in the world, however, their scores in the contribution dimension are relatively low, which means that there is still a lot of room for improvement in their governments’ financial investment in education.^119^

Thanks to improvement in our model, eight countries including Norway, Iceland, Denmark, Switzerland, the United States, the Netherlands, Australia and Belgium were included in this year's report. These eight countries are ahead of China in education issues. As a result, China's ranking dropped from third in 2017 (as elaborated in in2019′s report) to 12th in 2018, as laid out in this year’s report. Even so, we believe that China has already made very impressive achievements in education issues, especially in the performance dimension. It should be pointed out that the ranking of education issues refers to a country’s contribution to global justice in basic education. The population-weighted performance, which is used to calculate global justice index, suggests that China ranks among the top in the world mainly due to two factors: the first is that China's performance on this issue exceeds the world average, meaning that the Chinese government provides its citizens with access to basic education that exceeds the world average. We compare China’s unweighted performance to that of other top 14 countries in education issue and find that China still scores high on unweighted performance.^120^ In fact, China is close to full marks on some indicators in basic education. For example, in 2018, China’s net enrollment rate of primary school and gross enrollment rate of lower secondary school reached 99.95%, and 100.9%, and the rate of trained teachers in primary and lower secondary school was 99.79% and 99.86%, respectively.^121^ Besides, the pupil-teacher ratio in both primary and lower secondary school has continued to decline since 2000.^122^ The second is China’s huge population size. In other words, China has allowed a large population to have access to basic education beyond the world average. Therefore, China’s good performance in education means that China has given a considerable proportion of the world’s population access to basic education opportunities that are better than the world’s average, but it does not mean that China has provided basic education opportunities for its citizens far beyond the rest of the world (including developed countries).

However, China’s score in contribution dimension is much worse than that in performance dimension. Although both China’s government expenditure on primary and lower secondary school and its share of GDP persistently increased,^123^ from the perspective of contribution, China's investment in basic education is much lower than the other 14 countries.

South and Central Asia do no better in education than most parts of Africa. In particular, South Asia’s rank is at the bottom globally. It is also worth noting that the three populous countries in South Asia, Bangladesh, India and Pakistan, are better than most African countries in terms of unweighted performance in education, but still far below the world average. In the population-weighted model, we believe that because these countries provide with educational opportunities below the world average, they contribute to global justice relatively less in terms of the education issue area.^124^

**Europe** Europe as a whole performed very well in education in 2018. Eight of the top 10 countries in the ranking of education issue come from Europe, namely Norway (1st), Iceland (2nd), Denmark (3rd), Switzerland (4th), Sweden (6th), Finland (7th), Netherlands (8th) and Belgium (10th). We compare the score (both unweighted and weighted) in the performance and contribution dimensions of the top 15 countries and find that there is only a small difference in the scores of performance dimension of the top countries except China, which suggests that these top countries perform well in the performance of education. The eight countries are all high-welfare countries. On the one hand, high investment improves their performance in basic education, therefore, increasing their scores in the (unweighted) performance dimension. Thanks to vast financial investment in basic education, on the other hand, the score of these countries in the contribution dimension are also very high.

**North America** North America as a whole is the best performer in the education issue area. First, it performs very well in the performance dimension. For example, out-of-school rates for children of primary school age and for adolescents of lower secondary school age in north America are 0.54% and 0.29% in 2018, respectively, much lower than 1.6% for children of primary school age and 2.29% for adolescents of lower secondary school age of high-income countries in the same year. The pupil–teacher ratio in primary education in north America is 14 in 2018, on par with high-income countries and second only to Europe area at 13. Second, North America as a whole invests much in public education. Government expenditure on education in North America accounted for 5.0% of GDP in 2014, on par with OECD countries and higher than 4.9% in Europe as a whole in 2017. Therefore, the United States of America and Canada, the two countries in North America, score highly in the dimension of both performance and contribution to education, high ranking 5th and 20th in 2018, respectively.

**Latin America** The rank of Latin America as a whole is very similar to that of Asia, but higher than that of Africa in period of 2010–18. In the past 20 years, Latin America has made significant progress in basic education. First, the expenditure of governments on education has steadily increased in recent years. Expenditure as a share of GDP in Latin America increased from 3.9% in 2000 to 5.6% in 2017—the highest of all continents—which is much higher than the global average (4.5%) in 2017. And expenditure as a share of total public expenditure in Latin America rose from 13.1% in 2002 to 16.5% in 2017, the highest of all continents.^125^ Three countries from Latin America are among the top 10 countries worldwide in terms of spending the highest proportion of GDP on education, namely Montserrat (8.3%), Belize (7.4%) and Costa Rica (7.0%).^126^ Second, the performance of public education also improved over the past 20 years, although the trend of improvement has slowed recently. Primary completion rate in Latin America as a whole slightly increased from 97.7% in 2000 to 98.15% in 2018. And net school enrollment in primary education fluctuated at around 94% from 2000–2018. Completion rates rose from 79 to 95% in primary school, from 59 to 81% in lower secondary school during this period.^127^ Meanwhile, the pupil-teacher ratio in Latin America decreased from 25.4 in 2000 to 21.3 in 2018.

It is also worth noting, however, that many social, cultural, economic and political factors, such as income inequality, social segregation, and colonial history are restricting the development of public education in Latin America.

**Africa** Africa as a whole has the lowest score in the education issue area in the world. In partciular, the unweighted performance of public education in sub-Saharan Africa was the worst in the world. In 2018, for instance, out-of-school rates were the highest in sub-Saharan Africa, at 19.2% of primary school age children and 37.1% of lower secondary school age adolescents. Out-of-school rates for children of primary school age and for adolescents of lower secondary school age in Southern Africa are 11% and 14.4% in 2018, respectively, which are similar to 7.2% and 15.5% in South Asia, a region which performs poorly based on our results.^128^ The gross enrolment ratio for primary and lower secondary education in sub-Saharan Africa in 2018 was only 73.6%. This was followed by South Asia with an 82.4% ratio.

Due to low fiscal revenue and GDP, sub-Saharan does not perform as poorly in terms of government expenditure on education relative to its GDP and government expenditure. Government expenditure on education in sub-Saharan Africa accounted for 4.3% of GDP, higher than the 3.5% in South Asia in 2017. And government expenditure on education accounted for 17.7% of government expenditure in 2018, the highest of all regions in the world.^129^

**Oceania** Oceania as a whole performed worse than North America and Europe but better than Latin America, Asia and Africa in terms of education. Australia and New Zealand ranked 6th and 14th in 2018. The two countries perform well in both the performance and contribution dimensions. First, for example, total net enrolment rates of primary school and of lower secondary school in Australia are 99.6% and 97.5% in 2018, respectively. And out-of-school rates for children of primary school age and for lower secondary school age are 0.4% and 2.5% in the same year. Second, Australian government expenditure on education accounted for 5.16% of GDP in 2014, higher than the USA’s 4.96% in 2014. According to our results, in fact, Australia and New Zealand as a whole performed worse than only North America and Western Europe, and better than the rest of the world.

#### Conclusion

Education is one of the fundamental elements of global justice. We use data from the World Bank to construct a country’s score in education for global justice from the perspectives of both performance and contribution. Our analysis shows a positive relationship between the education scores and economic development: the higher GDP per capita, the higher the scores in the education issue area. Countries with large populations have a special responsibility for global justice. However, since most of countries with large populations are underdeveloped, they perform poorly in education. We use a weighted linear regression (WLR) to estimate the correlation between the score in the education area issue and GDP per capita by weighting population size, and then find that many countries with GDP per capita of less than 5000 US dollar are below the fitting line of the WIR in the figure, suggesting that these countries performance in the education issue area is worse than expected.

We further find a weak correlation between unweighted performance scores in the education issue area and GDP per capita, especially when GDP per capita is higher than 10,000 US dollars. This may be because we only focus on basic education. There is a ceiling effect of basic education in terms of global justice, that is, when the economy develops to a certain level, the continued development of the economy does not necessarily improve opportunities for basic education.^130^ However, we also find a strong positive correlation between unweighted performance scores in education issue and GDP per capita in the countries with a GDP per capita less than 10, 000 US dollars. Moreover, we find that many African countries are below the lowess line, suggesting that they perform worse than expected.

Finally, we also examine the relationship between the scores of contribution and unweighted performance in education issue, and find that when the score of contribution reaches a certain point, the continued increase in the score of contribution may not necessarily lead to an improvement in (unweighted) performance in basic primary education. However, a high correlation between the score of contribution and unweighted performance of countries with low-score of contribution in education issue is found. The higher score of contribution in education, the higher the score of unweighted performance, which means that the appropriate investment would improve the performance of basic education. It is worth noting that the ceiling effect of the contribution score in education may be a result of the way in which we focus on the opportunities rather than the quality of basic education. More specifically, we just focus on completion rate, school enrollment, pupil-teacher ratio and out-of-school rate in both primary and secondary education. However, measures of the quality of education, such as the literacy rate or educational inequality within countries, are not included in this report because of lack of systematic data cross all countries for the years the report covers. In fact, a great deal of evidence suggests that the quality of basic education in high-income countries is better than that in low-income countries. If the quality of basic education highly correlates with GDP per capita as well as the contribution score of basic education, fortunately this lack of measurement of basic education quality would not lead to a bias in the rankings in the education issue area of global justice.

### Issue 9: Public Health

#### Introduction

The existence of health inequality in the world has become a consensus.^131^ For instance, there exist huge differences in life expectancy and mortality across countries. In Japan, Switzerland, Spain, France and other high-income countries, life expectancy at birth is more than 80 years, while in Lesotho, Sierra Leone, the Central African Republic, Chad and other low-income countries, life expectancy at birth was less than 60 years in 2018. In Iceland, Finland, Norway, Japan, Singapore, and other high-income countries, the under-five mortality rate is less than three in 1000 in 2018. But in other countries, children die at high rate. For example, In Guinea, Sierra Leone, the Central African Republic, Chad, Somalia and Nigeria, more than 100 out of 1000 children will die before they are 5 years old according to the 2018 data.^132^

Equitable access to comprehensive, effective health care systems is seen as a fundamental human right and a public good.^133^ A population in good health is essential for a prosperous economy and a stable society and can only be achieved through proper disease prevention and intervention. Providing adequate public health goods to the public is seen as an obligation of government. Therefore, the government is usually one of the main providers of public health goods and interveners in public health.^134^ We agree with Ruger (2009) that both the responsibility and the obligation of different actors, such as local, national and global actors, should be considered when constructing a theory of global health justice.^135^ Since the objectives of this report are evaluating the contribution of a country to global justice, however, we then focus on a country’s efforts to provide equitable access to public health for its citizens rather than the role of global, national and local communities and institutions in global health justice. That is our main difference with Ruger (2018)’s provincial globalism which emphasizes that all local, national and global actors have responsibilities in reducing health inequalities. Without nations’ efforts, global health actors, including the World Health Organization, the World Bank and other United Nations organizations, the vast numbers of foundations, NGOs and other actors, are unable to sufficiently resolve global health problems.

If public health is divided into domestic and global public health, domestic public health must account for the vast majority of national responsibility.^136^ Thus, in this report, we focus on the effort of a country to provide domestic public health to its citizens. The connections between public health as a domestic public good and public health as a country’s contribution to the global public good is that when one country progresses in public health, it also improves the whole world’s public health.^137^ In other words, a country will improve global justice by providing public health services to its citizens.

#### Dimensions and Indicators

We measure the contribution of a country’s public health to global justice from two perspectives. The first is the performance of each country’s public health with respect to protection of an individual’s right to health. We pay attention to the performance of basic public health from the perspective of global justice. We further divide the performance of public health into three dimensions, namely life expectancy and mortality, public health infrastructure, and key diseases. Specifically, (1) we use life expectancy at birth and life expectancy at age 60 to proxy for life expectancy and use the infant mortality rate, neonatal mortality rate, under five mortality rate, and the adult mortality rate to proxy for mortality. (2) For public health infrastructure, we adopt an indicator of the population using at least basic sanitation services, population using at least basic drinking water services to measure public health infrastructure. The indicator of *Population practicing open defecation,* is not adopted this year since the rate of missing values of this indicator is disproportionately high in high-income countries. (3) We use four indicators to measure key diseases: treatment success rate of new TB cases, tuberculosis effective treatment coverage, raised fasting blood glucose and incidence of tuberculosis.

The second perspective assesses the contribution of each country’s government to public health. We adopt two indicators: current health expenditure per capita and domestic general government health expenditure per capita, to measure for a country’s effort to promote its citizens’ public health. These data are drawn from the WHO and cover 190 countries from 2010 to 2018.^138^ The details can be found in Table 20.

**Table 20:** Data on public health

Category	Dimension	Indicator	Data Source	Coverage
Performance	Life expectancy and mortality	Life expectancy at birth (years)	WHO	185–188 (2010–2018)
		Life expectancy at age 60 (years)		
		Neonatal mortality rate (per 1000 live births)		
		Infant mortality rate (probability of dying between birth and age 1 per 1000 live births)		
		Under five mortality rate (probability of dying by age 5 per 1000 live births)		
		Adult mortality rate (probability of dying between 15 and 60 years per 1000 population)		
	Public health infrastructure	Population using at least basic drinking-water services (%), total		
		Population using at least basic sanitation services (%), total		
	Key disease	Treatment success rate: new TB cases		
		Tuberculosis effective treatment coverage (%)		
		Incidence of tuberculosis (per 100,000 population per year)		
		Raised fasting blood glucose (≥ 7.0 mmol/L or on medication)		
Contribution	Expenditure	Current health expenditure (CHE) per capita in US$		
		Domestic general government health expenditure (GGHE-D) per capita in US$		

#### Results

Table 21 shows the ranking of countries the education issues area from 2010 to 2018. Taking 2018 as an example, the top 10 countries in the ranking are the USA, Norway, Germany, Iceland, France, Luxembourg, Sweden, Denmark, Japan, and Belgium, all of which are developed countries. The bottom ten countries are Sudan, Myanmar, Albania, Azerbaijan, Afghanistan, Armenia, Venezuela, Yemen, Nigeria and India, all of which are underdeveloped countries.

**Table 21:** Country ranking in the protection of public health

Country	2010	2011	2012	2013	2014	2015	2016	2017	2018
United States of America	11	12	9	9	2	1	1	1	1
Norway	1	1	1	1	1	2	2	2	2
Germany	4	7	7	5	6	6	6	6	3
Iceland	17	19	19	16	14	10	9	7	4
France	5	6	6	6	7	8	8	9	5
Luxembourg	2	2	2	3	3	3	3	3	6
Sweden	10	3	4	2	4	4	4	4	7
Denmark	3	5	5	4	5	5	5	5	8
Japan	6	4	3	7	8	7	7	8	9
Belgium	14	13	14	13	11	12	12	10	10
United Kingdom of Great Britain and Northern Ireland	7	9	8	8	9	9	10	11	11
Ireland	12	17	17	19	18	17	15	12	12
Australia	19	16	16	18	20	18	16	15	13
Finland	16	15	15	15	12	13	13	14	14
Netherlands	8	10	10	10	10	11	11	13	15
Canada	15	14	13	14	16	14	17	16	16
Austria	18	18	18	17	17	15	14	18	17
New Zealand	21	20	20	20	19	20	20	19	18
Italy	9	11	12	12	15	16	18	17	19
Switzerland	26	22	22	23	24	22	24	22	20
China	34	27	23	22	21	19	19	20	21
Brunei Darussalam	25	24	26	28	26	25	22	21	22
Nauru	24	25	25	27	27	24	21	23	23
Kuwait	33	30	28	32	29	30	34	25	24
Tuvalu	22	21	21	24	23	23	23	24	25
Spain	23	26	32	35	35	33	28	28	26
Cuba	28	33	33	34	28	26	26	26	27
Qatar	55	49	34	25	22	21	25	31	28
Czechia	30	31	30	31	33	31	33	32	29
Oman	41	40	39	37	32	28	32	30	30
Micronesia (Federated States of)	29	29	27	30	31	27	29	27	31
Israel	54	55	56	52	48	42	37	41	32
Kiribati	27	28	29	29	30	29	30	29	33
Argentina	73	64	50	45	41	34	49	40	34
Estonia	46	46	47	48	46	44	45	42	35
Solomon Islands	32	32	31	33	34	32	31	33	36
Botswana	94	84	79	71	63	67	71	35	37
Croatia	35	34	35	36	37	35	36	39	38
Papua New Guinea	43	41	38	38	36	45	42	34	39
Vanuatu	38	36	37	40	40	37	41	37	40
Slovakia	50	48	52	46	38	36	35	43	41
Slovenia	36	38	45	44	44	41	39	36	42
Marshall Islands	45	45	44	42	47	39	38	38	43
Costa Rica	57	63	62	65	64	55	54	49	44
United Arab Emirates	53	52	51	54	56	50	55	48	45
Sao Tome and Principe	62	59	58	53	54	53	52	45	46
Uruguay	86	83	74	67	62	58	58	56	47
Maldives	109	129	119	115	91	60	59	57	48
Malta	59	60	61	55	66	65	60	60	49
Samoa	40	39	40	41	39	38	40	44	50
Lesotho	65	53	53	56	51	49	56	50	51
Rwanda	64	61	57	66	57	54	51	46	52
Timor-Leste	39	35	36	39	43	40	43	47	53
Palau	60	65	55	58	53	52	44	58	54
Portugal	37	47	59	59	58	57	57	62	55
Andorra	87	87	91	84	82	79	78	69	56
Romania	44	56	54	49	49	51	53	55	57
Thailand	52	42	49	47	50	47	48	54	58
Mozambique	42	43	42	43	42	43	47	52	59
Poland	56	57	64	63	61	62	66	59	60
Republic of Korea	66	70	75	75	69	66	64	66	61
Bhutan	49	51	60	62	65	59	63	53	62
Tonga	48	54	43	57	45	46	46	51	63
Hungary	67	68	80	77	75	70	69	63	64
Turkey	47	44	46	50	52	48	50	61	65
Greece	31	37	41	60	73	73	67	68	66
Seychelles	82	72	65	64	72	68	61	64	67
Eswatini	63	66	68	70	70	71	72	67	68
Malawi	51	50	48	51	55	61	62	65	69
Colombia	61	58	63	68	67	69	74	72	70
Zambia	88	82	67	61	59	56	65	70	71
Bosnia and Herzegovina	69	71	72	74	74	72	75	71	72
Saudi Arabia	93	78	73	69	60	64	70	75	73
Djibouti	79	67	66	72	68	63	68	73	74
Chile	98	98	96	96	97	91	97	80	75
Lithuania	58	62	76	81	76	75	79	77	76
Singapore	138	137	129	117	110	94	93	92	77
Bolivia (Plurinational State of)	100	100	103	101	93	80	83	81	78
Fiji	77	94	93	100	95	90	90	78	79
Saint Vincent and the Grenadines	91	97	77	82	86	84	91	76	80
Belarus	75	91	84	94	94	98	77	79	81
Panama	72	81	97	91	87	82	73	84	82
Dominica	85	89	86	89	85	77	76	83	83
Belize	74	79	83	83	83	76	81	85	84
Peru	124	126	117	111	101	97	100	86	85
Republic of North Macedonia	101	95	95	88	92	87	96	87	86
Cabo Verde	81	76	71	76	78	81	87	89	87
Zimbabwe	131	146	144	135	108	115	107	90	88
Jamaica	96	96	104	99	106	93	94	82	89
Suriname	135	147	148	149	151	88	80	93	90
Algeria	76	74	69	78	71	78	85	95	91
Gambia	89	93	87	73	84	103	103	94	92
Bahrain	84	86	81	80	79	74	86	102	93
United Republic of Tanzania	105	102	94	90	90	95	82	91	94
Guyana	107	111	110	109	111	114	89	88	95
Russian Federation	78	75	70	79	77	83	92	99	96
Libya	71	99	88	87	89	89	99	100	97
El Salvador	97	88	99	86	88	85	88	101	98
Kazakhstan	80	77	82	92	81	96	102	105	99
Mali	155	150	136	103	105	102	108	103	100
Nicaragua	119	107	105	106	103	101	95	96	101
Latvia	95	85	98	97	98	99	104	107	102
Mongolia	83	92	90	102	100	106	101	104	103
Trinidad and Tobago	127	127	128	116	121	112	112	108	104
Madagascar	125	104	114	126	102	92	84	97	105
Bahamas	118	120	118	120	132	105	105	121	106
Ecuador	143	140	133	131	123	130	119	109	107
Gabon	92	101	101	95	107	107	98	106	108
Serbia	90	90	92	98	104	104	106	110	109
South Africa	128	124	120	127	125	122	123	113	110
Republic of Moldova	113	125	111	114	109	126	130	111	111
Malaysia	123	122	115	118	116	117	128	119	112
Tunisia	115	110	108	105	112	111	113	112	113
Brazil	121	123	127	122	119	124	127	120	114
Mexico	129	121	116	113	113	110	111	118	115
Saint Kitts and Nevis	147	154	149	138	135	137	117	114	116
Central African Republic	134	131	125	139	118	86	122	98	117
Burkina Faso	99	109	107	119	128	135	109	116	118
Namibia	116	128	137	133	133	139	126	123	119
Bulgaria	114	116	126	125	117	123	125	124	120
Jordan	68	73	78	85	80	119	120	126	121
Lebanon	148	142	124	130	124	121	121	115	122
Iran (Islamic Republic of)	160	161	158	154	127	118	118	127	123
Barbados	102	113	102	112	126	120	115	129	124
Kenya	126	130	132	134	130	125	124	125	125
Uganda	111	117	138	140	122	127	129	122	126
Cyprus	110	114	123	121	131	132	132	132	127
Burundi	108	106	106	104	138	140	116	128	128
Dominican Republic	142	138	139	137	136	133	142	131	129
Haiti	103	80	89	110	99	108	110	130	130
Saint Lucia	154	156	152	152	145	138	143	133	131
Viet Nam	133	139	131	123	134	134	114	117	132
Antigua and Barbuda	106	108	109	108	114	109	131	135	133
Congo	122	115	85	93	96	100	134	139	134
Grenada	139	144	150	151	140	145	137	141	135
Niger	159	165	165	157	158	150	162	136	136
Honduras	136	134	145	144	141	143	139	144	137
Uzbekistan	132	133	130	128	120	129	141	140	138
Benin	137	136	112	136	139	128	136	137	139
Paraguay	140	135	141	146	137	136	138	138	140
Mauritius	141	145	142	147	143	142	144	142	141
Ukraine	120	132	121	132	147	148	146	143	142
Ghana	112	112	122	124	142	113	140	147	143
Sri Lanka	151	152	151	143	144	141	145	146	144
Kyrgyzstan	117	119	113	129	152	146	150	151	145
Lao People's Democratic Republic	158	149	143	141	146	131	135	134	146
Mauritania	163	164	156	145	148	144	152	150	147
Liberia	146	141	135	142	149	149	147	149	148
Indonesia	176	177	171	167	165	159	149	145	149
Angola	104	103	100	107	129	147	148	148	150
Eritrea	144	105	146	148	150	153	153	157	151
Morocco	156	155	153	150	155	155	151	152	152
Georgia	173	185	181	172	168	158	156	156	153
Senegal	153	157	161	165	153	154	154	160	154
Guatemala	157	159	157	158	154	157	159	155	155
Democratic Republic of the Congo	149	153	154	156	157	152	155	159	156
Tajikistan	172	160	164	160	160	160	166	158	157
Iraq	70	69	160	155	163	176	178	154	158
Cote d'Ivoire	180	181	168	174	166	163	160	153	159
Ethiopia	152	163	155	159	171	164	164	162	160
Cambodia	165	176	159	162	156	156	158	161	161
Nepal	167	171	169	171	169	166	167	163	162
Sierra Leone	161	170	177	163	115	116	133	167	163
Philippines	162	173	173	170	172	165	165	164	164
Chad	174	166	166	166	161	161	163	165	165
Egypt	171	169	176	175	173	171	168	166	166
Togo	168	167	162	161	162	162	157	168	167
Equatorial Guinea	170	162	134	178	179	178	174	171	168
Guinea	164	158	163	164	170	168	161	169	169
Turkmenistan	175	175	172	168	174	173	180	170	170
Guinea-Bissau	130	118	167	173	164	170	171	174	171
Pakistan	183	180	175	177	177	175	173	172	172
Cameroon	182	151	186	184	184	180	179	178	173
Comoros	184	172	185	182	178	174	170	173	174
Bangladesh	169	168	170	169	175	172	172	175	175
Sudan	166	174	174	185	176	167	175	179	176
Myanmar	185	186	182	176	167	169	176	177	177
Albania	177	178	180	183	183	177	177	176	178
Azerbaijan	181	182	179	181	180	179	183	181	179
Afghanistan	186	183	183	179	181	182	181	180	180
Armenia	179	184	184	186	185	181	182	183	181
Venezuela (Bolivarian Republic of)	150	143	140	153	159	151	169	182	182
Yemen	178	179	178	180	182	183	184	184	183
Nigeria	187	187	187	187	186	184	185	185	184
India	188	188	188	188	187	185	186	186	185
South Sudan	NA	NA	NA	NA	NA	NA	NA	74	NA
Cook Islands	20	23	24	26	25	NA	27	NA	NA
Niue	13	8	11	11	13	NA	NA	NA	NA
Monaco	NA	NA	NA	21	NA	NA	NA	NA	NA
Syrian Arab Republic	145	148	147	NA	NA	NA	NA	NA	NA

#### Regional Analysis

**Fig. 19:**
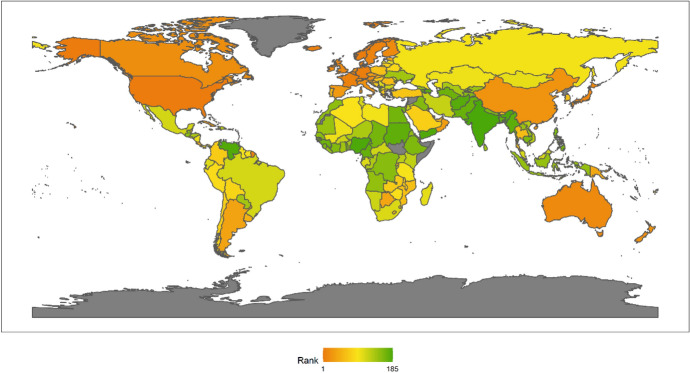
The score of public health issue across continents, 2010–2018

**Fig. 20:**
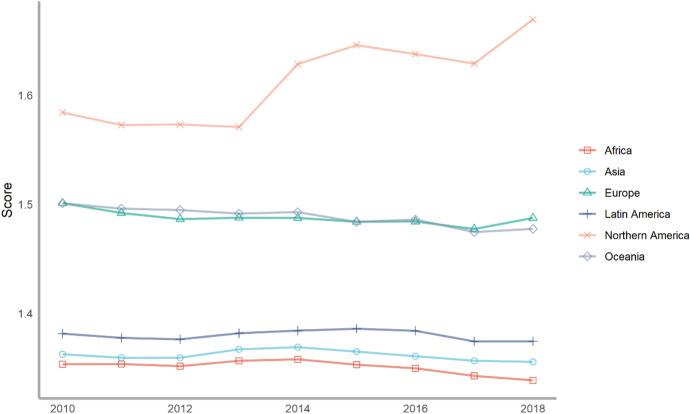
Index ranking of public health on a world map, 2018

As shown in Fig. 19, North America as a whole is far ahead of the rest of the world in term of the rank in public health. Both Europe and Oceania also perform very well. Africa is at the bottom of the rankings. The ranks of Asia and Latin America are slightly higher than that of Africa. It is worth noting that the rankings vary widely within continents. Specifically, North America, Western Europe, Australia and New Zealand, and Northern Europe make the top contribution to global justice in public health, while Southern Asia, Western Africa, Central Asia, Central Africa and Northern Africa are at the bottom (Fig. 20).

**Asia** The rank of Asia as a whole is only slightly higher than that of Africa, but lower than that of the rest of the world. However, as shown in Fig. 18, the rankings of Asian countries also vary widely. Specifically, countries in East Asia perform well in the issue of public health. For example, the ranks of Japan and China—9th and 21st, respectively—are higher than many high-income countries, including Spain, Portugal and Israel. Japan’s all-cause mortality rate was the lowest in the OECD in 2017 while its life expectancy at birth reached approximately 84.2 years in 2017, the highest in the OECD.^139^ Like other developing countries, China did not score well in the contribution dimension, ranking 106th among 185 countries in 2018. However, China ranked first in the performance dimension of public health in 2018, which is partly due to its large population size and its performance above the world average. In other words, although by developed countries’ standards, China’s government does not invest much into public health, it still provides a significant portion of population in the world with access to public health services that are above the world average.

South Asia is one of the worst regions in the public health issue area. South Asia's three most populous countries, namely India, Pakistan and Bangladesh, ranked last, third and fourth from the bottom in public health, respectively. This is partly due to their low government investment, their poor performance and large population size.

For example, India’s health expenditure accounted for rough 3.5% of its GDP, less than the world average (9.84%). India’s domestic general government health expenditure accounted for only 0.96% of GDP, less than 1/6 of the world’s average (5.87%). And its domestic general government health expenditure accounted for approximately 30% of health expenditure, which is also less than the world’s average (59.5%).

**Europe** Europe as a whole performed very well in public health in 2018. Eight of the top 10 countries in the ranking of public health are in Europe, specifically Western and Northern Europe, namely, Norway, Germany, Iceland, France, Luxembourg, Sweden, Denmark and Belgium. It is worth noting that these countries perform very well on the performance dimension of public health in spite of their small population size, suggesting that they provide a high quality of public health service to their people. For example, the life expectancy at birth of the eight countries reached 82.7 (Norway),81.1 (Germany), 82.2 (Iceland), 82.6 (France), 82.2 (Luxembourg), 82.5 (Sweden), 81.2 (Denmark) and 81.6 years (Belgium), all of which are higher than the OECD average (80.7 years) in 2017, while all-cause mortality rates (defined as number of deaths per 1000,000 people) of the eight countries is 701 (Norway), 777 (Germany), 725 (Iceland), 678 (France), 659 (Luxembourg), 710 (Sweden), 799 (Denmark) and 741 (Belgium), all of which is lower than the OECD average (801 deaths per 1000,000 people) in 2017.^140^

Another characteristic of these countries in public health is that they score highly in the contribution dimension of public health, suggesting that by the standards of the global average, these countries invest much into public health. It is estimated that health expenditure per capita of the government in the eight countries in 2018 are much higher than the average of the OECD countries.^141^

It is also worth noting that, however, the rankings of European countries also vary widely. As shows in Fig. 18, Eastern Europe performed worse than the rest of Europe, but still better than many countries from Africa, Asia and Latin America.

**North America** North America as a whole was far ahead of the rest of the world in the public health rankings in 2018. The United States and Canada ranked 1st and 16th in the world in 2018, respectively. The USA ranked 1st not only the overall issue area but also in the contribution dimension of public health. First, The USA’s domestic general government health expenditure per capita in 2018 reached approximately 5323 US dollars, the highest among all of the countries we observed. By one estimate, in 2018 the US spent 16.9% of its GDP on health care, which was approximately twice as much as OECD countries’ expenditure (8.8% of GDP). The US’s spending on health care is much higher than that of the other top 10 countries, such as Norway (10.2%), Germany (11.2%), Iceland (8.3%), France (11.2%), Luxembourg (5.4%), Sweden (11%), Denmark (10.5%), Japan (10.9%) and Belgium (10.4%).^142^ Second, public health spending on health in the USA during 2013–2016 accounted for 8.3% of total national GDP, which is less than some high-income countries, such as Sweden (10%), Netherlands (9.5%), Denmark (9.2%), France (8.7%) and Japan (8.6%), but higher than other, for instance, Switzerland (7.7%), UK (7.6%), Canada (7.4%) and Australia (6.3%).^143^ Third, the private health spending in USA was also higher than that in OECD countries. It is estimated that the private health spending in the USA accounted for 50.9% of total health spending in 2016, higher than the OECD average (25%).^144^ Although the USA ranks 1st in the OECD for health care expenditure, its score in performance dimension is lower than the other OECD countries.^145^ In 2016, for example, life expectancy in the total population at birth in USA was 78.8 years, lower than that in Japan (83.9), Switzerland (83), Australia (82.5), France (82.4), Netherlands (81.6), UK (81), Denmark (80.8) and Germany (80.7). Maternal mortality is 26.4 deaths per 100,000 live births, which is much higher than other high-income countries, such as the UK (9.2), Germany (9), France (7.8), Canada (7.3), Netherlands (6.7), Japan (6.4), Switzerland (5.8), Australia (5.5), Sweden (4.4) and Denmark (4.2). Moreover, the infant mortality rate of 5.8 deaths per 1000 live births in the USA is also higher than that in the other high-income countries we mention above.^146^

**Latin America** As shown in Fig. 3.9.2, Latin America as a whole performs better than Asia and Africa. However, countries within Latin America varied widely in terms of public health in 2018. For example, Argentina and Cuba ranked 34th and 27th, respectively, while Venezuela ranked 182nd among 185 countries in 2018. It is estimated that life expectancy at birth for the whole population across Latin America reached 74.5 years on average in 2017. However, large within-continent variations existed. The countries with the longest life expectancy in 2017 were Costa Rica and Chile at just over 80 years, which very closed to the OECD average (80.7 years). In contrast, life expectancy at birth in Haiti, Guyana and Bolivia is less than 70 years. In Haiti, life expectancy at birth was only 63.6 years in 2017.^147^

Large within-continent variations also existed in health expenditure by governments. For instance, in 2017, general government health expenditure in Cuba accounted for 10.5% of GDP, which was higher than the 6.6% in OECD countries. And in Argentina and Uruguay, general government health expenditure reached 6.6% of GDP, which was higher than the average (3.76%) for Latin America. However, in Venezuela and Haiti, general government health expenditure only accounted for 0.2% and 1% of GDP in 2017. This largely explains why the two countries ranked so low in terms of public health.

**Africa** Africa as a whole was the worst performer in public health. In both the performance and contribution dimensions of public health Africa scored poorly. For example, in 2016, there were at least 11 African countries with a life expectancy at birth below 60 years. And in 36 African countries, a child born in 2016 can expect to live an average of less than 65 years.^148^ The life expectancy at birth for Nigeria, which ranks 184th among 185 countries, in 2018 was only 54.3 years, which is less than that of India (69.1 years); and the infant mortality rate for Nigeria in 2018 was 75.7 deaths per 1000 live births, far more than that for India (29.7 deaths per 1000 births). Government health expenditure as a share of GDP is also low in Africa. For example, in 2016, the countries with the highest shares were Namibia (5.65%), Eswatini (5.33%), and Lesotho (5.15%), all of which are lower than the average (6.6%) of OECD countries. And in 2016, there were at least 26 countries with a share below 2% of GDP.

**Oceania** Oceania as a whole performed very well in terms of public health in 2018, however, the rankings of Oceanian countries in public health also vary widely. The two largest countries in Oceania, Australia and New Zealand, performed very well in the public health rankings in 2018. According to our ranking, Australia and New Zealand ranked 13th and 18th, respectively. However, other countries from Melanesia, Micronesia, and Polynesia did not perform so well. For example, life expectancies in Papua New Guinea and Fiji are only 64.3 and 67.3 years, respectively, which is far lower than the average (70.0 years) of life expectancy in lower-middle- and low-income countries in the Asia Pacific region. Infant mortality rates in Papua New Guinea and Fiji in 2018 are 38 and 21.6 deaths per 1000 live births, which very close to the average (27.2 deaths per 1000 live births) in lower-middle- and low-income countries in Asia Pacific region. However, these countries from Melanesia, Micronesia and Polynesia invest more into public health than those from Southeast and South Asia. For example, in Solomon Islands and Papua New Guinea, more than 75% of all health expenditure was paid for through government schemes and compulsory health insurance in 2017. By contrast, in some Southeast and South Asian countries, such as Myanmar, Bangladesh and Cambodia, less than 25% of health spending was through these schemes.^149^ Therefore, we observe that Oceania as a whole perform better than Asia in terms of public health.

#### Conclusion

Public health is one of the fundamental elements of global justice. We use data from the WHO to construct a country’s score of public health for global justice from the perspectives of both the performance and contribution dimensions. We examine the relationship between the public health score and economic development, measured as GDP per capita. We use a weighted linear regression (WLR) to estimate the correlation between the public health score and GDP per capita by weighting by population size. The fitting line of the WLR reveals a high correlation between a country’s contribution of public health to global justice and its economic development. Although these high-income countries perform well in the contribution of public health to global justice, the richest countries, such as Switzerland, Norway, Ireland, Iceland, Qatar and Singapore are below the fitting line. In addition, Venezuela and India are also far below the fitting line. This suggests that by the standard of economic development, these counties do less well than expected.

Based on a WLR, we also to find a high correlation between the score of contribution in public health issue and GDP per capita, suggesting that high-income countries as a whole invest much in public health. Again, by the standard of economic development, the richest countries, such as Switzerland, Norway, Ireland, Iceland, Qatar and Singapore, do not invest as much as expected. It is worth noting that, however, these countries performed very well in the performance dimension of public health, suggesting that unlimited financial expenditure on public health is not a sufficient and necessary conditions of good performance. Besides, many African countries are above the fitting line of the WLR of contribution on GDP per capita, suggesting that given their low GDP per capita, these countries invest more in public health than expected. However, unfortunately, when we use a fitting line of a WLR of unweighted performance on contribution and in public health to, we find that many African countries are below the fitting line of the WLR. A disproportionate relationship between the scores of contribution dimension and unweighted performance in many African countries, suggesting inefficiency in these countries’ investment in public health.

Last but not least, we find a weak correlation between the unweighted performance score in public health issue and GDP per capita. This may be because we only focus on basic public health. There is a ceiling effect of basic public health in terms of global justice, that is, when the economy develops to a certain level (e.g., when GDP per capita exceeds 10,000 US dollars as shown in this figure), the continued development of the economy does not necessarily improve its opportunities for basic public health. When we focus on underdeveloped countries with a GDP per capita of less than 10,000 UD dollars, we find a highly positive correlation between unweighted performance score in the public health issue area and GDP per capita, suggesting that when the higher the GDP per capita in underdeveloped countries, the better performance in public health, and that many African countries score of unweighted performance in public health is very low.

### Issue 10: Protection of Women and Children

#### Introduction

Protection of women and children is an important part of human rights treaties and it is essential for the achievement of global justice. There are various rankings relating to this issue across the world. For example, the Gender Inequality Index (GII) of the United Nations Development Programme measures gender inequalities in three important aspects of human development—reproductive health, empowerment and economic status. The Social Institutions and Gender Index (SIGI) of the OECD Development Center measures discrimination against women by taking into account laws, social norms and practices. The Kidsrights Index of the KidsRights Foundation is the first and only global ranking that annually measures how children’s rights are protected and to what extent countries are committed to improving the rights of children. At the regional level, there is the Gender Equality Index measuring the progress of gender equality in the EU. Most of these indexes measure the protection of women and children from the perspective of human rights. Considering that our research focuses on the contributions of different states to global justice, we use a population-based weighed score of indices to construct our scores on this issue from the perspective of global justice. In this way, our index is distinguished from most of the existing indexes and fills a gap in the measurement of protection of women and children from the perspective of global justice.

#### Dimensions and Indicators

The basic framework of indicators remains the same as our measurement last year. It is difficult to distinguish a country’s financial contribution to the protection of women and children from other issues such as public health, education, and poverty. However, a country’s performance on this issue is clear and measurable. As a result, for this issue area we focus only on the performance dimension. First, we use the ratio of health, demography, economic status, and political empowerment between males and females to measure gender inequality from the perspective of gender-based gaps in resources and opportunities in countries. Second, we focus on the gender difference of children’s situations from the perspective of poverty, health and education.

Table 22 shows the detailed information about the indicators we use. Data on children’s health and demography is obtained from the WHO, and the remainder are obtained from the World Bank.

**Table 22:** Indicators of the protection of women and children

Category	Dimensions	Indicators	Data Source	Coverage
Performance (Women)	Health & Demography	Life expectancy at birth, ratio female to male(years)	World Bank	155–159 (2010–2018)
		Maternal mortality ratio female to male (modeled estimate, per 100,000 live births)		
		Number of under-five death of thousands, female		
		Sex ratio at birth (male to female births)		
	Economic status	Unemployment, female (% of female labor force)		
		Vulnerable employment, ratio female to male		
		Wage and salaried workers, ratio female to male		
	Political status	Proportion of seats held by women in national parliaments (%)		
Performance (Children)	Children health and demography	Number of deaths per 1000 + (include 13 indicators)	WHO	
		Prevalence of thinness among children and adolescents, BMI < -2 standard deviations below the median (crude estimate) (%)		
	Children education (The Educational Difference between males and females)	School enrollment, primary (gross), gender parity index (GPI)	World Bank	

#### Results

According to the results, China remained in first place in the protection of women and children from 2010 to 2018 (Table 23). However, it t is worth noting that we used a population-based weighted score of indices to construct the scores for this issue from the perspective of global justice. As a result, the score here indicates not the level of protection for women and children in respect of an individual country’s situation, but the country’s total contribution in improving women and children’s living situation as a whole. Specifically, we set up a base line, and if a country performed better than the base line, the more women and children in this country, the higher the score it gets. However, if a country performed worse than the base line, the more women and children in this country, the lower the score is. This explains why China ranked the first while India, a country with a large population, ranked 157th in 2018.

**Table 23:** Country ranking in the protection of women and children

Country	2010	2011	2012	2013	2014	2015	2016	2017	2018
China	1	1	1	1	1	1	1	1	1
United States of America	2	2	2	2	2	2	2	2	2
Russian Federation	3	3	3	4	4	4	3	3	3
Brazil	4	4	4	3	3	3	4	4	4
Germany	5	5	5	6	6	6	6	5	5
Mexico	9	9	8	9	8	7	8	6	6
United Kingdom of Great Britain and Northern Ireland	6	6	7	7	7	8	7	7	7
France	7	7	6	8	9	9	9	8	8
Italy	8	8	9	10	10	10	10	9	9
Republic of Korea	10	11	10	12	11	11	11	10	10
Thailand	11	10	11	11	12	12	12	11	11
Poland	16	14	14	14	15	14	13	12	12
Spain	12	12	13	15	16	16	16	13	13
Ukraine	18	18	16	17	13	15	14	14	14
Argentina	14	13	12	13	14	13	15	15	15
Canada	17	15	15	16	17	17	17	16	16
Viet Nam	15	16	17	18	19	18	18	17	17
Australia	19	19	19	20	20	20	19	18	18
Saudi Arabia	28	26	24	21	22	22	22	20	19
Colombia	27	25	25	23	21	21	20	19	20
Philippines	13	17	18	19	18	19	21	21	21
Netherlands	22	21	21	25	24	24	24	22	22
Romania	26	28	27	30	28	27	25	23	23
Syrian Arab Republic	32	23	23	22	23	23	23	24	24
Kazakhstan	33	32	29	29	27	25	26	25	25
Malaysia	20	20	20	24	25	26	28	26	26
Belarus	25	27	28	28	29	29	27	27	27
Belgium	29	29	30	31	30	31	29	28	28
Sweden	30	30	31	32	32	32	31	29	29
Cuba	24	24	26	27	31	30	30	30	30
Czechia	31	31	32	33	33	33	32	31	31
Portugal	35	33	34	36	35	35	33	32	32
Chile	34	35	36	34	34	34	34	33	33
Uzbekistan	46	43	40	37	37	38	38	34	34
Sri Lanka	37	36	33	35	36	36	36	35	35
Turkey	138	127	115	108	52	37	41	40	36
Hungary	36	37	38	39	38	39	37	37	37
Dominican Republic	49	47	42	43	42	45	44	36	38
Austria	38	38	39	41	39	40	39	41	39
Israel	40	39	41	42	43	41	40	38	40
Peru	39	40	47	56	46	43	42	39	41
Lebanon	54	52	52	49	49	49	45	44	42
Bulgaria	47	48	48	48	50	48	47	43	43
Finland	43	41	43	44	45	46	46	45	44
Denmark	44	45	46	46	48	50	49	46	45
Norway	45	44	45	47	47	51	51	48	46
Switzerland	42	42	44	45	44	47	48	47	47
Tunisia	56	56	55	50	51	52	53	49	48
Ireland	51	51	53	52	56	54	54	50	49
Slovakia	50	50	51	51	54	55	55	52	50
Serbia	53	54	54	54	55	53	52	51	51
Venezuela (Bolivarian Republic of)	23	22	22	26	26	28	35	42	52
Greece	41	46	49	55	57	57	60	54	53
Kyrgyzstan	57	57	58	60	59	59	58	53	54
Kuwait	61	61	59	59	60	60	59	55	55
New Zealand	52	53	56	57	58	58	57	56	56
Costa Rica	55	55	57	58	61	62	61	57	57
Republic of Moldova	58	58	61	61	62	61	63	58	58
Croatia	59	60	63	63	64	64	65	60	59
Lithuania	60	62	60	62	65	63	64	59	60
Tajikistan	64	68	65	64	69	68	66	61	61
Qatar	70	72	72	67	70	69	69	64	62
Uruguay	62	67	66	65	68	67	67	63	63
Panama	65	64	67	66	66	66	68	65	64
Ecuador	48	49	50	53	53	56	62	62	65
Mongolia	68	71	70	69	71	71	72	66	66
Slovenia	63	66	68	68	72	73	71	68	67
Georgia	72	73	74	73	75	74	70	69	68
Nicaragua	69	63	64	70	74	70	75	72	69
Latvia	67	70	71	71	73	72	74	71	70
El Salvador	66	69	69	74	67	65	73	70	71
Estonia	71	73	73	75	76	76	76	73	72
Albania	84	81	80	81	80	79	78	74	73
Bolivia (Plurinational State of)	114	114	109	112	101	86	77	75	74
Republic of North Macedonia	78	76	76	80	81	82	80	78	75
Trinidad and Tobago	75	79	77	77	78	81	79	77	76
Cyprus	74	74	75	78	82	85	82	79	77
Bahrain	79	80	79	80	83	84	84	81	78
Mauritius	77	78	78	79	84	83	83	80	79
Armenia	102	101	99	104	93	87	85	82	80
Paraguay	76	77	81	72	77	75	81	83	81
Montenegro	81	83	82	82	87	88	86	84	82
Malta	83	84	83	83	88	88	87	85	83
Luxembourg	80	82	84	84	86	89	88	86	84
Iceland	82	85	85	85	89	90	89	87	85
Bahamas	85	86	86	86	90	91	91	88	86
Suriname	88	89	88	87	92	92	92	89	87
Barbados	86	88	87	88	91	93	93	90	88
Cabo Verde	97	99	97	97	104	101	98	91	89
Brunei Darussalam	NA	NA	NA	NA	NA	NA	94	92	90
Belize	96	97	95	94	100	97	99	93	91
Saint Lucia	91	92	91	90	94	94	95	94	92
Samoa	90	93	92	91	95	95	96	95	93
Saint Vincent and the Grenadines	94	94	93	92	97	98	97	96	94
Tonga	93	95	94	93	99	99	100	97	95
Fiji	87	91	90	89	96	96	101	98	96
Solomon Islands	92	96	96	95	102	102	102	100	97
Vanuatu	95	98	98	96	103	103	103	101	98
Sao Tome and Principe	98	100	100	100	105	104	104	102	99
Maldives	99	103	102	102	106	105	105	104	100
Guyana	100	102	101	103	107	106	106	103	101
Democratic People's Republic of Korea	NA	NA	NA	NA	NA	80	NA	NA	102
Algeria	109	59	35	40	40	44	56	76	103
Honduras	73	65	62	76	79	78	90	99	104
Bhutan	101	104	103	105	108	107	108	106	105
Oman	89	87	89	98	98	100	107	105	106
Azerbaijan	105	90	105	107	110	113	110	108	107
Timor-Leste	103	105	104	106	109	108	109	107	108
Iran (Islamic Republic of)	131	124	119	99	63	77	116	67	109
Senegal	128	128	124	125	124	123	125	121	110
Djibouti	107	107	107	110	113	111	111	109	111
Comoros	106	106	106	109	112	110	112	110	112
Botswana	108	108	108	111	115	112	114	111	113
Cambodia	110	110	110	113	114	109	113	112	114
Eswatini	118	113	112	115	116	115	115	113	115
Namibia	112	112	113	116	118	117	118	114	116
Lesotho	119	117	116	118	121	120	120	117	117
Rwanda	117	118	118	119	119	116	117	116	118
Equatorial Guinea	111	109	111	114	117	118	119	115	119
Gambia	115	115	114	117	120	119	121	118	120
Guatemala	104	75	117	101	111	114	122	119	121
Lao People's Democratic Republic	120	120	121	120	123	122	123	120	122
Myanmar	139	139	139	137	135	133	133	132	123
Mauritania	121	119	120	121	122	121	124	122	124
Papua New Guinea	122	122	125	122	126	124	126	123	125
Congo	123	121	123	123	125	125	127	124	126
Burundi	127	130	129	127	129	127	129	125	127
Togo	126	125	127	126	128	126	130	126	128
Liberia	125	123	126	124	127	128	128	127	129
Madagascar	132	132	130	129	130	129	131	128	130
Central African Republic	130	131	131	130	131	132	132	129	131
Morocco	129	126	128	128	132	130	135	130	132
Nepal	124	129	132	131	133	131	134	131	133
Malawi	135	134	133	134	134	134	136	133	134
Sierra Leone	136	136	136	135	136	135	137	135	135
Zambia	137	137	137	138	138	136	138	134	136
Uganda	142	142	141	141	141	139	140	137	137
Niger	143	140	140	140	142	142	143	140	138
Benin	133	133	135	136	137	137	139	136	139
Burkina Faso	141	138	146	142	144	141	145	142	140
South Africa	152	147	143	144	143	140	142	139	141
Guinea	134	135	138	139	139	138	141	138	142
Ghana	140	141	142	143	145	143	146	141	143
Indonesia	21	34	122	132	140	144	144	143	144
Chad	145	143	144	145	146	145	147	145	145
United Republic of Tanzania	146	146	148	150	150	146	148	144	146
Cameroon	149	148	149	148	148	148	149	146	147
Mali	147	145	145	146	147	147	150	147	148
Afghanistan	151	150	151	149	151	151	151	149	149
Mozambique	148	149	150	151	152	152	153	150	150
Yemen	144	144	147	147	149	149	152	148	151
Bangladesh	155	155	155	155	154	154	157	151	152
Kenya	153	152	152	152	153	153	155	153	153
Egypt	156	154	154	154	155	156	154	154	154
Ethiopia	154	153	153	153	156	155	156	152	155
Democratic Republic of the Congo	157	156	156	156	157	157	158	155	156
India	160	159	159	158	159	159	159	156	157
Pakistan	158	157	157	157	158	158	160	157	158
Nigeria	159	158	158	159	160	160	161	158	159
Guinea-Bissau	116	NA	NA	NA	NA	NA	NA	NA	NA
Eritrea	113	116	NA	NA	NA	NA	NA	NA	NA
Angola	150	151	NA	NA	NA	150	NA	NA	NA
Japan	NA	NA	NA	5	5	5	5	NA	NA
Zimbabwe	NA	NA	134	133	NA	NA	NA	NA	NA
Singapore	NA	NA	NA	NA	NA	NA	50	NA	NA
Gabon	NA	111	NA	NA	NA	NA	NA	NA	NA
United Arab Emirates	NA	NA	37	38	41	42	43	NA	NA
Turkmenistan	NA	NA	NA	NA	85	NA	NA	NA	NA

The United States remained in second place in this issue from 2010 to 2018. Countries with a large population who performed better than the base line ranked at the forefront as well, such as Russia, Brazil, and Mexico. Those countries with a large population but performed worse than the base line ranked at the bottom, such as Indonesia, India and Pakistan. Traditional democratic countries in Europe also ranked highly, including Germany, the UK, France and Italy.

**Fig. 21:**
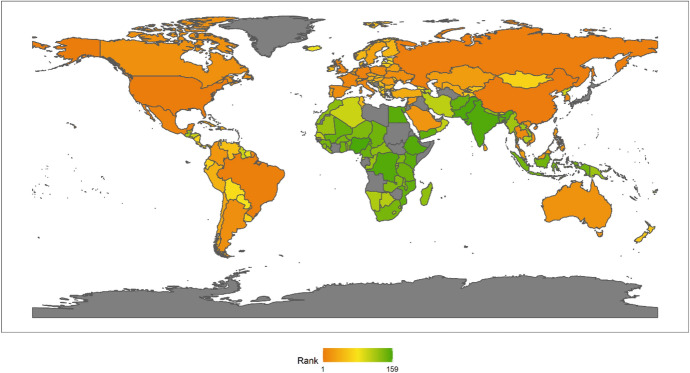
2018 Index ranking of the protection of women and children on a world map

The top ten countries in 2018 were China, the United States, Russia, Brazil, Germany, Mexico, the United Kingdom, France, Italy and Korea (Fig. 21). As we discussed earlier, a higher score does not denote a higher level of protection for women and children from an individual perspective, but rather means a higher improvement in conditions across all women and children. Thus, the fact that China ranked first reflected that China has made many women and children far better off than the world average.

#### Regional Analysis

See Fig. 22.

**Fig. 22:**
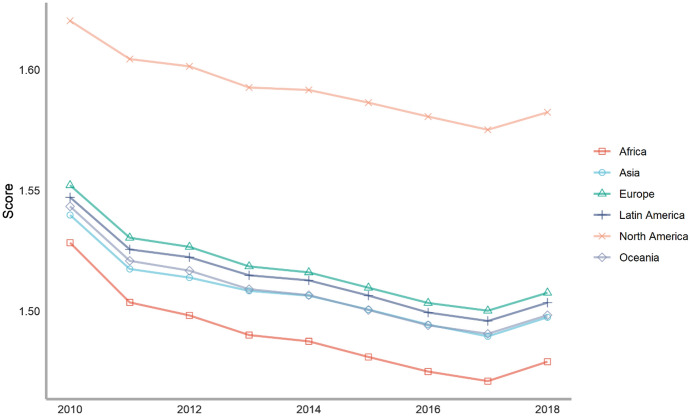
The score of Protection of Women and Children across continents, 2010–2018

**Asia** According to our result, East Asian countries such as China, Korea and Japan contributed greatly on this issue. At the regional level, ASEAN, as mentioned above in the section on transnational organized crime also plays a role in the protection of women and children. All of the ASEAN member states have ratified to the Convention on the Elimination of all Forms of Discrimination against Women (CEDAW) and the Convention on the Rights of the Child (CRC).

China ranked the first under our measurement of this issue. The Chinese government issued a series of policy papers, including national laws, local regulations and administrative rules, to protect women’s rights and interests. Taking women’s employment rights as an example, the 1992 Law on the Protection of Rights and Interests of Women is the main piece of legislation regarding the protection of women in the workplace. Additionally, the Constitutional Principle (1954), the Regulation Governing Labour Protection for Female Staff and Workers (1988),the Labour Law (1994), the Law on the Protection of Women's Rights and Interests (1992, revised 2005), the Employment Promotion Law (2008) and the Employment Contract Law (2008) provide legal protections to women in the workplace.^150^ In regard to the protection of children, China has also established a legal framework to protect children’s interests and rights. The framework is composed of conventions of the UN, international organizations and NGOs, laws issued by the National People’s Congress and its Standing Committee, administrative regulations of the State Council and local rules and relevant regulations.

**Europe** Gender equality and protection of children are foundational values under the EU treaties and are common topics in EU meetings. The Council of Europe has established a Committee for Equality between Women and Men, and the European Court of Human Rights also plays a role in the protection of women and children protection at the judicial level. For example, the European Court in different judgments has held the governments of Belgium, Denmark, Ireland, the Netherlands, Switzerland and the UK to be in violation of their duties to respect the human rights of women.^151^ In 2018, the EU adopted a new strategic approach to women, peace and security. In the Council conclusions, it emphasized the importance of full implementation of the Women, Peace and Security (WPS) agenda by all its member states and affirmed that the implementation of the EU Strategic Approach to WPS should be achieved through political and diplomatic engagement by the EU leadership. There are a large number of associations and organizations protecting the rights and interests of women and children. For example, the European Women’s Lobby, which aimed to “exert pressure on European and national institutions to ensure better defense and representation of women’s interests”.^152^

**North America** In North America, violence towards women and children is a widely focused social issue. The US government has established a legal framework against gender-based violence and violence towards children over the past several decades. President Clinton signed the Violence against Women Act (VAWA) into law in 1994. VAWA represents significant progress in the legal protection against gender-based violence. It emphasizes a coordinated community response to different types of violence including domestic violence, dating violence, sexual assault, stalking, etc. The Act also granted 1.6 billion dollars towards the investigation and prosecution of violent crimes directed towards women. Additionally, VAWA established the Office on Violence against Women within the Department of Justice. In Canada, Canada’s Strategy to Prevent and Address Gender-Based Violence (the Strategy) was issued in 2017 and acts as the main regulation against gender-based violence. The Strategy’s initiatives are organized across three pillars: preventing gender-based violence, supporting survivors and their families, and promoting responsive legal and justice systems. Additionally, the Canadian government provided over 200 million dollars (over 40 dollars per year) for the establishment and implementation of the Strategy.^153^

**Latin America** Latin America is a region with relatively serious problems of inequality, discrimination and violence towards women and children. According to data from the UNDP, more than 14 countries in Latin America and the Caribbean have categorized femicide as a crime. In Brazil, Chile, Bolivia, Colombia, Ecuador, Peru, Costa Rica, El Salvador, Guatemala, Honduras, Mexico, Nicaragua, Panama and Dominica, laws have been passed against femicide and other forms of violence against women. Antigua and Barbuda, Argentina, Bahamas, Barbados, Belize, Bolivia, Brazil, Chile, Colombia, Costa Rica, Dominica, Dominican Republic, Ecuador, El Salvador, Grenada, Guatemala, Guyana, Haiti, Honduras, Jamaica, Mexico, Nicaragua, Panama, Paraguay, Peru, Saint Kitts and Nevis, St. Lucia, St. Vincent and the Grenadines, Suriname, Trinidad y Tobago, Uruguay and Venezuela are signatory countries to the Inter-American Convention on the Prevention, Punishment and Eradication of Violence Against Women. However, to achieve a comprehensive realization of human rights protection there are still serious challenges to overcome.^154^

**Africa** Gender-based violence and violence towards children are severe problems in Africa due to a complex combination of conditions including hunger, poverty, conflicts and war. According to data from the World Economic Forum, 137 women around the world are killed by a family member each day, and 52 of them are in Africa. To improve women’s empowerment in Africa, the World Bank initiated the Sahel Women's Empowerment and Demographic Dividend project at the demand of the governments of Benin, Burkina Faso, Chad, Cote d’Ivoire, Mali, Mauritania and Niger. The project helped women to access to opportunities for education, employment, and engagement in policymaking. In regard to the protection of children, the United Nations Convention on the Rights of the Child (CRC) and the African Charter on the Rights and Welfare of the Child (ACRWC) are the main legal instruments to protect children from various problems including violence, child labor and military use of children. Article 19 of the CRC and Article 16 of the ACRWC require that member states should take all appropriate measures to prevent children from violence, maltreatment and abuse.^155^

**Oceania** Over the last several years, the Australian government has issued a series of policies and regulations to protect women from various forms of violence, such as the National Plan to Reduce Violence against Women and their Children 2010–2022 by the Council of Australian Governments in 2010 and The Time for Action: The National Council’s Plan for Australia to Reduce Violence Against Women and Their Children 2009–2021 by the National Council. Additionally, the National Plan has led to the establishment of two organizations relating to violence against women: Australia’s National Organisation for Women’s Safety and the National Foundation to Prevent Violence against Women and their Children. The former focuses on the promotion of national research activities to solve relevant problems, and the latter endeavors to induce changes culture, behavior and power imbalances. Strategies and policies have also been released at the territory level to promote gender equality and prevent violence. For example, the Achieving Women’s Equality was released in 2015 by the South Australian Government, and the Queensland Women’s Strategy 2016–2021 put forward by the Queensland Government in 2016.^156^

#### Conclusion

The protection of women and children, as a component of fundamental human rights, has long been an important part of the achievement of global justice. Legal frameworks and judicial systems have been established in most countries to protect women’s rights to education, employment and engagement in policy-making as well as protecting children from all forms of violence, maltreatment and abuse. However, there are still important challenges to overcome to achieve a comprehensive protection of women and children. We measured the performance and contribution of each nation state in this issue across 11 indicators in all. Eight of these, including life expectancy, maternal mortality ratio, death ratio, sex ratio, unemployment, vulnerable employment, wages and salaries, and proportion of seats held by women in national parliaments, were used to measure the protection of women, while three (number of deaths per 1000 + , prevalence of thinness among children and adolescents, and school enrollment) were used to measure the protection of children. According to the results, the top ten countries in 2018 are China, the United States, Russia, Brazil, Germany, Mexico, the United Kingdom, France, Italy and Korea. However, as we discussed earlier, we use a population-based weighed score of indices to construct score for this issue from the perspective of global justice. As a result, the highest score indicates not the best performance at the individual right level, but rather means that the country has made a sufficient number of women and children far better off than the world average.

## Global Justice Indices: Main Results

In this section, we report each country’s contribution to global justice from 2010 to 2018. Data availability is one of the most serious challenges facing this study. For example, because of the lack of data on energy consumption and electricity production, the issue of climate change in this report only covers 75 countries in 2018. Similarly, data on the issue areas of education and anti-poverty only cover 139 and 152 countries in 2018, respectively. Most of the countries which are not covered in these three issues are in Asia and Africa. Thus, we first provide a global justice index that excludes climate change and anti-poverty over 2010–2018 (Table 24); second, a global justice index over 2010–2018 that excludes climate change and education is reported (Table 25). Finally, we provide a global justice index of all ten issues over 2010–2018 (Table 26).

**Table 24:** Global Justice Index (except for both climate change and anti-poverty)

Country	2010	2011	2012	2013	2014	2015	2016	2017	2018
United States of America	1	1	1	1	1	1	1	1	1
Germany	3	3	3	4	3	3	2	2	2
United Kingdom of Great Britain and Northern Ireland	2	2	2	2	2	2	3	3	3
Sweden	6	5	5	5	6	6	7	5	4
Norway	5	6	6	7	7	7	6	6	5
China	4	4	4	6	5	5	5	4	6
Canada	7	7	7	8	9	9	8	7	7
Belgium	14	13	11	13	13	13	11	10	8
Italy	8	8	8	9	8	8	9	8	9
Finland	11	12	9	11	10	12	12	12	10
Switzerland	12	11	10	12	12	11	13	11	11
Denmark	9	10	12	10	11	10	10	9	12
Netherlands	13	14	13	14	14	14	14	14	13
Austria	16	16	18	18	18	19	17	16	14
Spain	10	9	14	15	15	15	15	13	15
Australia	15	15	17	17	20	20	19	17	16
New Zealand	17	18	19	19	21	21	20	19	17
Ireland	18	19	21	21	23	23	21	20	18
Saudi Arabia	29	40	37	38	30	45	36	33	19
Brazil	19	17	15	16	16	17	16	15	20
Russian Federation	23	23	22	22	19	18	18	18	21
Argentina	20	21	20	20	22	22	22	21	22
Israel	22	27	28	27	24	26	24	22	23
Portugal	21	24	24	23	26	28	26	23	24
Iceland	67	66	68	69	63	53	47	34	25
Rwanda	49	46	31	39	36	31	33	30	26
Chile	24	26	25	25	29	27	27	26	27
Ethiopia	71	22	36	34	39	35	28	25	28
Mexico	36	34	34	28	35	34	29	28	29
Senegal	38	33	32	30	27	24	23	24	30
Uruguay	27	29	27	24	28	32	31	31	31
Czechia	33	28	38	31	32	33	32	32	32
South Africa	28	32	33	35	38	40	39	47	33
Ghana	31	61	29	40	34	36	40	35	34
Malta	32	37	49	43	53	39	38	36	35
Zambia	43	48	45	51	50	49	48	39	36
Togo	47	35	30	32	25	30	30	37	37
Philippines	25	31	26	29	31	37	35	27	38
Peru	57	53	52	54	43	44	43	40	39
United Republic of Tanzania	34	45	39	33	41	42	34	29	40
Indonesia	81	83	77	77	77	67	60	59	41
Malawi	40	38	23	41	40	29	42	41	42
Poland	26	30	35	37	42	41	45	38	43
Costa Rica	64	41	41	49	48	50	51	42	44
Qatar	39	36	48	36	37	38	46	48	45
Belarus	52	57	55	57	79	56	54	46	46
Romania	54	52	56	52	52	54	55	52	47
Malaysia	59	59	64	64	75	79	76	72	48
Lesotho	51	44	40	50	49	63	52	50	49
Lithuania	53	54	63	58	57	64	61	55	50
India	30	25	44	26	33	43	44	43	51
Mozambique	41	43	46	44	46	52	50	45	52
Thailand	48	47	51	45	47	51	53	53	53
Paraguay	61	58	58	67	54	60	63	61	54
Hungary	62	62	66	66	76	69	75	64	55
Burkina Faso	68	56	65	47	56	47	41	51	56
Latvia	65	63	67	60	67	72	70	58	57
Panama	44	42	43	46	45	48	49	49	58
Nepal	35	20	73	65	64	46	66	60	59
Guatemala	73	51	50	56	51	57	64	57	60
Slovakia	55	55	59	59	59	61	62	63	61
Estonia	69	74	75	82	81	76	69	62	62
Kenya	37	39	47	53	58	59	59	68	63
El Salvador	66	72	62	75	65	62	67	65	64
Colombia	60	67	70	79	87	83	79	75	65
Slovenia	50	49	61	63	74	78	73	70	66
Ukraine	58	60	53	42	44	55	71	69	67
Cyprus	63	65	82	84	85	81	78	71	68
Cameroon	92	71	106	109	89	77	87	81	69
Bangladesh	42	50	42	48	60	65	68	54	70
Uganda	56	64	57	55	55	58	56	44	71
Liberia	NA	NA	69	62	73	NA	NA	66	72
Sierra Leone	100	92	92	93	62	68	57	67	73
Serbia	70	68	71	70	80	82	77	76	74
Tunisia	75	76	81	81	84	84	80	79	75
Djibouti	45	70	60	61	72	70	74	78	76
Croatia	74	80	86	87	93	91	88	84	77
Eswatini	96	95	76	86	98	92	83	80	78
Benin	93	75	72	76	78	73	72	74	79
Niger	98	96	90	73	69	66	58	56	80
Republic of Moldova	103	87	79	78	82	87	85	83	81
Ecuador	78	78	88	88	94	98	91	89	82
Nicaragua	85	82	83	71	71	74	82	82	83
Madagascar	76	79	84	74	68	71	65	77	84
Bulgaria	77	84	78	80	91	94	86	90	85
Fiji	106	103	102	113	103	114	112	88	86
Cuba	90	89	99	94	92	85	81	85	87
Pakistan	82	69	54	68	61	25	37	73	88
Honduras	NA	NA	NA	85	70	86	NA	NA	89
Mauritius	86	85	94	102	100	102	110	94	90
Timor-Leste	72	73	74	83	90	90	90	87	91
Lebanon	115	99	101	101	102	100	102	96	92
Armenia	105	91	91	95	95	95	94	93	93
Kyrgyzstan	87	77	80	90	99	101	98	100	94
Kazakhstan	79	88	93	96	97	99	97	99	95
Central African Republic	84	81	89	72	66	88	103	91	96
Mauritania	97	101	112	110	108	106	100	97	97
Mali	94	90	103	91	88	75	104	98	98
Cambodia	99	102	95	100	96	97	92	95	99
Chad	112	107	108	108	106	89	95	105	100
Georgia	119	108	111	112	113	104	106	102	101
Gambia	80	97	87	92	83	96	84	92	102
Bahrain	83	86	96	104	104	112	99	111	103
Azerbaijan	88	94	97	103	105	105	109	104	104
Burundi	109	109	100	99	107	109	101	103	105
Namibia	95	106	109	105	109	108	114	108	106
Tajikistan	101	100	105	107	114	113	113	109	107
Oman	91	98	98	98	101	103	105	101	108
Congo	113	113	115	115	110	110	107	106	109
Mongolia	108	111	104	106	112	111	111	107	110
Belize	104	105	110	114	117	107	116	114	111
Guyana	114	116	117	118	120	119	120	113	112
Albania	102	104	107	111	116	116	115	112	113
Barbados	111	110	113	117	115	115	117	115	114
Sri Lanka	117	114	114	119	119	118	118	117	115
Afghanistan	110	112	116	116	118	117	119	116	116
Viet Nam	116	117	119	122	123	122	122	119	117
Saint Vincent and the Grenadines	118	115	118	121	122	121	121	118	118
Maldives	121	120	122	125	126	123	124	121	119
Saint Lucia	123	121	123	124	125	125	126	123	120
Lao People's Democratic Republic	120	118	120	123	124	124	125	122	121
Bhutan	122	119	121	126	127	126	127	124	122
Myanmar	NA	122	NA	NA	NA	NA	NA	125	123
Iran (Islamic Republic of)	46	93	85	89	86	80	89	86	NA
Democratic Republic of the Congo	89	NA	NA	97	111	93	108	110	NA
Uzbekistan	NA	NA	NA	120	121	120	123	120	NA
Japan	NA	NA	NA	3	4	4	4	NA	NA
Republic of Korea	NA	NA	NA	NA	NA	NA	25	NA	NA
Brunei Darussalam	NA	NA	NA	NA	NA	NA	93	NA	NA
Samoa	NA	NA	NA	NA	NA	NA	96	NA	NA
Luxembourg	NA	NA	16	NA	17	16	NA	NA	NA

**Table 25:** Global justice index (except for both climate change and education)

Country	2010	2011	2012	2013	2014	2015	2016	2017	2018
United States of America	1	1	1	1	1	1	1	1	1
Germany	3	3	3	4	4	2	2	2	2
United Kingdom of Great Britain and Northern Ireland	2	2	2	2	2	3	3	4	3
China	5	4	4	5	5	5	5	3	4
Sweden	7	6	6	7	8	8	8	7	5
France	4	5	5	6	6	6	6	5	6
Canada	8	8	8	9	9	9	9	8	7
Italy	6	7	7	8	7	7	7	6	8
Belgium	15	12	12	11	13	11	12	10	9
Norway	9	10	9	10	10	10	10	9	10
Finland	11	11	11	13	11	13	13	12	11
Switzerland	12	13	14	14	16	17	18	13	12
Spain	10	9	10	12	12	12	11	11	13
Denmark	14	15	16	15	18	16	15	15	14
Netherlands	13	14	15	17	17	14	17	17	15
Austria	16	18	17	18	19	19	19	18	16
Luxembourg	18	20	19	22	21	21	20	19	17
Russian Federation	23	21	21	20	15	15	14	14	18
Australia	17	17	18	19	22	22	21	20	19
Greece	21	19	20	21	20	20	22	21	20
Ireland	19	22	22	23	23	24	24	22	21
Brazil	20	16	13	16	14	18	16	16	22
Portugal	24	25	25	27	25	26	25	24	23
Turkey	22	23	23	24	24	23	23	23	24
Republic of Korea	30	33	31	33	30	27	27	26	25
Israel	25	32	30	32	29	30	30	29	26
Chile	26	26	27	26	26	25	26	25	27
Uruguay	28	30	26	25	27	29	28	27	28
Czechia	29	27	29	28	28	28	29	28	29
Belarus	33	37	36	38	55	39	33	32	30
Ethiopia	89	29	47	39	48	45	34	31	31
Rwanda	56	61	35	44	43	38	43	43	32
Mexico	45	38	42	37	53	43	36	36	33
Poland	27	28	28	30	32	33	31	30	34
Malta	31	35	41	34	49	36	35	33	35
Zambia	36	46	39	48	45	47	42	38	36
Malaysia	49	52	53	54	56	60	61	59	37
Peru	52	56	49	52	38	42	38	37	38
Costa Rica	55	36	33	40	39	46	44	40	39
India	32	24	37	31	33	40	37	35	40
Iceland	84	82	88	89	87	77	76	68	41
Lithuania	40	47	51	51	44	51	47	44	42
Thailand	43	39	46	35	35	35	39	39	43
Romania	54	59	57	53	50	52	46	45	44
Hungary	47	49	54	57	57	53	62	52	45
Paraguay	53	55	50	61	40	50	49	51	46
Ghana	58	87	45	55	51	49	57	50	47
South Africa	44	41	44	45	47	54	52	64	48
Senegal	57	48	48	46	36	34	32	34	49
Latvia	50	53	60	56	58	58	56	47	50
Slovakia	37	40	40	50	42	48	45	49	51
Malawi	42	44	24	43	41	31	48	48	52
Panama	35	34	34	36	37	41	40	41	53
Togo	65	43	38	41	34	37	41	54	54
Ukraine	38	42	32	29	31	44	54	53	55
Philippines	48	50	43	49	46	57	55	42	56
Serbia	59	58	62	63	60	59	58	60	57
Egypt	51	54	69	70	72	79	68	57	58
Republic of North Macedonia	61	63	63	60	59	61	63	62	59
Indonesia	97	101	105	100	103	97	75	73	60
United Republic of Tanzania	46	62	59	42	52	56	50	46	61
Lesotho	63	57	56	62	63	72	60	58	62
Estonia	68	68	72	73	71	68	64	61	63
Slovenia	41	45	52	58	61	64	66	63	64
Mozambique	39	51	55	47	54	55	53	55	65
Croatia	60	66	64	67	69	69	71	67	66
El Salvador	75	79	68	82	70	63	67	66	67
Colombia	72	71	78	84	80	83	80	77	68
Republic of Moldova	98	91	79	71	67	73	83	70	69
Guatemala	81	65	61	68	62	66	70	65	70
Tunisia	78	75	75	79	74	80	78	75	71
Bulgaria	69	74	65	66	75	81	74	74	72
Cyprus	73	69	76	83	76	74	77	71	73
Bolivia (Plurinational State of)	62	60	58	59	66	78	72	81	74
Algeria	NA	NA	NA	76	78	75	79	78	75
Azerbaijan	64	70	71	72	77	76	82	76	76
Morocco	70	92	84	77	79	70	81	79	77
Ecuador	80	80	82	88	88	94	90	87	78
Lebanon	NA	NA	NA	86	84	82	89	84	79
Mauritius	83	83	87	93	91	92	95	85	80
Kazakhstan	77	77	80	74	81	85	87	86	81
Burkina Faso	85	78	86	65	83	67	59	72	82
Uganda	66	73	66	64	64	65	65	56	83
Madagascar	82	85	83	80	73	71	69	83	84
Cameroon	101	90	113	116	98	91	99	96	85
Nepal	71	31	103	101	99	84	96	93	86
Botswana	92	97	94	106	107	109	108	97	87
Liberia	90	76	89	87	93	100	91	88	88
Kenya	67	67	77	85	90	88	88	95	89
Honduras	87	86	90	95	85	95	93	89	90
Benin	100	89	85	90	89	86	86	91	91
Sierra Leone	108	102	107	111	94	90	84	92	92
Fiji	113	113	109	122	112	119	118	103	93
Armenia	119	114	108	108	109	105	103	98	94
Eswatini	104	108	93	99	110	108	100	100	95
Samoa	96	96	102	107	105	103	97	99	96
Bangladesh	74	81	74	75	92	93	92	82	97
Dominican Republic	88	95	96	103	104	101	94	101	98
Central African Republic	91	94	92	78	68	89	102	94	99
Pakistan	95	93	73	92	82	32	51	90	100
Nicaragua	99	100	98	97	96	98	98	102	101
Niger	103	107	104	96	95	87	85	80	102
Burundi	106	110	97	98	108	107	101	104	103
Georgia	122	120	117	119	117	111	109	109	104
Mongolia	109	115	100	102	102	104	107	105	105
Chad	115	117	115	114	114	102	106	111	106
Namibia	107	116	116	110	111	113	112	110	107
Albania	102	109	110	115	118	117	115	114	108
Papua New Guinea	NA	105	99	112	116	112	113	106	109
Democratic Republic of the Congo	86	99	91	94	106	96	105	107	110
Tajikistan	114	112	114	117	120	118	119	116	111
Mali	105	106	112	104	100	99	114	113	112
Mauritania	112	118	119	121	119	120	116	118	113
Timor-Leste	94	98	101	109	113	116	117	112	114
Gambia	93	104	95	105	97	110	104	108	115
Congo	116	122	120	120	115	114	111	117	116
Viet Nam	110	121	121	123	122	122	120	119	117
Sri Lanka	121	124	122	125	123	123	122	121	118
Kyrgyzstan	111	103	111	118	121	121	121	122	119
Nigeria	79	88	81	91	101	115	110	115	120
Tonga	120	123	123	126	125	124	123	123	121
Yemen	118	119	118	124	124	125	124	120	122
Maldives	124	126	125	128	126	126	125	124	123
Bhutan	125	127	126	129	128	128	127	125	124
Uzbekistan	123	125	124	127	127	127	126	126	125
Lao People's Democratic Republic	126	128	127	130	129	129	128	127	126
Myanmar	NA	NA	NA	NA	NA	NA	129	128	127
Iran (Islamic Republic of)	34	84	67	69	65	62	73	69	NA
Japan	NA	NA	NA	3	3	4	4	NA	NA
Angola	117	111	NA	NA	NA	106	NA	NA	NA
Venezuela (Bolivarian Republic of)	76	72	70	81	86	NA	NA	NA	NA
Zimbabwe	NA	NA	106	113	NA	NA	NA	NA	NA
Gabon	NA	64	NA	NA	NA	NA	NA	NA	NA

**Table 26:** Global justice index (including all ten issues)

Country	2010	2011	2012	2013	2014	2015	2016	2017	2018
United States of America	1	1	1	1	1	1	1	1	1
Germany	3	3	3	5	3	3	2	2	2
United Kingdom of Great Britain and Northern Ireland	2	2	2	2	2	2	3	4	3
China	4	4	4	4	4	4	4	3	4
Sweden	5	5	5	6	6	6	6	5	5
Norway	6	6	6	7	7	7	7	6	6
Canada	7	7	7	8	9	9	8	7	7
Belgium	14	13	12	13	13	13	13	12	8
Finland	11	10	9	10	10	10	11	9	9
Italy	8	8	8	9	8	8	9	8	10
Switzerland	12	11	10	12	12	12	12	11	11
Denmark	10	12	14	11	11	11	10	10	12
Austria	15	17	17	18	18	19	18	16	13
Netherlands	13	14	15	15	16	14	15	15	14
Spain	9	9	13	14	15	15	14	13	15
Australia	16	15	16	17	20	20	19	18	16
Ireland	17	18	20	20	21	21	20	19	17
Russian Federation	20	19	19	19	17	16	17	17	18
Brazil	18	16	11	16	14	17	16	14	19
Portugal	19	20	21	21	22	22	22	20	20
Israel	21	22	23	23	23	23	23	21	21
Iceland	38	38	39	41	38	30	27	24	22
Chile	22	21	22	22	24	24	24	22	23
Czechia	24	23	25	24	25	25	25	23	24
Poland	23	24	24	25	26	26	26	25	25
Mexico	30	27	28	28	33	29	28	28	26
Belarus	26	29	26	29	41	31	29	26	27
Malaysia	32	32	34	37	39	40	40	38	28
Peru	37	37	31	34	28	27	30	27	29
Lithuania	28	31	35	31	31	33	33	29	30
Latvia	35	33	36	32	35	35	35	30	31
Hungary	33	34	37	39	40	37	41	36	32
Thailand	29	30	32	27	29	28	31	31	33
Romania	40	41	40	38	37	36	34	33	34
Slovakia	27	28	29	33	30	32	32	34	35
Slovenia	25	26	27	35	36	38	36	35	36
Estonia	43	43	44	44	44	43	39	37	37
Cyprus	34	35	41	42	43	42	43	39	38
South Africa	39	39	38	40	42	44	42	43	39
Philippines	41	36	33	36	34	41	38	32	40
Indonesia	50	50	52	51	51	51	47	45	41
Ukraine	36	40	30	26	27	34	44	41	42
Colombia	44	42	45	45	46	46	46	42	43
India	42	25	42	30	32	39	37	40	44
Bulgaria	45	44	43	43	45	48	45	44	45
Ecuador	47	46	48	48	49	50	51	48	46
Azerbaijan	46	45	46	46	48	49	49	47	47
Kazakhstan	49	47	49	49	50	52	52	49	48
Bangladesh	48	49	50	50	52	53	53	50	49
Viet Nam	51	52	54	53	55	55	55	52	50
Pakistan	52	51	51	52	53	45	50	51	51
Sri Lanka	53	53	53	54	54	54	54	53	52
Iran (Islamic Republic of)	31	48	47	47	47	47	48	46	NA
Uzbekistan	NA	NA	NA	55	56	56	56	54	NA
Japan	NA	NA	NA	3	5	5	5	NA	NA
Republic of Korea	NA	NA	NA	NA	NA	NA	21	NA	NA
Luxembourg	NA	NA	18	NA	19	18	NA	NA	NA

**Fig. 23:**
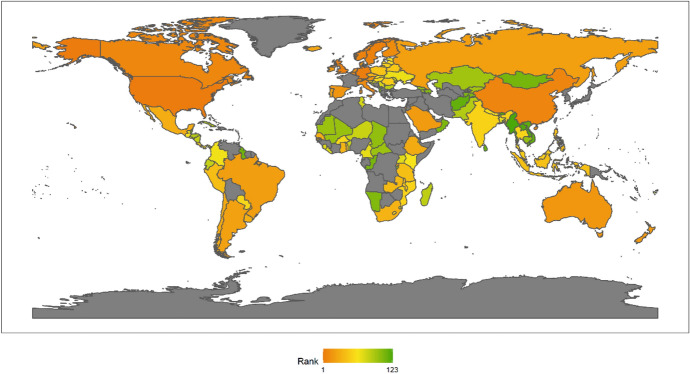
2018 Index ranking of global justice (except for climate change and anti-poverty)

Table 24 shows the global justice index excluding both climate change and anti-poverty. In 2018, as shown in the table, the United States, Germany, the United Kingdom, Sweden,Norway, China, Canada, Belgium, Italy and Finland rank as the top 10 in the global justice index that excludes climate change and anti-poverty. All of the top ten countries except China are are high-income and located in North America and Europe. Most countries which perform badly in global justice come from Africa, Asia and the Caribbean, including Myanmar, Bhutan, Lao People's Democratic Republic, Saint Lucia, Maldives. Afghanistan, and Vietnam, Congo, Sri Lanka and Barbados. Figure 23 vividly shows the distribution of the rank of global justice (except for climate change and anti-poverty) in 2018. As the figure shows, North America, Europe and oceania rank highly, while Africa and Asia (especially South Asia and West Asia) rank low.

**Fig. 24:**
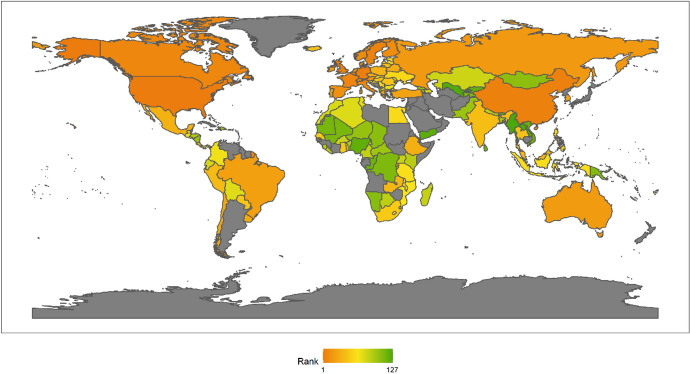
2018 Index ranking of global justice (except for climate change and education)

Table 25 shows the global justice index except for both climate change and education. Similarly to Table 24, Table 25 shows that, in 2018, the United States, Germany, the United Kingdom, China, Sweden, Fracen, Canada, Italy, Belgium and Norway rank among the top 10 in the global justice index (excluding chimate change and education). Myanmar, Lao People's Democratic Republic, Uzbekistan, Bhutan, Maldives, Yemen, Tonga, Nigeria, Kyrgyzstan and Sri Lanka, all of which come from Africa, Asia and Oceania, are the bottom ten countries in the global justice index. Figure 24 clearly shows the indistribution of the rank in global justice (excluding climate change and education) in 2018. Although the two figures exclude different issues, Fig. 24 shows the same pattern as Fig. 23.

**Fig. 25:**
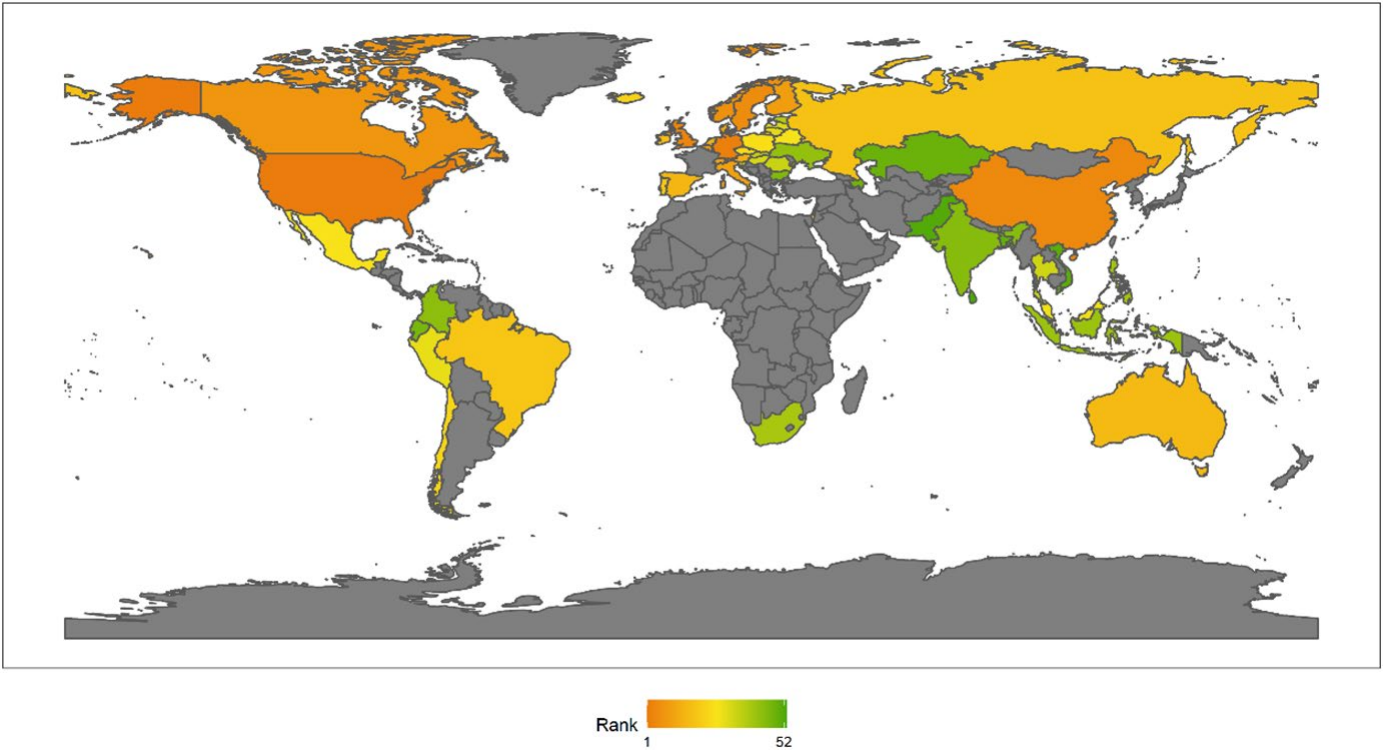
2018 Index ranking of global justice (including all ten issues)

Table 26 shows the global justice index including all ten issues. In 2018, as shown in Table 26, the United States, Germany, the United Kingdom, China, Sweden, Norway, Canada, Belgium, Finland and Italy ranked the top ten in the global justice index that includes all issues. Although Table 26 only covers 52 countries in 2018, the results are very similar to Tables 24 and 25, suggesting the robustness of global justice index. In addition, Fig. 25 also illustrates that the lowest-ranked countries come from Africa, Asia, and Latin America, and that, apart from China, the highest-ranked countries come from Europe and North America, affirming that the rank of global justice index highly correlates with economic development.

**Fig. 26:**
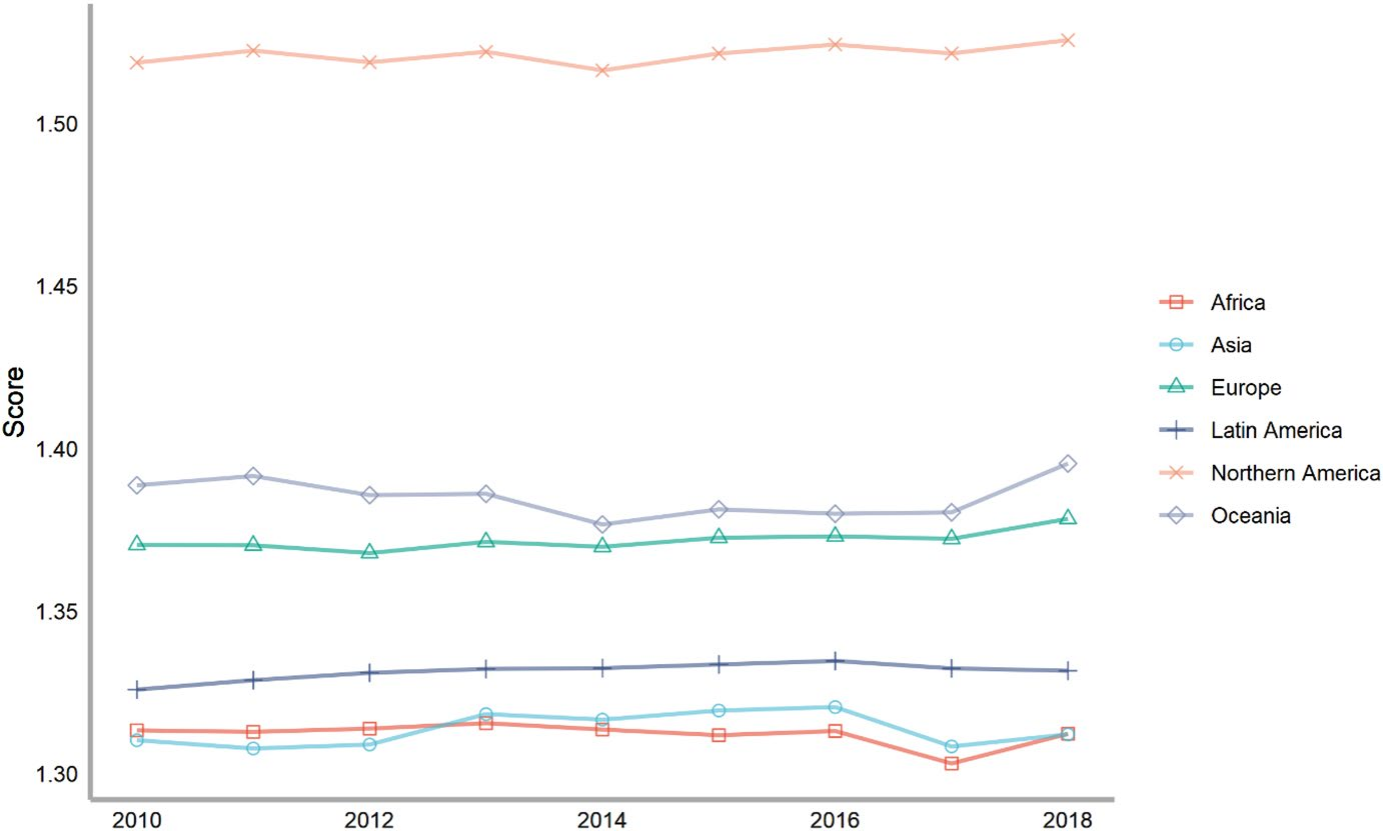
The score of global justice index (including all ten issues) across continents, 2010–2018

We also present the scores in the global justice index (including all ten issues) across continents from 2010 to 2018 in Fig. 26. The figure shows that North America, Europe and Oceania ranked highly while Latin America, Asia and Africa ranked low from 2010 to 2018, and that the pattern has been stable over the years, suggesting the robustness of our measurement. Even so, we remind readers of the variation within continents. For example, although Asia as a whole ranked low during 2010–2018, two coutries from Asia,China and Japan, performed well: China ranked 4th in 2018 and Japan ranked 6th in 2016. And vice versa, although Europe as a whole ranked highly, Bulgaria and Ukraine, both of which are in Europe, only ranked 45th and 42nd out of 52 countries in 2018.

## Conclusion

The Global Justice Index is a multiyear research project to conceptualize and measure each country’s contribution to global justice. In this year’s Global Justice Index, we kept unchanged the theoretical framework of last year and made a few modifications to refine our measurements. According to the two major principles of CBDR-RC and CDDR, which was the synthesis of rights based, goods based, and virtue based approaches embedded in the historical discussion of global justice, we added a brand new issue area into our measurement and ended up with a ten-issue index system: (1) climate change (global warming), (2) peacekeeping, (3) humanitarian aid, (4) terrorism and armed conflicts, (5) cross-national criminal police cooperation, (6) refugee, (7) anti-poverty, (8) education, (9) public health, and (10) the protection of women and children. Additionally, we have improved our data collection and strengthened the analysis section with more policy-oriented discussion.

Our results show that the United States, Germany, the United Kingdom, China, Sweden, Norway, Canada, Belgium, Finland and Italy are the top ten countries in 2018 in their contribution to global justice. The United States ranks 1st and China ranks the highest among developing countries. In our measurement last year, the final result covered merely 2010 to 2014 due to data limitations, and the top five countries were the United States, the United Kingdom, Germany, China and France. Through improving the methodology and complementing missing data, we expanded the coverage to 2018 in this year’s report and the result shows little change, which suggests the robustness of our global justice index.

This year’s global justice index is our second release of the research result of the Global Justice Project which was initiated in 2018. The index is designed to empirically measure the performance and contribution of nation-states to enhancing justice at the global level by considering ten different issue areas. The following findings of this project have a number of implications: (1) the index provides the first comprehensive assessment of global justice development, which can be used by the international and regional communities to trace and monitor individual countries’ performance, therefore pushing relevant policymakers to develop targeted interventions aimed at enhancing global justice; (2) the index establishes a quantitative framework for detecting changes and weakness in different issue areas of global justice. This information will be of assistance to country leaders and local practitioners to set policy priorities and invest continued efforts; (3) despite its exploratory nature, this index offers some insight into methodological approaches to measuring various justice-related variables, which will be of broad use to academic communities for conducting further causal analysis.

Several limitations to this index need to be acknowledged: (1) because no existing literature has provided solid evidence about how to determine the weight of the ten issues to global justice, we, therefore, assume that the ten issues equally contribute to global justice. However, in reality, a country’s equal efforts in different issue areas may affect the ranking result to different degrees; (2) another limitation of using this index is that due to data availability problems, we were not able to include all nation states in the index. For some issue areas, such as poverty, climate change and education, this problem is particularly serious. This means that the ranking presented in the index may better be understood as a relative ranking, and the results may change as the data improves; (3) another caveat which needs to be noted regarding conducting comparisons is that a comparison of the global justice index ranking across the observation years and across different issue areas is inappropriate because each year/issue area in fact contains different numbers of countries due to the problem of missing data. This means that the ranking results need to be interpreted cautiously; (4) another source of weakness lies in the possibility of measurement bias. The measurement of some issue areas seems relatively simple. Although we have designed and collected a comprehensive set of indicators, not all the indicators have sufficient and reliable data to secure accurate measurement. In addition, we rely more on objective indicators. For example, on education, we have considered the school enrollment rate, but the measurement is limited by the lack of information on education quality. All these limitations, on the one hand, remind us to be prudent in interpreting the index results and on the other hand indicate that there is abundant room for further work.

## Footnotes

1 Crowley (2000).

2 Johnston & Deeming (2016).

3 India: The Impact of Climate Change to 2030. https://www.dni.gov/files/documents/climate2030_india.pdf.

4 Adger et al. (2011).

5 Kingston (2013).

6 Verhoeven, I. (2020). Contentious governance around climate change measures in the Netherlands. *Environmental Politics*, online first.

7 Selby (2019).

8 Turubanova et al. (2018).

9 Collier et al. (2008).

10 Mills‐Novoa & Liverman (2019).

11 Lipson (2007).

12 Bara & Hultman (2020).

13 https://www.passblue.com/2019/02/12/why-developing-nations-send-so-many-troops-to-un-peace-ops/.

14 https://news.un.org/en/gallery/541602.

15 Hang (2013).

16 Hamman & Omojuwa (2013).

17 Philippine Participation in UN Peace Operations. https://www.fsi.gov.ph/wp-content/uploads/2019/08/2015-Special-Issue-No-2-May-FSI-Insights-UN-Peace-Operations.pdf.

18 For more information about Séléka, see Kah (2013).

19 Zaman, & Biswas, (2014).

20 Coleman & Nyblade (2018).

21 Kathman, & Melin, (2017).

22 https://www.deutschland.de/zh-hans/topic/zhengzhi/weihepinggongtongnuli.

23 Davis (2011).

24 Zarka et al. (2019).

25 Please check https://fts.unocha.org/content/about-fts-1 for more information about FTS.

26 https://www.wfp.org/overview.

27 https://www.wfp.org/types-of-support.

28 Chin & Frolic (2007).

29 Dreher et al. (2017).

30 Helen Versluys (2008).

31 https://www.state.gov/policy-issues/refugee-and-humanitarian-assistance/#:~:text=Total%20U.S.%20humanitarian%20assistance%20worldwide,%2C%20Conflict%2C%20and%20Humanitarian%20Assistance.

32 https://ec.europa.eu/echo/where/latin-america-caribbean/south-america_en.

33 https://ec.europa.eu/echo/where/latin-america-caribbean/south-america_en.

34 https://ec.europa.eu/echo/where/africa_en.

35 Bogg (2015).

36 Taylor (2019).

37 https://world.huanqiu.com/article/9CaKrnKbsL7.

38 http://www.xinhuanet.com/globe/2019-06/06/c_138094065.htm.

39 Coolsaet (2010).

40 Appleton (2014).

41 Taylor (2019).

42 Hoffman (1995).

43 Heine & Thakur (2011).

44 Broude & Teichman (2009).

45 Andreas & Nadelmann (2008).

46 Broude & Teichman (2009).

47 https://www.unodc.org/unodc/en/organized-crime/intro/UNTOC.html.

48 United Nations Convention against Transnational Organized Crime and the Protocols Thereto (2004), United Nations, p.9.

49 Please check https://www.interpol.int/en for more information about Interpol.

50 https://www.unodc.org/unodc/en/organized-crime/technical-assistance.html

51 AEI WORKING GROUP ON TRANSNATIONAL ORGANIZED CRIME IN THE AMERICAS. (2017). Kingpins and Corruption: TARGETING TRANSNATIONAL ORGANIZED CRIME IN THE AMERICAS (pp. 5–10, Rep.). American Enterprise Institute. Retrieved January 30, 2021, from http://www.jstor.org/stable/resrep03288.4.

52 Foertsch. (1999).

53 Europol Convention. P. 6.

54 United Nations Office on Drugs and Crime (2019), Transnational Organized Crime in Southeast Asia: Evolution, Growth and Impact.

55 Ralf Emmers (2003).

56 Saskia Hufnagel (2014).

57 Finckenauer & Chin (2006).

58 Woodward, Susan L(2004).

59 Velkova & Saso (2004).

60 Velkova & Saso (2004).

61 Finckenauer & Albanese (2014).

62 Amy Pope (2013), ‘Partnering with Mexico to Fight Transnational Crime’, GPSolo 30(5):66–67.

63 Seelke (2010).

64 Seelke. (2010).

65 José María et al. (2013).

66 John Hatchard (2006).

67 Broadhurst et al. (2014).

68 See https://www.unhcr.org/what-is-a-refugee.html (accessed November 17, 2020). There are different approaches to conceptualize refugees, but a common sense is that refugees are not migrants. For details, see David Scott FitzGerald and Rawan Arar. 2018. “The Sociology of Refugee Migration”, *Annual Review of Sociology* 44: 387–406.

69 Elena et al. (2014)*.*

70 See https://www.unhcr.org/uk/figures-at-a-glance.html (accessed November 17, 2020).

71 Mark Gibney (2010).

72 David Owen. (2018).

73 We assume that the capacity of a country in accommodating imported refugees are related to its economic conditions.

74 Nandini Krishnan et al. (2020).

75 See https://reliefweb.int/report/world/global-refugee-forum-broken-promises-must-end-now (accessed December 8, 2020).

76 See https://www.loc.gov/law/help/refugee-law/sweden.php (accessed December 5, 2020).

77 See https://www.cbsnews.com/news/sweden-rise-of-the-right-immigrants-unwelcome-cbsn-originals/ (accessed December 5, 2020).

78 Cas Mudde (2019)*.*

79 David Scott FitzGerald and Rawan Arar, 2018, The Sociology of Refugee Migration, *Annual Review of Sociology* 44: 387–406.

80 See https://www.unhcr.org/refugee-statistics/ (accessed December 12, 2020).

81 See https://www.un.org/press/en/2018/sc13469.doc.htm (accessed December 14, 2020).

82 See https://www.unrefugees.org/emergencies/syria/ (accessed December 15, 2020).

83 Executive Committee of the High Commissioner, *Update of UNHCR’s Operations in Asia and the Pacific*, 14 September 2018.

84 Paolo Biondi, (2016).

85 See https://www.theguardian.com/world/2018/jun/04/no-entry-hungarys-crackdown-on-helping-refugees.

86 The UN Refugee Agency, 2018, Global Trends: Forced Displacement in 2018, available at: https://www.unhcr.org/globaltrends2018/ (accessed December 17, 2020).

87 Executive Committee of the High Commissioner’s Programme, Update of UNHCR’s operations in Africa, 1–5 October 2018.

88 Libya even run detention centers where to hold returners, refugees and asylum seekers for indefinite periods. See the UN Refugee Agency, UNHCR Position on Returns to Libya (Update II), September 2018.

89 John et al. (2018) Australia’s Refugee Policy: Not a Model for the World, *International Studies* 55 (1): 1–21.

90 Susan Kneebone (2016).

91 For example, the United Nations General Assembly affirmed the “Global Compact on Refugees” in 2018. The Compact provides “a blueprint for governments, international organizations, and other stakeholders to ensure that host communities get the support they need and that refugees can lead productive lives.” See https://www.unhcr.org/gcr/GCR_English.pdf (accessed December 24, 2020).

92 World Bank. (2020).

93 World Bank (2018).

94 See https://www.worldbank.org/en/news/press-release/2020/10/07/covid-19-to-add-as-many-as-150-million-extreme-poor-by-2021 (accessed December 27, 2020).

95 Jonathan Haughton and Shahidur R. Khandker. 2009. Handbook of Poverty and Inequality. Washington, DC: World Bank. P.2.

96 David Brady and Linda M. Burton eds. 2016. Oxford Handbook of the Social Science of Poverty. New York: Oxford University Press.

97 Scholars have argued that some aspects of well-being cannot be priced using a single dimension. Amartya See Sen. 1985. *Commodities and Capabilities*. Amsterdam: North-Holland.

98 Alkire et al. (2014).

99 Individual countries’ international commitment to reduce global poverty in other poor countries and regions is assessed by “humanitarian aid” in our project.

100 For related discussions, see Ferreira, Francisco H. G., and María Ana Lugo. 2013. “Multidimensional Poverty Analysis: Looking for a Middle Ground.” *World Bank Research Observer* 28 (2): 220–35.

101 The R package povcalnetR provides an API for the data retrieving.

102 If there are survey years before and after the missing year, we impute the missing year with linear splines. Suppose $l$ is an income/consumption level. ${y}_{1}$ and ${y}_{2}$ are survey years, and $y$ is our interested missing year, where ${y}_{1}<y<{y}_{2}$. Denote $p(l,y)$ as the proportion of people whose income/consumption is less than $l$ in the year $y$. Then our imputation is $p\left(l,y\right)=\frac{y-{y}_{1}}{{y}_{2}-{y}_{1}} p\left(l,{y}_{1}\right)+\frac{{y}_{2}-y}{{y}_{2}-{y}_{1}}p\left(l,{y}_{2}\right).$ If we only have the survey years on the one side of the missing year, we use GDP as an auxiliary information in the imputation. To be specific, people’s income/consumption is assumed to be linear in the GDP. Suppose that GDP rises by 5%. By our assumption, all people’s income/consumption increase 5%. In this scenario, the whole income/consumption distribution curve shrifts. We impute missing values by calculating poverty statistics from the new distribution.

103 World Bank (2020).

104 Zuo, Cai, Zeng Qingjie & Wang Zhongyuan 2020, *Farewell to Poverty: The Institutional Foundations of Poverty Reduction in China* [Gaobie Pinkun: Jingzhun Fupin De Zhidu Mima], Shanghai: Fudan University Press.

105 See https://www.un.org/press/en/2019/sgsm19779.doc.htm (Accessed December 20, 2020).

106 See http://south.iprcc.org.cn/# (Accessed December 20, 2020).

107 For detailed discussions, see Michelle Adato and John Hoddinott eds. 2010. *Conditional cash transfers in Latin America*. Baltimore, MD: Johns Hopkins University Press; Maria Elisa Balen and Martin Fotta, 2019, *Money from the Government in Latin America: Conditional Cash Transfer Programs and Rural Lives*, London: Routledge.

108 See https://www.un.org/en/sections/issues-depth/population/index.html (Accessed December 25, 2020).

109 World Bank (2020).

110 See https://borgenproject.org/poverty-in-the-solomon-islands/ (Accessed December 24, 2020).

111 See https://www.theguardian.com/world/2005/feb/03/hearafrica05.development (Accessed December 26, 2020).

112 See https://www.worldbank.org/en/news/press-release/2020/10/07/covid-19-to-add-as-many-as-150-million-extreme-poor-by-2021 (Accessed December 26, 2020).

113 United Nations Educational, Scientific and Cultural Organization (UNESCO). (2018). Global education monitoring report 2019: migration, displacement and education: building bridges, not walls.

114 United Nations Educational, Scientific and Cultural Organization (UNESCO). (2018). Global education monitoring report 2019: migration, displacement and education: building bridges, not walls.

115 Hutchings, (2017).

116 Guo et al. (2019).

117 Lee (2013).

118 See https://databank.worldbank.org/source/education-statistics-%5e-all-indicators.

119 In order to distinguish between performance of education in global justice and educational performance itself, we further divided the performance dimension into population-weighted and unweighted performance. We use population-weighted performance, which is finally used for calculating global justice index, to measure the performance of a country in providing educational opportunities for a specific population relative to the world average and unweighted performance to measure the educational performance of a country, regardless of its population size.

120 In the issue of education, we only adopt four indicators of both primary and secondary education from the perspective of educational opportunity. Therefore, China’s high score of unweighted performance does not mean that its basic education quality is comparable to that of the other top 14 countries, but simply means that China ranks high in the world in these four indicators.

121 Ministry of Education of the People’s Republic of China, The National Statistical Bulletin on Education Development in 2018, Stable URL: http://www.moe.gov.cn/jyb_sjzl/sjzl_fztjgb/201907/t20190724_392041.html.

122 Yue et al. (2018).

123 Morgan et al. (2017).

124 The bottom top three countries in education are Pakistan, Ethiopia and India, all of which are far lower than other countries in the score in population-weighted performance dimension. In fact, the scores of India and Pakistan in unweighted performance ranked the 122th and the 161th of 178 countries in 2018. However, the two countries with large population make a considerable proportion of population in the world far behind the world average in terms of access to basic education.

125 UNESCO, 2020, Latin America and the Caribbean Report-Inclusion and Education: All Means All. See https://en.unesco.org/gem-report/LAC2020inclusion.

126 UNESCO, 2020, Latin America and the Caribbean Report-Inclusion and Education: All Means All. See https://en.unesco.org/gem-report/LAC2020inclusion.

127 UNESCO, 2020, Latin America and the Caribbean Report-Inclusion and Education: All Means All. See https://en.unesco.org/gem-report/LAC2020inclusion.

128 Of all regions, sub-Saharan Africa has the highest rates of education exclusion. Over one-fifth of children between the ages of about 6 and 11 are out of school, followed by one-third of youth between the ages of about 12 and 14. According to UIS data, almost 60% of youth between the ages of about 15 and 17 are not in school. See UNESCO’S website: http://uis.unesco.org/en/topic/education-africa.

129 See UNESCO Institute for Statistics (http://uis.unesco.org).

130 We only focus on the opportunities, rather than the qualities of basic education in terms of global justice since there is no systemic data to measure the qualities of basic education of all countries this report covers over years. Evidence suggests that the quality of learning outcomes, a proxy for the quality of basic education, highly correlates with GDP per capita (see Max Roser, Mohamed Nagdy and Esteban Ortiz-Ospina (2013), “Quality of Education”. Published online at OurWorldInData.org. Retrieved from: https://ourworldindata.org/quality-of-education [Online Resource]).

131 For example, Dwyer, J. (2005). Global health and justice. *Bioethics*, 19(5‐6), 460–475. Ruger, J. P. (2009). Global health justice. *Public Health Ethics*, 2(3), 261–275. William W. Fisher and Talha Syed, *Global Justice in Healthcare: Developing Drugs for the Developing World*, 40 U.C. Davis L. Rev. 581 (2006), Available at: http://scholarship.law.berkeley.edu/facpubs/958.

132 The statistics on life expectancy and the under-five mortality are drown from the website of the World Bank. See https://data.worldbank.org/.

133 For example, The International Covenant on Economic, Social and Cultural Rights (ICESCR) proclaims a universal human right to health, namely, the right of everyone to the enjoyment of the highest attainable standard of physical and mental health.

134 Although we believe that NGOs and international organization, such as World Health Organization will play an important role in providing global public health, they are still secondary providers of public health goods.

135 See Ruger (2009).

136 A theory of global health justice always considers public health as a global justice Ruger, J. P. (2009), and Ruger, J. P. (2009). it is difficult to distinguish the contribution of a country from the contribution of other actors, such as international organization and NGOs. Ruger (2009), Dwyer (2005).

137 Gu et al. (2020).

138 See https://www.who.int/nutgrowthdb/database/en/.

139 See https://www.oecd-ilibrary.org/social-issues-migration-health/health-at-a-glance_19991312.

140 OECD (2019).

141 OECD (2019).

142 OECD (2019).

143 Papanicolas et al. (2018).

144 Anderson et al. (2019).

145 The high health-sector prices may explain why the USA’s score in performance dimension is lower than other high-income countries since the USA’s expenditure on public health is far more than others.

146 Papanicolas et al. (2018).

147 OECD (2020).

148 The data comes from WHO Global Health Observatory data repository.

149 World Health Organization. (2020).

150 Sadie Yang and Ao Li (2009).

151 Rebecca (1995).

152 https://womenlobby.org/?lang=en.

153 https://cfc-swc.gc.ca/violence/strategy-strategie/index-en.html.

154 UNDP (2017).

155 Marisa and Amanda (2014).

156 Vichealth (2017).
